# The efficacy of powered toothbrushes: A systematic review and network meta‐analysis

**DOI:** 10.1111/idh.12563

**Published:** 2021-12-31

**Authors:** Tim M. J. A. Thomassen, Fridus G. A. Van der Weijden, Dagmar E. Slot

**Affiliations:** ^1^ Department of Periodontology Academic Centre for Dentistry Amsterdam (ACTA) University of Amsterdam and Vrije Universiteit Amsterdam Amsterdam The Netherlands

**Keywords:** electric, manual, network meta‐analysis, plaque, powered, single brushing, systematic review, toothbrush

## Abstract

**Aim:**

This systematic review and network meta‐analysis synthesizes the available clinical evidence concerning efficacy with respect to plaque scores following a brushing action with oscillating‐rotating (OR) or high‐frequency sonic (HFS) powered toothbrushes (PTB) compared with a manual toothbrush (MTB) as control.

**Material and methods:**

Databases were searched up to 1 August 2021, for clinical trials that evaluated the efficacy of a PTB with OR or HFS technology compared with an MTB on plaque removal after a single‐brushing action and conducted with healthy adult patients. Meta‐analysis (MA) and a network meta‐analysis (NMA) were performed.

**Results:**

Twenty‐eight eligible publications, including 56 relevant comparisons, were retrieved. The overall NMA results for the mean post‐brushing score showed a statistically significant difference for the comparison between an OR PTB and an MTB (SMD = −0.43; 95% CI [−0.696;−0.171]). The change in plaque score data showed a significant effect of a PTB over an MTB and OR over HFS. Based on ranking, the OR PTB was highest, followed by the HFS PTB and the MTB.

**Conclusion:**

Within the limitations of the present study design, based on the outcome following a single‐brushing action, it can be concluded that for dental plaque removal, there is a high certainty for a small effect of a PTB over an MTB. This supports the recommendation to use a powered toothbrush for daily plaque removal. There is moderate certainty for a very small benefit for the use of a powered toothbrush with an OR over an HFS mode of action.

## 1 INTRODUCTION

Dental plaque‐induced gingivitis is an inflammatory lesion resulting from interactions between the biofilm and the host's immune‐inflammatory response. It is reversible by reducing levels of dental plaque at and apical to the gingival margin.^1^, ^2^ Gingivitis is a major risk factor and a necessary prerequisite, for periodontitis. Thus, the management of gingivitis is the primary prevention strategy for periodontitis.^3^, ^4^, ^5^

There are several ways to remove bacterial plaque from teeth, but the use of a toothbrush is considered the most effective.^6^, ^7^, ^8^ The manual toothbrush (MTB) is a relatively simple device that is widely accepted and affordable to most people.^5^ Powered toothbrushes (PTB) were first introduced in the 1940s, starting with devices with a circular brush head and a straight brush head. The first generation of electric toothbrushes was essentially mechanized versions of manual toothbrushes, with the bristles moving back and forth in an imitation of how people brush by hand. Over the years, design changes have increased the efficacy of powered toothbrushes in plaque removal, including improved brush head and filament arrangement architecture,^9^, ^10^ increased motion^11^ and compliance‐enhancing features.^12^ Modern innovations such as mobile apps give people easy access to oral health knowledge, further improving levels of oral hygiene.^13^ These developments over the years have resulted in various types of PTBs with different brush head configurations and modes of action. Currently, the PTBs with oscillating‐rotating (OR) and high‐frequency sonic (HFS) technology are the most common commercially available products on the market globally.^14^

Various systematic reviews have evaluated the efficacy of MTBs and PTBs. In general, they conclude that PTBs are more effective than MTBs in reducing dental plaque, gingivitis and bleeding.^15^ Previously, it has been shown that following brushing with an MTB, an average 42% plaque score reduction can be expected.^16^ A similar review^14^ that evaluated PTBs found an average plaque score reduction of 46%. A more recent review comparing the PTB and the MTB concluded that there is moderate certainty that a PTB is more effective than an MTB for plaque removal.^17^ The most recent systematic review evaluated OR and HFS PTBs in particular and concluded that there is moderate certainty of a significant but very small beneficial effect in favour of OR.^18^ These studies all evaluate the effect following a single‐brushing action and do not consider the benefits of gingival health. They are, however, appropriate for estimating the potential plaque removal,^19^ as they facilitate the control of confounding variables such as patient compliance.^20^

A Cochrane systematic review (SR) from almost a decade ago involved a direct comparison between PTBs with different modes of action on plaque score reduction.^21^ At that time, no definitive conclusions could be drawn regarding the superiority of one particular type of PTB over another. However, some evidence showed that the OR PTBs reduce plaque more than HFS PTBs in the short term.^21^ A recently updated SR using the same methodology concluded that the evidence does not suggest the superiority of either OR or HFS PTBs for reducing plaque or gingivitis scores.^22^ Based on studies conducted over the last decade, a recent SR compared the efficacy of OR and other PTBs and concluded that there is evidence to suggest that OR is more efficient in plaque removal and reduction in the number of bleeding sites than other PTBs, including HFS.^23^

It is the dental care professional (DCP)’s role to provide oral hygiene advice to their patients based on the best available evidence.^24^ Thus, evidence‐based findings concerning the mechanical plaque removal of a toothbrush have to be established and made readily available. In this respect, an NMA combines both indirect and direct evidence providing the most precise estimate of treatment effects to support decision‐making.^25^, ^26^, ^27^ The simultaneous comparison of all interventions of interest in the same analysis enables the estimation of their relative ranking for a given outcome. Recently, an NMA^28^ was published that only evaluated studies obtained from the database of a PTB manufacturer covering the period 2007–2017. It solely addressed RCTs with a duration up to three months. As the studies were retrieved from a non‐public archive, with in addition a non‐transparent search strategy and extraction of data, this review had considerable limitations. Therefore, the purpose of the present study is to systematically evaluate the available clinical evidence concerning efficacy with respect to plaque scores following a brushing action with OR or HFS PTBs compared with an MTB as control and synthesize this with an NMA.

## 2 MATERIALS AND METHODS

This review is prepared and reported in accordance with the Cochrane Handbook for Systematic Reviews of Interventions.^29^ In addition, the guidelines of Preferred Reporting Items for Systematic Reviews and Meta‐Analyses (PRISMA)^30^ were followed and the consequent extensions for abstracts^31^, ^32^ and Network Meta‐Analyses (NMA).^33^ The protocol that details the review method was developed *a priori* after an initial discussion among the members of the research team. The review is registered under number CRD42020192418 with the International Prospective Register of Systematic Reviews (PROSPERO).^34^ The study was also approved by the Medical Ethical Committee of the Academic Center for Dentistry Amsterdam (ACTA‐ETC), the Netherlands, under number: 2021‐1835.

### 2.1 Focussed PICOS question

The PICOS question^35^ for this review is: Based on (randomized) controlled clinical trials, what is the efficacy of a PTB with an OR or a HFS technology as compared to an MTB on dental plaque removal following a single‐brushing action in healthy participants?

### 2.2 Search strategy

A structured and comprehensive search strategy was designed to retrieve all relevant publications that satisfied the study purpose with a direct comparison between:

● MTB and OR
● MTB and HFS
● HFS and OR

Electronic databases were searched for relevant papers. These included The National Library of Medicine, Washington, D.C. (MEDLINE‐PubMed); and the Cochrane Central Register of Controlled Trials (CENTRAL). The last electronic search was performed on 1 August 2021. The search strategy and employed search terms and keywords are presented in Table 1. All references cited in the papers selected for this review were checked for additional potentially suitable studies. Hand searching was only performed as part of the Cochrane Worldwide Hand Searching Program uploaded to CENTRAL.

**TABLE 1:** Search terms used for MEDLINE‐PubMed and Cochrane‐CENTRAL. The search strategy was customized according to the database being searched. The following strategy was used in the search

{(<intervention AND outcome>)} {<[(MeSH terms) Toothbrushing OR (text words) toothbrush OR toothbrushing OR toothbrush*> AND <(MeSH terms) dental plaque OR dental plaque index OR dental deposits OR [text words] plaque OR dental plaque OR plaque removal OR plaque index OR dental plaque removal OR dental deposit* OR dental deposits* OR dental deposit OR dental deposits>}

Note

The asterisk (*) was used as a truncation symbol.

### 2.3 Screening and selection

Titles and abstracts of the studies obtained from the searches were screened in detail for suitability by two reviewers (TMJAT, DES) using the Rayyan^36^ web application. The reviewers worked independently and were blinded for each other's results during the screening process. Possible duplicates were identified and checked to eliminate those that were identical. Disagreements in the screening and selection process were resolved by consensus or, if disagreement persisted, by arbitration through a third reviewer (GAW) until consensus was reached. The papers that fulfilled all of the inclusion criteria were processed for data extraction.

Studies were deemed eligible for inclusion if they conformed to the following criteria:

● Publications written in the English language
● (Randomized) Controlled Clinical Trials (CCT or RCT)
● Studies conducted with human participants:
  - ≥18 years old.
  - In good general health (without systemic disorder or pregnancy)
  - Without diagnosed periodontitis
  - Without orthodontic fixed appliance and/or removable prosthesis
  - Without dental implants
● Intervention; powered toothbrush, technologies of interest being oscillating‐rotating and high‐frequency sonic
● Comparison; manual toothbrush
● All toothbrushes must be single‐headed
● Self‐performed brushing by the participant
● Single‐brushing action
● Full‐mouth plaque scores assessed according to one of the following most commonly used plaque indices or their modification:
  - Quigley and Hein plaque index (Q&HPI^37^ or the Turesky^38^ modification assessed at two sites per tooth or according to the Lobene^39^ modification up to six sites per tooth).
  - Navy plaque index^40^ or Rustogi modified Navy plaque index (RMNPI)^41^
● Plaque score data available as mean and standard deviation (SD) for pre‐ and post‐brushing and/or incremental plaque score reduction.

### 2.4 Heterogeneity assessment

Across the studies, the factors used to evaluate the clinical and methodological heterogeneity of the characteristics of the different studies were as follows: study design and evaluation period, subject characteristics, brushing regimen, technology of mode of action, instructions given and plaque indices or their modifications.

As part of the NMA, heterogeneity was statistically tested by the I^2^ statistic, which describes the percentage of variation across studies due to heterogeneity rather than chance. As a rough guide, I^2^ was interpreted as follows: an I^2^ of 0%–40% may indicate unimportant levels of heterogeneity; an I^2^ of 30%–60% may represent moderate heterogeneity; an I^2^ of 50%–90% may represent substantial heterogeneity; and an I^2^ greater than 75% may indicate considerable heterogeneity.^42^

### 2.5 Risk of bias and (in)directness

Two reviewers (TMJAT and DES) individually scored the methodological qualities of the included studies according to the method described by Van der Weijden et al.,^43^ and in greater detail by Keukenmeester et al.^44^ In summary, the study was classified as having an estimated ‘low risk of bias’ when random allocation, defined eligibility criteria, masking of examiners, masking of patients, balanced experimental groups, identical treatment between groups (except for the intervention) and reporting of follow‐up were present. The study was considered to have an estimated ‘moderate risk of bias’ when one of these seven criteria was missing. When two or more of these criteria were missing, the study was estimated to have a ‘high risk of bias’. The potential risk of bias was estimated, and the acquired evidence was graded.

For the assessment of indirectness in the context of the NMA two components were considered: the similarity of the studies in the analysis to the target PICO‐question^35^ (i.e. the extent to which the evidence relates to the population, intervention(s), comparisons and outcomes of interest); and the transitivity assumption, which is the comparison between two treatments via a third one.^45^

For the present review, risk of bias and the assessment of indirectness were checked for each included study by two reviewers (TMJAT and DES). If disagreements in the quality assessments were found, this was resolved by consensus after discussion.

### 2.6 Statistical analysis

#### 2.6.1 Data extraction

The data from the publications that met the selection criteria were extracted and processed for further analysis. Custom‐designed data extraction forms were used by two independent reviewers (TMJAT and DES) for mean pre‐ and post‐brushing and incremental plaque score data and SD. If studies provided a standard error (SE) of the mean, these values were converted to SD based on the sample size (SE = SD/√N). In all cases, to ensure an accurate estimate, any data approximation in figures was avoided. In case of missing data or undetermined information, attempts were made to contact the first or corresponding author of the included publications for clarification or to retrieve additional data. For studies with multiple treatment arms, and for those in which data from the control group were compared with more than one other group, the number of participants (N) in the control group was divided by the number of comparisons.^46^ Disagreements in the data extraction were resolved by discussion and consensus.

#### 2.6.2 Data analysis

##### Network meta‐analysis

From the selected papers, the mean plaque scores, the standard deviations and the number of participants per group for the consequent plaque scores were used for the NMA. The NMA was performed using MetaInsight^47^ (with either the fixed‐ or random‐effects model, as appropriate). MetaInsight^47^ is an interactive web‐based tool for analysing, interrogating, and visualizing network meta‐analyses using R‐shiny and netmeta (see: https://crsu.shinyapps.io/MetaInsight/).^48^ Irrespective of which plaque index score the data were related to, the overall effect size analysis was calculated as the standardized mean difference (SMD). The difference of means (DiffM) was used for the sub‐analysis per plaque index score. The 95% confidence interval (CI) is presented for both the SMD and the DiffM. The NMA was performed on overall data as well as for the direct and indirect comparison. In addition, the 95% prediction interval (PI) was calculated.

Treatments were ranked based on the NMA and ranking was performed by P‐scores. The P‐scores are based solely on the point estimates and standard errors of the network estimates. They measure the extent of certainty that one treatment is better than another, averaged over all the competing treatments.^49^ This interpretation is comparable to that of the surface under the cumulative ranking curve (SUCRA), which is the rank of a treatment within the range of treatments, measured on a scale from 0 (worst) to 1 (best).^50^

### 2.7 Evidence profile

The Grading of Recommendations Assessment, Development and Evaluation (GRADE) system, as proposed by the GRADE working group,^51^ was used to rank the body of evidence emerging from this review. Two reviewers (TMJAT and DES) used the GRADE^51^ approach and the Confidence in Network Meta‐Analysis Software (CINeMA)^52^, ^53^ to evaluate the strength of evidence for results at the end of treatment from the NMA. CINeMA is a web application that simplifies the evaluation of confidence in the findings from an NMA and is based on the framework developed by Salanti et al.^25^ and refined by Nikolakopoulou et al.^53^ It is a single page application that communicates to an R back‐end server. In particular, the package's meta and netmeta are used.^54^ The CINeMA platform provides a transparent framework to evaluate evidence from systematic reviews with multiple interventions.^25^ Six domains that affect the level of confidence in the NMA results are considered: (i) within‐study bias, (ii) across‐studies bias, (iii) indirectness, (iv) imprecision, (v) heterogeneity and (vi) incoherence. Any disagreement between the two reviewers was resolved after additional discussion with the third reviewer (GAW).

## 3 RESULTS

### 3.1 Search and selection results

**FIGURE 1:**
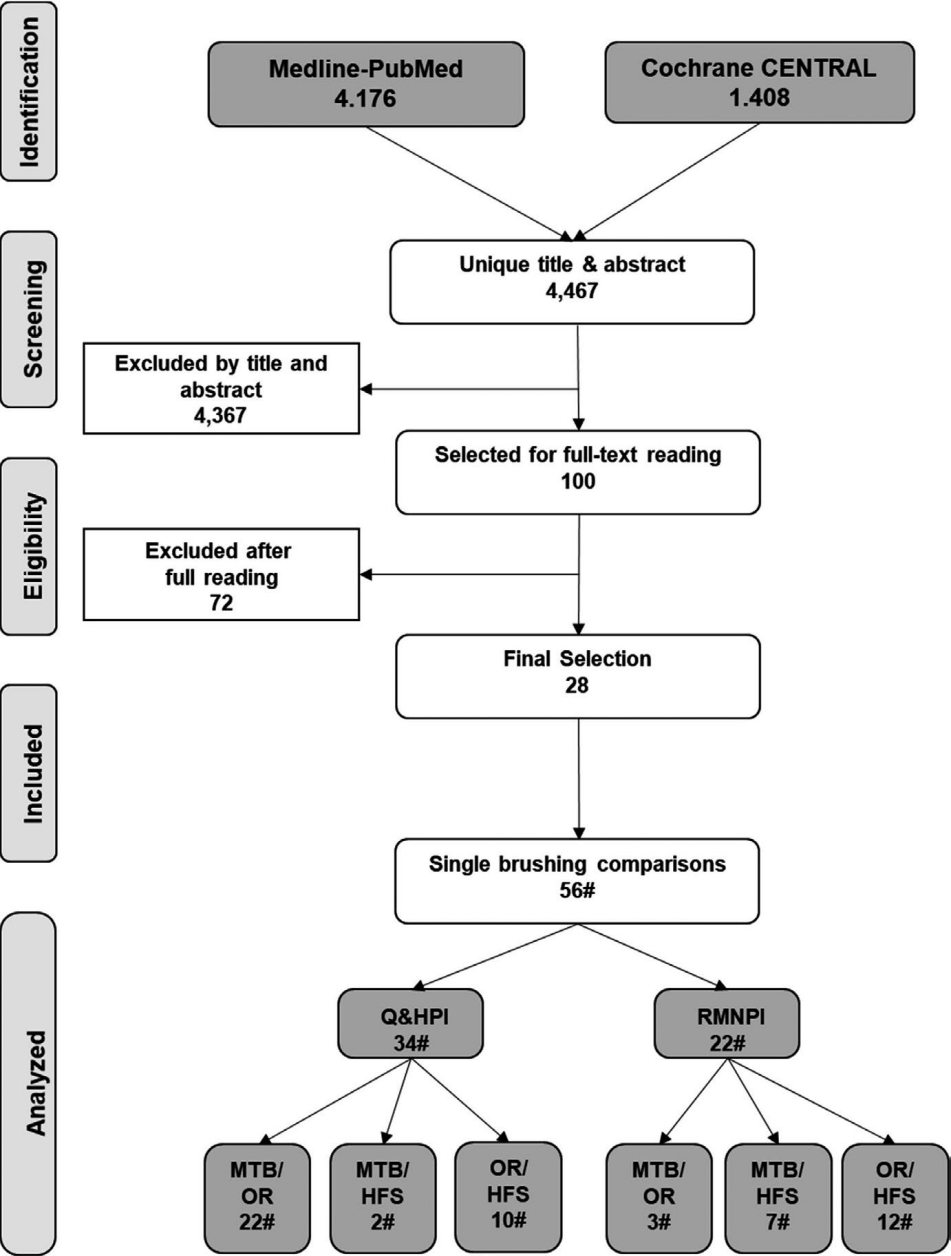
Search and selection results

A search of the MEDLINE‐PubMed and Cochrane‐CENTRAL databases identified 4467 unique papers (for details, see Figure 1). Screening the titles and abstracts resulted in 100 papers, which were obtained in full text. After careful, extensive and detailed reading, 72 papers were excluded (for details, see online Appendix S1). This resulted in 28^9^, ^28^, ^55^, ^56^, ^57^, ^58^, ^59^, ^60^, ^61^, ^62^, ^63^, ^64^, ^65^, ^66^, ^67^, ^68^, ^69^, ^70^, ^71^, ^72^, ^73^, ^74^, ^75^, ^76^, ^77^, ^78^ papers for inclusion in this review, describing in total 56 comparisons. Of these, 34 comparisons used the Q&HPI^37^ or a modification,^38^, ^39^ and 22 used the RMNPI.^40^, ^41^ In total, 25 compared the MTB to OR PTBs, nine compared the MTB to HFS PTBs, and 22 compared the HFS to OR PTBs.

### 3.2 Study characteristics

All but one^70^ of the 28 selected studies were RCTs. The characteristics of each study are displayed in online appendix Table S2, and the extracted data are presented by means and SD separately per plaque index of interest (online appendix, Table S3a‐b). The number of participants varied from 12 to 216 per group, and the age ranged from 18 to 69. In 14 studies,^55^, ^57^, ^58^, ^59^, ^63^, ^65^, ^67^, ^72^, ^75^, ^78^, ^79^, ^80^, ^81^ a familiarization phase of 2 days up to 6 weeks prior to the single‐brushing action was part of the research protocol. Instructions were given in a written format in 22 studies,^9^, ^56^, ^57^, ^58^, ^59^, ^60^, ^61^, ^62^, ^64^, ^67^, ^68^, ^72^, ^73^, ^74^, ^75^, ^76^, ^78^, ^79^, ^80^, ^81^, ^82^ five studies^55^, ^63^, ^65^, ^77^, ^81^ provided visual instructions to their participants and one study^70^ did not give any instruction (for details, see online appendix Table S2).

### 3.3 Risk of bias and (in)directness

A summary of the evaluation of the risk of bias in the individual studies is shown in Table S3a‐b in the online appendix, along with the indirectness assessment. Of the 28 selected studies, 24 were estimated to have a low risk of bias, three a moderate risk and one a high risk (online appendix, Table S4). The indirectness was scored low for 17 and moderate for 11 comparisons.

### 3.4 Synthesis of the results

#### 3.4.1 Meta‐analysis

At pre‐brushing, no statistically significant difference between the comparisons was found (OR vs. MTB, SMD = 0.10; 95% CI (0.00;0.19); HFS vs. MTB, SMD = 0.08; 95% CI (−0.04;0.19)) (for details, see online appendix Tables S5, S6 and S7). Tables 2 and 3 present an overview of the outcomes of the performed NMA on post‐brushing plaque scores, and Tables 4 and 5 show the incremental reduction in plaque scores. An overall analysis and a sub‐analysis per plaque index score are presented. The corresponding forest plots are displayed in the online appendices (S8 through S13).

**TABLE 2:** Meta‐analysis for the standardized mean difference (SMD) evaluating efficacy of a manual toothbrush (MTB), an oscillating‐rotating powered toothbrush (OR) and a high‐frequency sonic powered toothbrush (HFS) using the MQ&HPI and the RMNPI. Overall results, independent of the plaque indices used. *Post*‐*brushing data*

	Single‐brushing design *Post‐brushing*	Number of comparisons	Network meta‐analysis				Effect size			Heterogeneity		Online appendix number
			NMA (95% CI)	Indirect (95% CI)	Direct (95% CI)	95%PI	SMD	95% CI	*p*‐value	*I^2^* Statistic *^%^*	*t^2^*	Forest Plot
*Modified Quigley & Hein PI +* *Rustogi Modified Navy PI*	HFS:MTB	9	−0.27 (−0.586;0.040)	0.16 (−0.269;0.593)	−0.77* (−1.235;−0.313)	−1.604;1.055	−0.94	−1.57;−0.31	0.00	85.0	0.469	S8a‐c
	HFS:OR	18	0.16 (−0.112;0.430)	−0.55 (−1.097;0.000)	0.39* (0.076;0.699)	−1.161;1.478	0.94	0.31;1.57	0.00	92.1	0.444	S8a‐c
	OR:MTB	22	−0.43* (−0.696;−0.171)	−1.16* (−1.718;−0.605)	−0.23 (−0.523;0.071)	−1.751;0.884	0.94	0.31;1.57	0.00	75.1	0.191	S8a‐c

Abbreviations: CI, confidence interval; PI, prediction interval.

* Statistically significant.

**TABLE 3:** Meta‐analysis for the difference of means (DiffM) evaluating efficacy of a manual toothbrush (MTB), an oscillating‐rotating power toothbrush (OR) and a high‐frequency sonic powered toothbrush (HFS) using the MQ&HPI and the RMNPI. Sub‐analysis per index. *Post*‐*brushing data*

	Single‐brushing design *Post‐brushing*	Number of comparisons	Network meta‐analysis				Effect size			Heterogeneity		Online appendix number
			NMA (95% CI)	Indirect (95% CI)	Direct (95% CI)	95%PI	DiffM	95% CI	*p*‐value	*I^2^* Statistic *^%^*	*t^2^*	Forest Plot
*Modified Quigley & Hein PI*	HFS:MTB	2	−0.06 (−0.141;0.012)	−0.06 (−0.150;0.021)	−0.06 (−0.235;0.107)	−0.176;0.047	0.00	−0.19; 0.19	1.00	0.0	0.000	S9a‐c
	HFS:OR	9	−0.00 (−0.069;0.063)	−0.000 (−0.180;0.175)	−0.00 (−0.075;0.068)	−0.108;0.101	−0.00	−0.19; 0.19	1.00	11.7	0.001	S9a‐c
	OR:MTB	21	−0.06 (−0.106;0.016)	−0.06 (−0.246;0.125)	−0.06* (−0.108;−0.014)	−0.153;0.030	−0.00	−0.19;0.19	1.00	18.2	0.002	S9a‐c
*Rustogi Modified Navy PI*	HFS:MTB	7	−0.08* (−0.115;−0.037)	−0.06 (−0.172;0.045)	−0.08* (−0.120;−0.036)	−0.194;0.043	−0.01	−0.13;0.10	0.81	86.9	0.003	S10a‐c
	HFS:OR	9	0.05* (0.022,0.088)	0.04 (−0.070;0.152)	0.06* (0.021;0.091)	−0.062;0.171	0.01	−0.10;0.13	0.81	95.9	0.003	S10a‐c
	OR:MTB	1	−0.13* (−0.178;−0.130)	−0.13* (−0.188;−0.079)	−0.12* (−0.222;−0.016)	−0.253;−0.008	0.01	−0.10;0.13	0.81	na	na	S10a‐c

Abbreviations: CI, confidence interval; na; Not applicable; PI, prediction interval.

* Statistically significant.

**TABLE 4:** Meta‐analysis for the standardized mean difference (SMD) evaluating efficacy of a manual toothbrush (MTB), an oscillating‐rotating power toothbrush (OR) and a high‐frequency sonic power toothbrush (HFS) using the MQ&HPI and the RMNPI. Overall results, independent of the plaque indices used. *Incremental change between pre*‐ *and post*‐*brushing*

	Single‐brushing design *Difference*	Number of comparisons	Network meta‐analysis				Effect size			Heterogeneity		Online appendix number
			NMA (95% CI)	Indirect (95% CI)	Direct (95% CI)	95%PI	SMD	95% CI	*p*‐value	*I^2^* Statistic *^%^*	*t^2^*	Forrest Plot
*Modified Quigley & Hein PI +* *Rustogi Modified Navy PI*	HFS:MTB	8	−0.60* (−0.980;−0.214)	−0.04 (−0.570;0.493)	−1.20* (−1.753;−0.648)	−2.138;0.944	−1.16	−1.93;−0.40	0.00	87.6	0.641	S11a‐c
	HFS:OR	16	0.47* (0.148;0.800)	−0.41 (−1.084;0.256)	0.75* (0.376;1.121)	−1.053;2.001	1.16	0.40;1.93	0.00	94.7	0.619	S11a‐c
	OR:MTB	16	−1.07* (−1.401;−0.742)	−1.95* (−2.616;−1.283)	−0.76* (−1.166;−0.408)	−−0.457;2.599	1.16	0.40;1.93	0.00	87.2	0.256	S11a‐c

Abbreviations: CI, confidence interval; na; Not applicable; PI, prediction interval.

* Statistically significant.

**TABLE 5:** Meta‐analysis for the difference of means (DiffM) evaluating efficacy of a manual toothbrush (MTB), an oscillating‐rotating power toothbrush (OR) and a high‐frequency sonic power toothbrush (HFS) using the MQ&HPI and the RMNPI. Sub‐analysis per index. *Incremental change between pre*‐ *and post*‐*brushing*

	Single‐brushing design *Difference*	Number of comparisons	Network meta‐analysis				Effect size			Heterogeneity		Online appendix number
			NMA (95% CI)	Indirect (95% CI)	Direct (95% CI)	95%PI	DiffM	95% CI	*p*‐value	*I^2^* Statistic *^%^*	*t^2^*	Forrest Plot
*Modified Quigley & Hein PI*	HFS:MTB	1	−0.14* (−0.261;−0.026)	−0.14* (−0.279;−0.011)	−0.14 (−0.380;0.100)	−0.396;0.108	0.00	−0.27; 0.28	0.97	na	na	S12a‐c
	HFS:OR	4	0.04 (−0.068;0.141)	0.04 (−0.210;0.291)	0.04 (−0.079;0.151)	−0.209;0.282	−0.00	−0.28; 0.27	0.97	0.0	0.000	S12a‐c
	OR:MTB	13	−0.18* (−0.247;−0.113)	−0.18 (−0.442;0.091)	−0.18 (−0.442;0.091)	−0.410;0.049	−0.00	−0.28;0.27	0.97	56.9	0.005	S12a‐c
*Rustogi Modified Navy PI*	HFS:MTB	7	−0.08* (−0.110;−0.043)	−0.02 (−0.087;0.038)	−0.10* (−0.137;−0.058)	−0.184;0.031	−0.07	−0.015;0.00	0.05	90.9	0.004	S13a‐c
	HFS:OR	12	0.05* (0.027;0.079)	−0.01 (−0.078;0.058)	0.06* (0.035;0.091)	−0.052;0.158	0.07	−0.00;0.15	0.05	95.6	0.002	S13a‐c
	OR:MTB	3	−0.13* (−0.166;−0.092)	−0.16* (−0.209;−0.112)	−0.09* (−0.143;−0.032)	−0.237;−0.021	0.07	−0.00;0.15	0.05	94.4	0.001	S13a‐c

Abbreviations: CI, confidence interval; na; Not applicable; PI, prediction interval.

* Statistically significant.

Only for the post‐brushing scores of the comparison between OR and MTB did the overall NMA show a statistically significant difference (SMD = −0.43; 95% CI (−0.696;−0.171)) (Table 2). The sub‐analysis (Table 3) based on the (M)Q&HPI^37^, ^38^, ^39^ did not show any significant differences between the three types of toothbrushes. This result was in contrast to the RMNPI,^40^, ^41^ which showed a significant effect in favour of the PTBs when compared to the MTB. There was a significant difference between the two PTB technologies, favouring OR (DiffM = 0.05; 95% CI (0.022;0.088)).

Analysis of the outcomes on incremental plaque score data (Table 4) showed a significant reduction for all three comparisons of interest. In detail, the two types of PTBs present both a significant difference compared to an MTB, and OR shows a significant difference in comparison with HFS. The sub‐analysis per plaque index score (Table 5) shows that only when (M)Q&HPI^37^, ^38^, ^39^ was used could no difference be found between the PTB technologies (DiffM = 0.04; 95% CI (−0.068;0.141)).

### 3.5 Heterogeneity assessment

The studies included in the NMA showed considerable heterogeneity,^29^ as I^2^ statistic values range from 76.3% and 94.7% (Tables 2 and 4). The sub‐analysis per plaque index score shows an unimportant to moderate heterogeneity (0.0%–56.9%) for (M)Q&HPI^37^, ^38^, ^39^ and considerable heterogeneity (86.9%–95.9%) for RMNPI^40^, ^41^ NMA (Tables 3 and 5).

### 3.6 Network meta‐analysis graph

**FIGURE 2:**
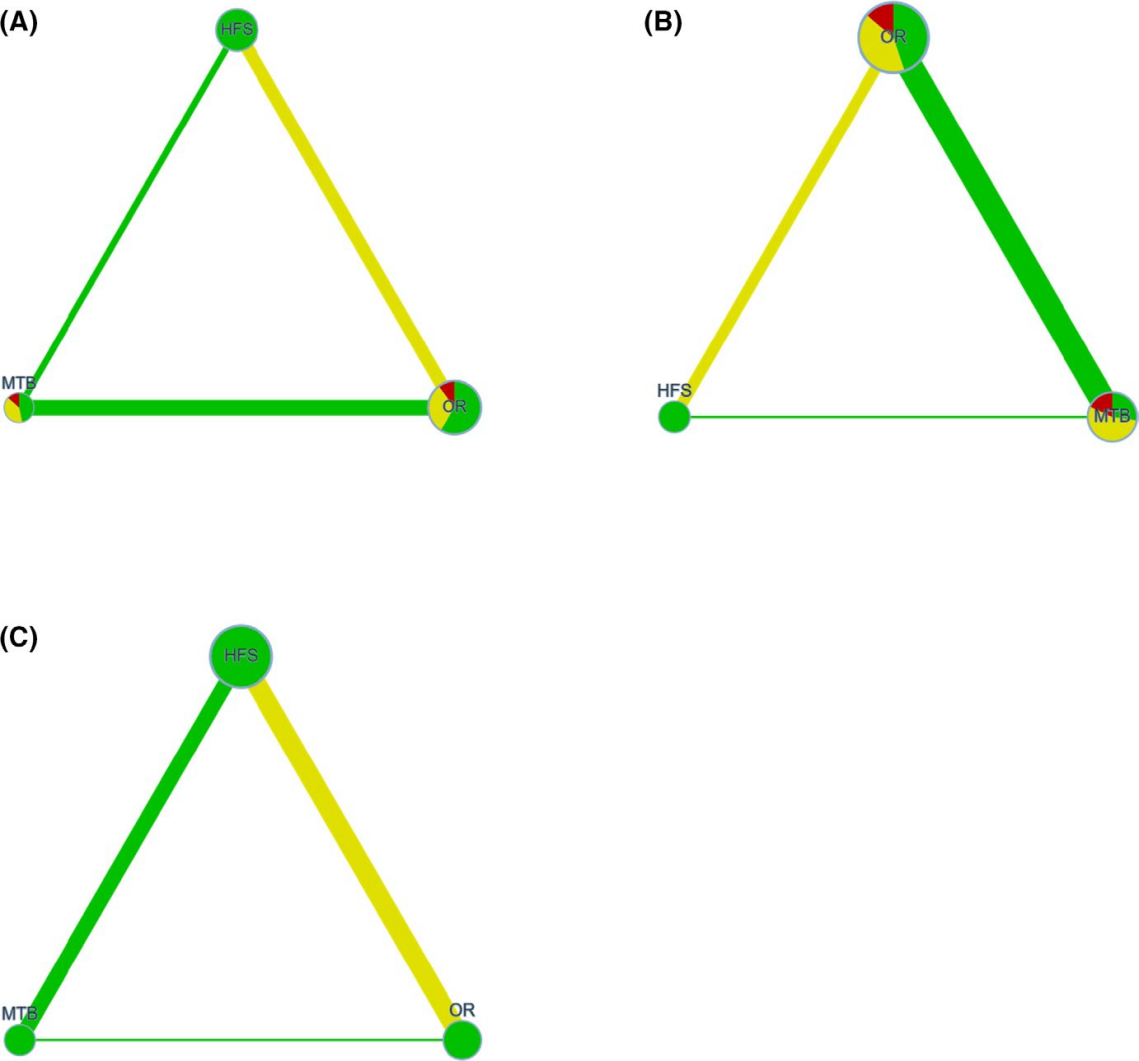
Confidence in Network Meta‐Analysis (CINeMA). The different nodes represent a device, manual toothbrush (MTB), an oscillating‐rotating power toothbrush (OR) and a high‐frequency sonic power toothbrush (HFS). The colour of the node represents the risk of bias and the size of the node the sample size. The width of the edge shows the number of included studies and the colour the average indirectness. *Post*‐*Brushing*. (A) Network meta‐analysis graph irrespective of the plaque indices. (B) Network meta‐analysis graph using only the Q&HPI. (C) Network meta‐analysis graph using only the RMNPI

**FIGURE 3:**
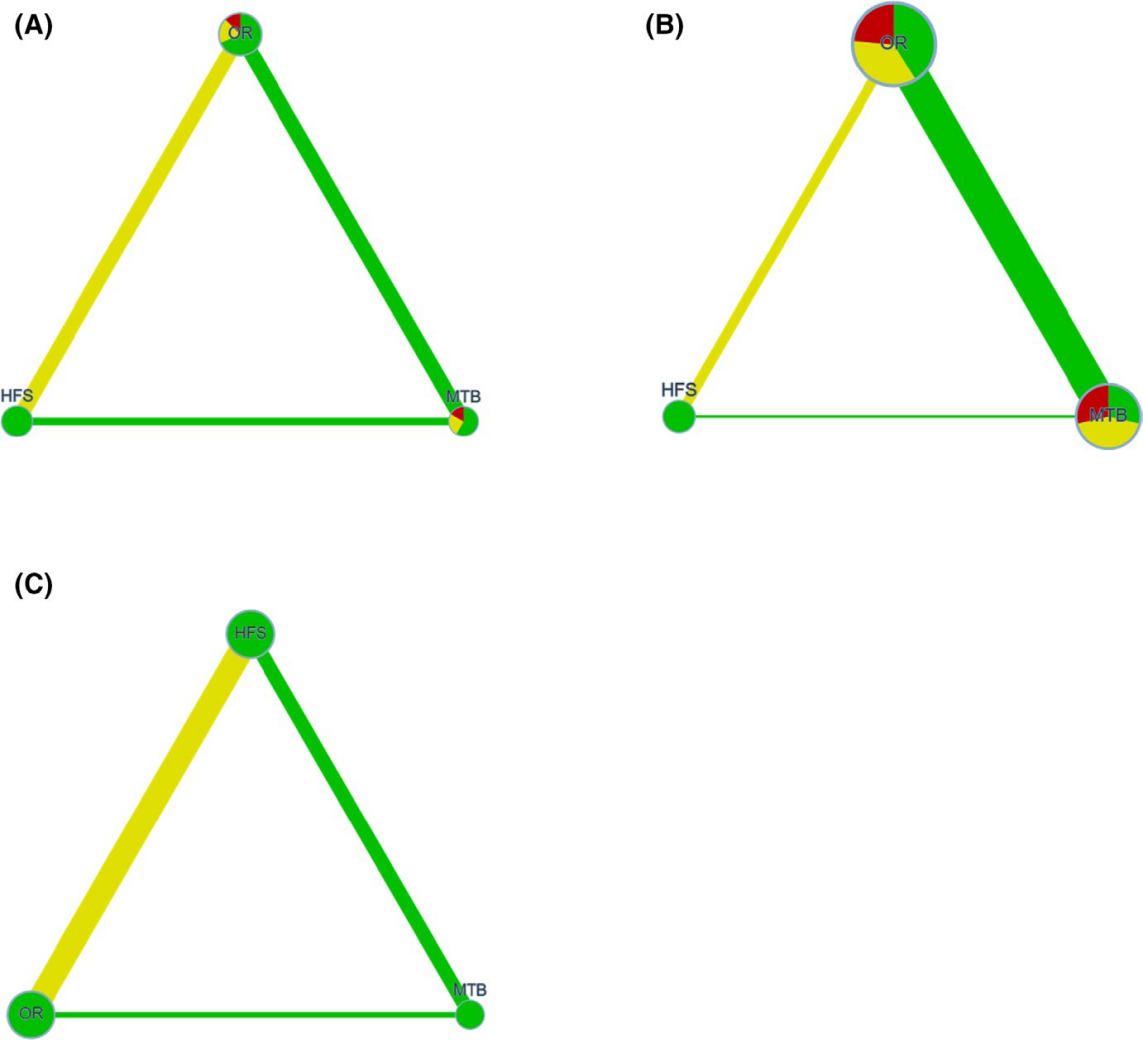
Confidence in Network Meta‐Analysis (CINeMA). The different nodes represent a device, manual toothbrush (MTB), an oscillating‐rotating power toothbrush (OR) and a high‐frequency sonic power toothbrush (HFS). The colour of the node represents the risk of bias and the size of the node the sample size. The width of the edge represents the number of included comparisons and the colour the average indirectness. Incremental change between pre‐ and post‐brushing. (A) Network meta‐analysis graph irrespective of the plaque indices. (B) Network meta‐analysis graph using only the Q&HPI. (C) Network meta‐analysis graph using only the RMNPI

The graphs of the NMA^53^ for post‐brushing scores are shown in Figure 2A‐C, and those for the incremental reduction in plaque scores are shown in Figure 3A‐C for the overall and subsequent sub‐analyses. It provides a visual synthesis of the evidence comparing the MTB and the two different PTB modes of action (HFS and OR). The different nodes represent a device, risk of bias and sample size. The width of the edge represents the number of included comparisons and the indirectness (for details, see Appendices S14 and S15).

### 3.7 Confidence in network meta‐analysis

The six domains that affect the level of confidence in the NMA were estimated with CINeMA^53^ (see Appendices S16–S21). Based on the main concerns regarding heterogeneity and incoherence, a moderate confidence rating of the overall NMA for both post‐brushing and incremental reduction in plaque index scores was present.

### 3.8 Ranking of interventions

When the toothbrushes are ranked based on the P‐scores as result of the NMA by the program MetaInsight^47^, either based on post‐brushing (Table 6) or incremental change (Table 7) in plaque scores, the OR ranks first and MTB last. The exception is the sub‐analysis for the post‐brushing scores when (M)Q&HPI^37^, ^38^, ^39^ is used, where HFS ranks first. For details on ranking data, see online appendices S8–S13.

**TABLE 6:** Ranking table. *Post*‐*Brushing*

Rank *Post‐Brushing*	1	2	3	Online Appendix number
Overall	OR	HFS	MTB	S8b,c
(M)Q&HPI	HFS	OR	MTB	S9b‐d
RMNPI	OR	HFS	MTB	S10b‐d

**TABLE 7:** Ranking table. *Incremental reduction between pre*‐ *and post*‐*brushing*

Rank *Difference*	1	2	3	Online Appendix number
Overall	OR	HFS	MTB	S11b,c
(M)Q&HPI	OR	HFS	MTB	S12b‐d
RMNPI	OR	HFS	MTB	S13b‐d

### 3.9 Evidence profile

Table 8 shows the evidence profile based on a summary of the various factors used to rate the quality of evidence and the level of certainty. It accumulates into an estimation of the strength and direction of the recommendation according to GRADE.^49^ With respect to removal of dental plaque, there is a high certainty for a small effect, which supports the recommendation to advise using a PTB rather than an MTB. In addition, there is moderate certainty for a very small beneficial effect for the use of an OR mode of action PTB over a HFS PTB.

**TABLE 8:** Estimated evidence profile appraisal of the strength of the recommendation and the direction regarding the use of the different toothbrushes

Determinants of the quality	In majority based on	Plaque scores
Study design	Appendix S2	RCT/CCT
# Studies	Figure 1	#28
# Comparisons	Figure 1	#56
Risk of Bias	Appendix S4	Low to High
Consistency	Table 2‐7	Rather consistent
Directness	Single‐brushing design	Rather generalizable
Precision	Table 2, 3, 4, 5	Precise
Reporting Bias	Appendix S16–21	Possible to Likely
Magnitude of the effect	PTB vs MTB HFS vs OR	Small Very small
Strength of the recommendation based on the quality and body of evidence	PTB vs MTB HFS vs OR	Strong Moderate

Note

Strength and direction of the recommendation: With respect to removal of dental plaque, there is high certainty for a small effect of a PTB over an MTB which is in support of a recommendation to advice a PTB over an MTB. There is moderate certainty for a very small benefit for the use of an OR mode of action PTB over a HFS PTB.

## 4 DISCUSSION

### 4.1 Network meta‐analysis

The aim of this study was to use an NMA to systematically compare and rank the effect of two different PTBs (OR and HFS technology) compared with an MTB with respect to plaque removal as evaluated following a single‐brushing action research model. An NMA is a novel approach that takes the assumptions of a MA one step further.^84^ An NMA^29^ incorporates direct and indirect comparisons based on the principle of transitivity, which relies on the fact that combined studies have a common comparator.^85^ The addition of indirect comparisons by incorporating evidence from other sources makes the results more robust.^86^ When the network is well connected and provides both direct and indirect comparisons, these can be pooled together into ‘mixed evidence’, which increases statistical power and the precision of the estimates.^84^ The use of added information also allows for more robust recommendations compared with conventional pairwise meta‐analyses.^87^, ^88^ Consequently, a new evidence hierarchy is proposed with the NMA at the top of the pyramid of evidence, followed by the pairwise MA and SRs without NMA or MA.^88^

From this review, it can be concluded that when combining direct and indirect evidence, there is a significant difference in plaque score reduction after a single‐brushing action in favour of both PTB technologies compared with an MTB. In addition, there is moderate certainty for a very small benefit for the use of an OR PTB mode of action over a HFS PTB. The ranking supports these findings, and this also concurs with the ranking in a recently published NMA by Grender et al. (2020),^28^ based on studies with a three‐month duration. The results of the present review are congruent with the findings of the recent evaluation of single‐brushing actions^17^ and the Cochrane SR,^89^ which were both pairwise comparisons of PTBs and MTBs.

### 4.2 Plaque indices

From previous reviews on toothbrushing efficacy that have evaluated plaque reduction following a single act of brushing,^14^, ^16^, ^17^, ^18^ it is apparent that the indices most frequently used are Q&HPI^37^, ^38^, ^39^ and the RMNPI^40^, ^41^ and their modifications. Based on this observation, it was decided that these indices would be used as parameters of interest for the present review. This decision mirrors the approach of Elkerbout et al. (2019),^17^ who restricted their selection of papers to those that provided outcomes related to these two indices. PTB manufacturers of the different modes of action apparently use various plaque indices to evaluate the efficacy of their products.^17^ The choice of the plaque score index is presumably related to the manufacturer's preference or a research facility's expertise. Furthermore, in this NMA, most PTB studies evaluating the HFS mode of action assessed plaque according to the criteria of the RMNPI.^40^, ^41^ Conversely, with an OR mode of action, most evaluations are based on the Q&HPI.^37^, ^38^, ^39^ This phenomenon may contribute to reporting bias. In our earlier work,^14^, ^16^ we have shown that the outcomes with the Q&HPI^37^, ^38^, ^39^ result in a smaller plaque score reduction compared with the RMNPI.^40^, ^41^ This effectively contributes to a wider CI, which is evident from the data presented in Tables 2, 3, 4, 5.

### 4.3 Oral hygiene instruction

If optimal results are to be achieved with a toothbrush, professional instruction and reinforcement are needed.^90^, ^91^ Analysis of the included studies revealed that in 22^9^, ^56^, ^57^, ^58^, ^59^, ^60^, ^61^, ^62^, ^64^, ^67^, ^68^, ^72^, ^73^, ^74^, ^75^, ^76^, ^78^, ^79^, ^80^, ^81^, ^82^ studies, only written instructions were provided of which three^74^, ^76^, ^78^ studies only instructed those using the PTB and gave no instructions for the MTB. In five^55^, ^63^, ^65^, ^77^, ^81^ studies, visual and /or verbal instructions were given to their participants. In one^70^ study, no instructions were provided as the purpose was to evaluate what the effect of brushing was in participants that habitually used either an MTB or PTB (for details, see online appendix Table S2). Considering this outcome, a certain level of bias may be introduced when only those using a PTB received instructions. The efficacy as observed may also have been higher for both the MTB and the PTB if more effort had been put in individual professional instructions.

As the included studies were single‐brushing actions, familiarization with the brushes under research is required, especially if individuals habitually using an MTB are participating in the PTB group. In 14^55^, ^57^, ^58^, ^59^, ^63^, ^65^, ^67^, ^72^, ^75^, ^78^, ^79^, ^80^, ^81^ out of the 28 studies, such a familiarization phase was part of the research design. In one study, this phase was not needed as the participants were using the type of brush they used at home. What the impact of the absence of a familiarization phase is on the outcome of the studies was not further analysed but can be considered a limitation. This may have had an impact on the results of brushing with the toothbrush the participants were not familiar with.

### 4.4 Study design

The study design that evaluates a single‐brushing action provides an assessment under ideal conditions in which all participants comply with the use of the device to which they are randomly assigned.^16^ Although the design is clearly restricted to an instant evaluation of one‐time brushing action under controlled circumstances, when data indicate that a specific toothbrush shows a greater potential in reducing plaque scores, it can be supposed that it offers improved plaque control over time. Consequently, it may also have long‐term benefits for gingival health.^16^ The American Dental Association (ADA), in their acceptance programme requirements for an ADA‐seal, requests a minimum study duration of 30‐days to show improved reduction in plaque and gingivitis scores.^92^ Ideally, study should involve a combination of single‐brushing designs and short‐ and long‐term studies that establish robust evidence for a particular toothbrush. The clinical importance of the findings also deserves attention. In this respect, phase IV studies are needed to confirm the long‐term clinical importance of PTB use and for safety surveillance. Studies extending over several years not only provide data related to the prevention of periodontal diseases but also to the prevention of caries.^93^, ^94^

The practical difficulty is that PTB manufacturers frequently change toothbrush design or technology, adjust brush heads and introduce other technological improvements. This may result in a long‐term study presenting results on a specific PTB that is no longer available. Recently, an 11‐year prospective population‐based cohort evaluated the longitudinal effects of PTB on periodontal health, caries and tooth loss in an adult population.^95^ It showed an effect in reducing the progression of PD and CAL in the study participants. Therefore, it seems that PTB usage in the long run helps maintain the number of teeth in the oral cavity and reduces the progression of periodontal disease burden.^95^

### 4.5 Clinical relevance

As with an MA, an SMD as a summary of multiple plaque indices can be calculated with an NMA. Furthermore, a DiffM can be calculated for a specific plaque index. The results of the present NMA show larger differences between the indirect and direct evidence analysis when comparing the outcomes for the SMD with the DiffM. This is presumably due to the variation within and between indices and the subsequent SDs. Hence, it appears legitimate to perform the analysis separately per plaque index score and synthesize the data according to the DiffM. This also allows a direct interpretation of outcomes relative to its original scale, which is crucial for estimating and judging the clinical relevance of the observed difference. This also helps clinicians to interpret the scientific findings in their daily practice. The contribution of indirect data to the overall NMA appears to be more pronounced for the post‐brushing data than the data of the incremental change (see Tables 2, 3, 4, 5). This may be because the incremental change between pre‐ and post‐brushing is also affected by the variation in baseline brushing scores. Although there is no statistical difference at baseline, scores can differ due to details in study design choices such as duration of plaque accumulation and dietary instructions. In the present study, this could not be analysed in detail.

For the analysis of post‐brushing scores concerning the MQ&HPI,^37^, ^38^, ^39^ only a significant difference is found for the direct comparisons between the OR PTB and MTB. When the incremental change in plaque scores is considered, analysis with the RMNPI^40^, ^41^ shows a significant difference irrespective of the inclusion of indirect comparisons (Table 5). The incremental change can also be used for interpreting the clinical relevance. The DiffM of MQ&HPI^37^, ^38^, ^39^ scores range from 0.04 to 0.18 on a scale of 0–5 (Table 5), and for RMNP^40^, ^41^ index, scores range from 0.05 to 0.13 on a scale of 0–1. The latter translates approximately as a 13% difference, a figure that could result in a clinically significant effect on gingival health.^96^

### 4.6 Should everyone use a PTB?

Toothbrushes in general are the most recommended oral care product.^97^ The conclusion that a PTB removes more dental plaque than an MTB raises the question whether people should always use a PTB. As this significantly impacts professional recommendations and public knowledge, such a message should be posted with vigilance. Evidence‐based advice should include details of PTB costs and should not be limited to plaque removal effectiveness but should include the maintenance or improvement of periodontal health.

In 2006, Porter introduced the term value‐based health care (VBH).^98^ This concept is based on a cost‐effectiveness principle and is currently well integrated into the medical field, particularly in Western societies.^98^ Value‐based oral health care (VBOHC) is about improving people's oral health outcomes divided by the costs—that is, ‘patient health outcomes achieved per dollar spent’.^99^, ^100^ Currently, such an analysis for something as basic as a toothbrush has not been performed, and certainly not for PTBs and their different modes of action. For such an analysis, there are several aspects to consider. First, the cost of a PTB is substantially more than an MTB and also comes with a variety of models and prices. Second, it needs to be ascertained how much the expected improvement in plaque removal and subsequent preventive effect of improved gingival health and reduced caries risk will cost. As periodontitis accounts for a considerable proportion of edentulism and masticatory dysfunction, it has a negative impact on general health and results in significant dental care costs.^101^, ^102^ Thus, indirectly, adequate plaque removal reduces the need for treatment and, consequently, toothbrushing presumably reduces oral healthcare costs in both the short and long term. Based on this complex series of considerations and consequent calculations, VBOHC can be used to gain more insights into which oral health outcome can be obtained for a specific person by using a PTB. This insight will answer the question raised earlier of whether the financial expense for a PTB is realistic and beneficial for everyone.

### 4.7 Limitations and recommendations

‐ Only publications written in the English language were included in this SR and NMA. This prerequisite may have introduced a language bias, although the extent and effect of this may be negligible due to the shift towards publications in English in recent decades and the high number of included studies.^29^
‐ Only full‐text publications were considered, and no abstracts from scientific meetings or manufacturers’ data on file were sought. This restriction may have introduced a publication bias. However, internationally published papers have been through the peer review process, which is intended to safeguard content quality.^103^
‐ Blinding participants during clinical trials comparing MTB and PTB is not possible. Participants will see and experience the difference, and this is also true for the different PTB modes of action (ie OR and HFS.)
‐ This SR included publications dated from 1992, and the most recent study was from 2020. The changes in toothbrusg design occurring in the intervening 28 years could possibly affect the outcome. Both MTBs and PTBs have undergone technological improvements over recent decades.^104^ While the original technology of the OR movements or sonic vibrations is essentially unchanged, it has been optimized, and this also applies to brush head design.
‐ The new development of digital software to optimize patients’ oral hygiene performance—such as timers, pressure sensors, apps, and artificial‐intelligence brushing recognition and guidance—is not considered in this review.

## 5 CONCLUSIONS

Within the limitations of the present study design, based on the outcome of single acts of brushing, it can be concluded that for dental plaque removal, there is a high certainty for a small effect of PTB efficiency compared with an MTB. This supports the recommendation to advise using a PTB rather than an MTB. There is moderate certainty for a very small benefit in using an OR mode of action PTB rather than a HFS PTB.

## 6 CLINICAL RELEVANCE

### 6.1 Scientific rationale for the study

Toothbrushing is considered the most efficient way to remove dental plaque and prevent periodontal diseases. At present, no network meta‐analysis (NMA) of the available literature has been performed concerning the efficacy of different powered toothbrush technologies on plaque removal.

### 6.2 Principal findings

The NMA demonstrated that an oscillating‐rotating (OR) or a high‐frequency sonic (HFS) powered toothbrush (PTB) is more effective than a manual toothbrush. When comparing the two PTB technologies, OR ranks higher than HFS.

### 6.3 Practical implications

When recommending a toothbrush to a patient, a PTB is more effective than a manual toothbrush and should be considered the first choice. The clinical relevance of the very small but significant difference in favour of OR over HFS technology needs further appraisal based on long‐term studies.

## CONFLICT OF INTEREST

The authors declare that they have no conflicts of interest.

## AUTHORS CONTRIBUTIONS

All authors approved the final version of this manuscript before submission and agreed to be accountable for all aspects of the work ensuring that questions related to the accuracy or integrity of any part of the work were appropriately addressed and resolved. TMJAT contributed to design, search and selection, analysis and interpretation and drafted the manuscript, and DES contributed to conception and design, search and selection, analysis and interpretation and critically revised the manuscript. GAW contributed to conception and design, analysis and interpretation and critically revised the manuscript.

## Supporting information

Supplementary Material

## Data Availability

The data are derived from public domain resources. The data that support the findings (the included studies) of this study are available from search databases PubMed/Medline and Cochrane‐CENTRAL. These data were derived from resources available in original papers that are published in the public domain.
