# Supplementary material for: The efficacy of powered toothbrushes: A systematic review and network meta‐analysis
Source: Int J Dent Hyg. 2021 Dec 31;20(1):3–17. doi: 10.1111/idh.12563 (PMC9303421; doi:10.1111/idh.12563)
Supplement: Supplementary file 1 — Supplementary Material [file IDH-20-3-s001.docx]

# The efficacy of toothbrushes following a brushing exercise:

# *-A Systematic Review and Network Meta-Analysis-*

#

# Tim M.J.A. Thomassen

# G.A. (Fridus) Van der Weijden

# Dagmar E. Slot

Online Appendices

**Online supporting information index**

**Appendix S1.**

Papers excluded after full text reading with the details for rejection.

**Appendix S2**.

Overview of the included studies and characteristics processed for data extraction

**Appendix S3a.**

Summary Information on studies included in the network meta-analyse (NMA), including interventions, data on Difference of Means, Risk of Bias and Indirectness. *(M)Q&HPI.*

**Appendix S3b**.

Summary Information on studies included in the network meta-analyse (NMA), including interventions, data on Difference of Means, Risk of Bias and Indirectness. *RMNPI*

**Appendix S4**.

Methodological quality and potential risk of bias scores of the individual included studies

**Appendix S5a**.

Forest plot of the overall analysis of the comparisons at pre-brushing.

**Appendix S5b**.

Forest plot comparing OR and HFS with the MTB. Overall. *Pre-Brushing*

**Appendix S6a**.

Forest plot of the sub analysis of the comparisons. (M)Q&HPI. *Pre-brushing*

**Appendix S6b**.

Forest plot of the sub analysis comparing OR and HFS with the MTB. (M)Q#HPI. *Pre-Brushing*

**Appendix S7a**.

Forest plot of the sub analysis of the comparisons. RMNPI. *Pre-brushing*

**Appendix S7b**.

Forest plot of the sub analysis comparing OR and HFS with the MTB. RMNPI. *Pre-Brushing*

**Appendix S8a**.

Forest plot of the overall analysis of the comparisons at post-brushing

**Appendix S8b**.

Treatment Ranking. Overall results presented irrespective of indices used. *Post-Brushing*

**Appendix S8c.**

Forest plot comparing OR and HFS with the MTB. *Post-Brushing*

**Appendix S9a**.

Forest plot of the sub analysis of the comparisons. (M)QHPI. *Post-brushing*

**Appendix S9b.** Treatment Ranking. Sub-analysis on (M)Q&HPI. *Post-Brushing*

**Appendix S9c**.

Forest plot comparing OR and HFS with the MTB. Sub-analysis on (M)Q&HPI. *Post-Brushing*

**Appendix S9d**.

Specific **Ranking table for all studies using (M)Q&HPI- Probability for each treatment to be the best.** *Post-Brushing*

**Appendix S10a**.

Forest plot of the sub analysis of the comparisons. RMNPI. *Post-brushing*

**Appendix S10b**.

Treatment Ranking. Sub-analysis on RMNPI. *Post-Brushing*

**Appendix S10c**.

Forest plot comparing OR and HFS with the MTB. Sub-analysis on RMNPI. *Post-Brushing*

**Appendix S10d**.

Specific **Ranking table for all studies using RMNPI- Probability for each treatment to be the best.** *Post-Brushing*

**Appendix S11a**.

Forest plot of the overall analysis of the comparisons of the reduction in plaque scores, irrespective of the plaque indices used.

**Appendix S11b.**

Treatment Ranking. Overall results presented irrespective of indices used. *Incremental reduction between pre- and post brushing*

**Appendix S11c**.

Forest plot comparing OR and HFS with the MTB. *Incremental reduction between pre- and post brushing*

**Appendix S12a.** Forest plot of the overall analysis of the comparisons of the change in plaque scores on the (M)Q&HPI

**Appendix S12b**.

Treatment Ranking. Sub-analysis on (M)Q&HPI. *Incremental reduction between pre- and post brushing*

**Appendix S12c.**

Forest plot comparing OR and HFS with the MTB. Sub-analysis on (M)Q&HPI. *Incremental reduction between pre- and post brushing*

**Appendix S12d**.

Specific **Ranking table for all studies using (M)Q&HPI- Probability for each treatment to be the best.** *Incremental reduction between pre- and post brushing*

**Appendix S13a.**

Forest plot of the overall analysis of the comparisons of the change in plaque scores on the RMNPI.

**Appendix S13b.**

Treatment Ranking. Sub-analysis on RMNPI. *Incremental reduction between pre- and post brushing*

**Appendix S13c**.

Forest plot comparing OR and HFS with the MTB. Sub-analysis on RMNPI. *Incremental reduction between pre- and post brushing*

**Appendix S13d**.

Specific **Ranking table for all studies using RMNPI- Probability for each treatment to be the best.** *Incremental reduction between pre- and post brushing*

**Appendix S14**.

MetaInsight Network meta-analysis graphs. *Post-Brushing*

**Appendix S15**.

MetaInsight Network meta-analysis graphs. *Incremental reduction between pre- and post brushing*

**Appendix S16a**.

Confidence in Network Meta-Analysis (CINeMA). Confidence Rating. Overall. *Post-Brushing*

**Appendix S16b**.

Confidence in Network Meta-Analysis (CINeMA). Risk of bias contributions. Overall. *Post-Brushing*

**Appendix S16c.**

Confidence in Network Meta-Analysis (CINeMA). Reporting Bias. Overall. *Post-Brushing*

**Appendix S16d**.

Confidence in Network Meta-Analysis (CINeMA). Indirectness contributions. Overall. *Post-Brushing*

**Appendix S16e.**

Confidence in Network Meta-Analysis (CINeMA). Imprecision.Overall. *Post-Brushing*

**Appendix S16f.**

Confidence in Network Meta-Analysis (CINeMA). Heterogeneity.Overall. *Post-Brushing*

**Appendix S16g.**

Confidence in Network Meta-Analysis (CINeMA). Incoherence. Separating indirect from direct evidence. Overall. *Post-Brushing*

**Appendix S17a.**

Confidence in Network Meta-Analysis (CINeMA). Confidence Rating. Sub-analysis (M)Q&HPI. *Post-Brushing*

**Appendix S17b.**

Confidence in Network Meta-Analysis (CINeMA). Risk of bias contributions. Sub-analysis (M)Q&HPI. *Post-Brushing*

**Appendix S17c.**

Confidence in Network Meta-Analysis (CINeMA). Reporting Bias. Sub-analysis (M)Q&HPI. *Post-Brushing*

**Appendix S17d.**

Confidence in Network Meta-Analysis (CINeMA). Indirectness contributions. Sub-analysis (M)Q&HPI. *Post-brushing*

**Appendix S17e**.

Confidence in Network Meta-Analysis (CINeMA). Imprecision. Sub-analysis (M)Q&HPI. *Post-Brushing*

**Appendix S17f.**

Confidence in Network Meta-Analysis (CINeMA). Heterogeneity. Sub-analysis (M)Q&HPI. *Post-Brushing*

**Appendix S17g.**

Confidence in Network Meta-Analysis (CINeMA). Incoherence. Separating indirect from direct evidence. Sub-analysis (M)Q&HPI. *Post-Brushing*

**Appendix S18a.**

Confidence in Network Meta-Analysis (CINeMA). Confidence Rating. Sub-analysis RMNPI. *Post-Brushing*

**Appendix S18b**.

Confidence in Network Meta-Analysis (CINeMA). Risk of bias contributions. Sub-analysis RMNPI. *Post-Brushing*

**Appendix S18c**.

Confidence in Network Meta-Analysis (CINeMA). Reporting Bias. Sub-analysis RMNPI. *Post-Brushing*

**Appendix S18d.**

Confidence in Network Meta-Analysis (CINeMA). Indirectness contributions. Sub-analysis RMNPI. *Post-Brushing*

**Appendix S18e.**

Confidence in Network Meta-Analysis (CINeMA). Imprecision. Sub-analysis RMNPI. *Post-Brushing*

**Appendix S18f**.

Confidence in Network Meta-Analysis (CINeMA). Heterogeneity. Sub-analysis RMNPI. *Post-Brushing*

**Appendix S18g**.

Confidence in Network Meta-Analysis (CINeMA). Incoherence. Separating indirect from direct evidence. Sub-analysis RMNPI. *Post-Brushing*

**Appendix S19a.**

Confidence in Network Meta-Analysis (CINeMA). Confidence Rating. *Incremental reduction between pre- and post brushing*

**Appendix S19b.**

Confidence in Network Meta-Analysis (CINeMA). Risk of bias contributions. The bar chart shows the contributions of each piece of study to the network estimate. *Incremental reduction between pre- and post brushing*

**Appendix S19c.**

Confidence in Network Meta-Analysis (CINeMA). Reporting Bias. *Incremental reduction between pre- and post brushing*

**Appendix S19d.**

Confidence in Network Meta-Analysis (CINeMA). Indirectness contributions. *Incremental reduction between pre- and post brushing*

**Appendix S19e**.

Confidence in Network Meta-Analysis (CINeMA). Imprecision. *Incremental reduction between pre- and post brushing*

**Appendix S19f.**

Confidence in Network Meta-Analysis (CINeMA). Heterogeneity. *Incremental reduction between pre- and post brushing*

**Appendix S19g.**

Confidence in Network Meta-Analysis (CINeMA). Incoherence. Separating indirect from direct evidence. *Incremental reduction between pre- and post brushing*

**Appendix S20a**.

Confidence in Network Meta-Analysis (CINeMA). Confidence Rating. Sub-analysis (M)Q&HPI. *Incremental reduction between pre- and post brushing*

**Appendix 20b.**

Confidence in Network Meta-Analysis (CINeMA). Risk of bias contributions. Sub-analysis (M)Q&HPI. *Incremental reduction between pre- and post brushing*

**Appendix S20c.**

Confidence in Network Meta-Analysis (CINeMA). Reporting Bias. Sub-analysis (M)Q&HPI. *Incremental reduction between pre- and post brushing*

**Appendix S20d.**

Confidence in Network Meta-Analysis (CINeMA). Indirectness contributions. Sub-analysis (M)Q&HPI. *Incremental reduction between pre- and post brushing*

**Appendix S20e.**

Confidence in Network Meta-Analysis (CINeMA). Imprecision. Sub-analysis (M)Q&HPI. *Incremental reduction between pre- and post brushing*

**Appendix S20f.**

Confidence in Network Meta-Analysis (CINeMA). Heterogeneity. Sub-analysis (M)Q&HPI. *Incremental reduction between pre- and post brushing*

**Appendix S20g.**

Confidence in Network Meta-Analysis (CINeMA). Incoherence. Separating indirect from direct evidence. Sub-analysis (M)Q&HPI. *Incremental reduction between pre- and post brushing*

**Appendix S21a**.

Confidence in Network Meta-Analysis (CINeMA). Confidence Rating. Sub-analysis RMNPI. *Incremental reduction between pre- and post brushing*

**Appendix S21b.**

Confidence in Network Meta-Analysis (CINeMA). Risk of bias contributions. Sub-analysis RMNPI. *Incremental reduction between pre- and post brushing*

**Appendix S21c.**

Confidence in Network Meta-Analysis (CINeMA). Reporting Bias. Sub-analysis RMNPI. *Incremental reduction between pre- and post brushing*

**Appendix S21d.**

Confidence in Network Meta-Analysis (CINeMA). Indirectness contributions. Sub-analysis RMNPI. *Incremental reduction between pre- and post brushing*

**Appendix S21e.**

Confidence in Network Meta-Analysis (CINeMA). Imprecision. Sub-analysis RMNPI. *Incremental reduction between pre- and post brushing*

**Appendix S21f.**

Confidence in Network Meta-Analysis (CINeMA). Heterogeneity. Sub-analysis RMNPI. *Incremental reduction between pre- and post brushing*

**Appendix S21g.**

Confidence in Network Meta-Analysis (CINeMA). Incoherence. Separating indirect from direct evidence. Sub-analysis RMNPI. *Incremental reduction between pre- and post brushing*

**Appendix S22.**

References used in the online appendices

**Appendix S1.** Papers excluded after full text reading with the details for rejection.

| **Excluded** | |
| --- | --- |
| Children (8) | Ghassemi et al. 2013; Kallar et al. 2011; Garcia-Godoy et al. 2001; McCracken et al. 2001; Jongenelis et al. 1997; Grossman et al. 1997; Tewari et al. 1992; Erbe et al. 2018 |
| No Single use (44) | García-Carrillo et al. 2016; Klukowska et al. 2014; Nobre et al. 2014; Nightingale et al. 2014; Jain et al. 2013; Sharma et al. 2012; Parizi et al. 2011; Sharma et al. 2010; Pelka et al. 2011; Moritis et al. 2008; Zimmer et al. 2005; Barnes et al. 2003; Lazarescu et al. 2003; Danser et al. 2003; Zimmer et al. 2002; Dentino et al. 2002; Warren et al. 2001; Zimmer et al. 2000; Aass et al. 2000; van der Weijden et al. 1998; Cronin et al. 1998; Yankell et al. 1997; Tritten et al. 1996; Stabholz et al. 1996; Terezhalmy et al. 1995; Stoltze et al. 1994; van der Weijden et al. 1994; Yankell et al. 1994; Emling et al. 1994; Grossman et al. 1994; van der Weijden et al. 1993; Barnes et al. 1993; Hotta et al. 1992; Silverstone et al. 1992; Preber et al. 1991; Mayer et al. 1990; De Beule et al. 1990; Tscharre-Zachhuber et al. 1989; Walsh et al. 1989; Baab et al. 1989; Niemi et al. 1987; Glavind et al. 1986; Walsh et al. 1984; Goldman et al. 1974 |
| No full mouth scores (3) | Oijma et al. 2003; Wiedemann et al. 2001; Niemi et al. 1986 |
| Other devices than OR and HFS (3) | McCracken et al. 2000; Killoy et al. 1989; Khocht et al. 1992 |
| Others (11) | Kemp et al. 2012; Haffajee et al. 2001; Haffajee et al. 2001; Steenackers et al. 2001; Addy et al. 1999; Johnson et al. 1994; Rapley et al. 1994; Engel et al. 1993; Van der Weijden et al. 1993; Manhold et al. 1967; Davidovich et al. 2017 |

**Appendix S2**. Overview of the included studies and characteristics processed for data extraction

| **Authors**  **(year)** | **Study design** | **Plaque index** | **Participants baseline (end),**  **gender,**  **age (mean/range),** | **Mode of action per group/**  ***brands*** | **Instruction method (I),**  **Familiarization phase**  **(F)** | **Conclusions of the original authors** |
| --- | --- | --- | --- | --- | --- | --- |
| Renton-Harper  et al. (2001) | RCT  Cross over | Q&H | 16 (16)  ♀: 10  ♂: 6  Mean age: ?  Age range: 23-41 | OR-PTB *(Braun Oral-B Plaque Remover D5)*  OR-PTB *(Braun Oral-B Plaque Remover D9)*  MTB *(Oral-B 35)* | I:  PTB> written  MTB> none  F: yes, 7 days | The present study showed greater benefits from the use of electric brushes. |
| Heasman et al. (1999) | RCT  Parallel | Q&H | 75 (74)  ♀: ?  ♂: at least 10  Mean age: ?  Age range: 18-25 | OR-PTB  *(Philips Jordan HP 735)*  OR-PTB *(Braun Oral-B D7)*  MTB  *(Oral-B 35 advantage)* | I: visual  F: yes, 6 weeks | There were no significant differences for PI between groups following supervised brushing at 24h. |
| Van der Weijden et al. (1996) | RCT  Cross over | Q&H | 20 (20)  ♀: ?  ♂: ?  Mean age: ?  Age range:? | OR-PTB *(Braun Plak Control)*  MTB *(?)* | I: written  F: no | The PTB is more effective in plaque removal than the MTB. |
| Moritis et al. (2002) | RCT  Cross over | Q&H | 25 (25)  ♀: 17  ♂: 8  Mean age:45.3  (10.9)  Age range: ? | SS-PTB  *(Sonicare Elite)*  MTB  *(Oral-B 35)* | I: written  F: yes, 10 days | The SS-PTB achieved a significantly greater reduction in plaque than the MTB. |
| Sharma et al. (2006)^34^ | RCT  Parallel | RMNPI | 144 (144)  ♀: 96  ♂: 48  Mean age: 37.5 (10.94)  Age range: 19-66 | SS-PTB  (*Sonicare Elite)*  SS-PTB  *(Oral-B Sonic Complete)*  SS-PTB  *(Waterpik Sensonic)*  MTB *(Oral-B 35 Indicator)* | I: none  F: no | Two out of the three SS-PTB compared favorably to the MTB. |
| Pizzo et al. (2010)^35^ | RCT  Cross over | Q&H | 66(66)  ♀: 28  ♂: 38  Mean age: 36.5 (10.1)  Age range: 18-59 | OR-PTB  *(Oral-B PC 8500)*  MTB *(Oral-B Crossaction Vitalizer)*  MTB *(Oral-B Indicator)* | I:  PTB> written  MTB> none  F: no | The OR-PTB demonstrated to be more effective in plaque control than the 2 MTBs. |
| Sharma et al. (2011) | RCT  Cross over | RMNPI | 40 (40)  ♀: 28  ♂: 12  Mean age: 42.3 (11.36)  Age range: 20-69 | OR-PTB  *(Oral-B Professional Care 1000)*  MTB *(Oral-B Indicator)* | I: written  F: yes, 1 week | The OR-PTB with a novel brush head showed statistically significantly superior plaque reduction compared to an MTB. |
| Nathoo et al. (2012) | RCT  Parallel | RMNPI | 82 (76)  ♀: 51  ♂: 25  Mean age: 43  Age range: 18-66 | SS-PTB  *(Colgate Pro Clinical A1500 Triple clean brush head)*  MTB *(Oral-B Indicator)* | I: written  F: no | The SS-PTB provides statistically significant and clinically relevant levels of efficacy in the removal of supragingival dental plaque after a single tooth brushing compared to an MTB. |
| Klukowska et al. (2012) | RCT  Cross over | Q&H | 36 (36)  ♀: 31  ♂: 5  Mean age: 45.6 (8.63)  Age range: 25-60 | OR-PTB (with SS movement)  *(Oral-B Vitality TriZone)*  MTB *(?)* | I: written  F: yes, 2- to 3-days | Comparing the brushes, the SS-PTB provided a significantly superior mean whole mouth plaque reduction relative to the manual brush control. |
| Nathoo et al. (2014) | RCT  Parallel | RMNPI | 120 (120)  ♀:71  ♂: 49  Mean age: 41.4  Age range: 18-67 | SS-PTB*(Colgate Pro Clinical C200 Triple clean brush head)*  SS-PTB*(Colgate Pro Clinical C200 Sensitive clean brush head)*  MTB *(Oral-B indicator)* | I: written  F: no | Both SS-PTB’s provides statistically significant and clinically relevant levels of efficacy in the removal of supragingival dental plaque when compared to a manual flat-trim toothbrush. |
| Rosema et al.  (2014) | CCT  Cross sectional | Q&H | 181 (181)  ♀: 125  ♂: 56  Mean age: 22.7  Age range: 18-35 | OR-PTB  *(?)*  MTB *(?)* | I: none  F: no | The PTB removed significantly more plaque than the MTB. |
| Re et al.  (2015) | RCT  Cross over | Q&H | 40 (40)  ♀: 22  ♂: 18  Mean age: 24 (3.5)  Age range: 18-32 | SS-PTB  *(Philips Sonicare DiamondClean 300)*  MTB *(Butler Gum 409 Compact Soft)* | I: visual  F: no | In subjects without any previous experience of a similar technology, the SS-PTB showed a significantly greater plaque reduction compared to the MTB. |
| Gallob et al. (2015) | RCT  Parallel | RMNPI | 80 (79)  ♀: 58  ♂: 21  Mean age: 49.3  Age range: 19-69 | SS-PTB  *(Colgate ProClinical A1500)*  MTB *(Oral-B Indicator)* | I: written  F: no | Compared to the MTB, the SS-PTB demonstrates statistically and clinically significantly greater levels of plaque removal |
| Kurtz et al. (2016) | RCT  Cross over | Q&H | 95 (87)  ♀: 54  ♂: 41  Mean age: 30.2 (11.87)  Age range: 18-67 | OR-PTB  *(Oral-B Vitality D12)*  MTB *(?)* | I:  PTB> written  MTB> none  F: no | The OR-PTB removed significantly more plaque than the MTB. |
| Kulkarni et al.  (2018) | RCT  Parallel | Q&H | 45  ♀: 30  ♂: 15  Mean age: ?  Age range: 18-25 | OR-PTB  *(Oral-B 2D)*  MTB *(Oral-B 40 advantage)* | I: written  F: no | All individuals within both groups showed reduction in PI. |
| Robinson et al. (1997) | RCT  Parallel | Q&H | 66 (54)  ♀: 32◊  ♂: 22◊  Age ?  Range ? | OR-PTB  *(Braun Oral-B D7)*  HFS-PTB:  *(Side to side Optiva Corporation)* | I: verbal and video  F: yes, 2-3 weeks | Both the OR-PTB and the HFS-PTB improved the mean overall plaque scores. |
| Yankell et al.(1997) | RCT  Parallel | Q&H | 128 (115◊)  ♀: ?  ♂: ?  Age ?  Range 18-50 | OR-PTB:  *(Braun Oral-B)*  HFS-PTB:  *(Side to side Optiva)* | I: written  F: no | The OR-PTB and the HFS-PTB removed statistically significant amounts of plaque after brushing. |
| Sharma et al. (1998) | RCT  Parallel | RMNPI | 44 (44)  ♀: 26  ♂: 18  36.6 (9.6)  22-60 | OR-PTB:  *(Braun Oral-B 3D)*  HFS-PTB:  *(Side to side)* | I: visual, written  F: yes, 4 weeks | Significantly greater plaque reduction was achieved with a new electric toothbrush with a three-dimensional action (OR-PTB), when compared with the HFS-PTB. The OR-PTB was also greatly preferred by the participants, a fact which is likely to increase long-term compliance |
| Sharma et al. (2005) | RCT  Cross-over | RMNPI | 79 (79)  ♀: 51  ♂: 28  36.3 (?)  18-53 | OR-PTB:  *(Oral-B PC7000)*  OR-PTB:  *(Oral-B 3D)*  HFS-PTB:  *(Side to side)* | I: written  F: no | Both OR-PTBs were more effective in plaque removal than the HFS-PTB. |
| Strate et al. (2005) | RCT  Cross-over | RMNPI | 61 (61)  ♀: 38  ♂: 23  37.8 (?)  19-64 | OR-PTB:  *(Oral-B Professional )*  HFS-PTB:  *(Side to side Elite, Philips)* | I: written  F: no | The OR-PTB is more effective at plaque removal than a HFS-PTB. |
| Biesbrock et al. (2007-1) | RCT  Cross-over | RMNPI | 50 (50)  ♀: 36  ♂: 14  41.1 (?)  18-64 | OR-PTB:  *(Oral-B Triumph)*  HFS-PTB:  (*Side to side Elite 7300)* | I: written  F: yes, 7 days | The OR-PTB was significantly more effective in single-use plaque removal than the HFS-PTB with a standard brush head. |
| Biesbrock et al. (2007-2) | RCT  Cross-over | RMNPI | 49 (48)  Participants in general good health  ♀: 34  ♂: 14  43.5 (?)  20-67 | OR-PTB:  *(Oral-B Triumph)*  HFS-PTB:  *(Side to side Elite 7300)* | I: written  F: yes, 10 days | The OR-PTB was significantly more effective in single-use plaque removal than the HFS-PTB with a compact brush head. |
| Biesbrock et al. (2008) | RCT  Cross-over | RMNPI | 48 (45)  Participants in good general health  ♀: 37  ♂: 8  41.2 (?)  27-55 | OR-PTB:  *(Oral-B Triumph with)*  HFS-PTB:  *(Side to side FlexCare)* | I: written  F: yes, 5-6 days per brush | The OR-PTB was significantly more effective in plaque removal after single brushing than the HFS-PTB. |
| Putt et al. (2008) | RCT  Cross-over | Q&H | 94 (93)  Population ?  ♀: 64  ♂: 30  38.1 (11.6)  18-60 | OR-PTB:  *(Oral-B Triumph*  *Professional Care 9000)*  HFS-PTB:  *(Philips Side to side Flexcare)* | I: written  F: yes, 7 days | Both the HFS-PTB and the OR-PTB were safe and effective. HFS-PTB reduced plaque scores significantly better than the OR- PTB |
| Williams et al. (2008) | RCT  Cross-over | Q&H | 48 (47)  Healthy adults  ♀: 41  ♂: 7  43.5 (12.1)  20-66 | OR-PTB:  *(Oral-B Triumph*  *Professional Care 9000)*  HFS-PTB:  *(Philips Side to side Flexcare)* | I: written  F: yes, 10-14 days | The OR-PTB delivered superior plaque removal by reducing plaque scores more than the HFS-PTB. |
| Goyal et al. (2009) | RCT  Parallel | RMNPI | 175 (171)  Participants in good general health  ♀: 120  ♂: 55  41 (10.77)  18-68 | OR-PTB:  *(Oral-B Triumph Floss-Action brush head)*  HFS-PTB:  *(Side to side FlexCare with ProResults brush head)* | I: written  F: no | The OR-PTB was significantly more efficacious than the HFS-PTB in reducing plaque levels. |
| Williams et al. (2009) | RCT  Parallel | Q&H | 179 (171)  ♀: 123  ♂: 56  42.3 (12.2)  19-67 | OR-PTB:  *(Oral-B Triumph with FlossAction brush head.)*  HFS-PTB:  *(Side to side FlexCare with the ProResults brush head)* | I: written, verbal  F: no | The OR-PTB was significantly more effective than the HF-STB, both for whole mouth plaque scores and for gingival margin and interproximal scores. |
| Ayad et al. (2012) | RCT  Parallel | RMNPI | 184 (184)  Participants in general good health  ♀: 138◊  ♂: 46◊  45◊ (?)  18-69◊ | OR-PTB:  *(Oral-B Smart Series 5000 with Flossaction brush head)*  HFS-PTB:  *(Side to side Flexcare Contoured brush head)*  HFS-PTB:  *(Colgate ProClinical A1500)* | I: written  F: yes, 4 weeks | The HFS-PTB Colgate ProClinical A1500Triple Clean Brush head provided statistically significantly greater reductions compared to the other two PTBs in whole mouth plaque index scores after a single tooth brushing. |
| Adam et al (2020) | RCT  Cross-over | Q&H | 27 (27)  Participants in general good health  ♀: 14  ♂: 13  42.0 (10.46)  24-62 | OR-PTB  *(Oral B iO/ Ultimate Clean)*  MTB  *(ADA)* | I: visual  F: yes, 3-4 days | OR- toothbrush with microvibrations produced significantly greater plaque reduction compared to the manual toothbrush. |

RCT: Randomized controlled trial

OR-PTB: Oscillating-rotating power toothbrush

HFS-PTB: High Frequency sonic power toothbrush

OP: Oral prophylaxis

DF: Dentifrice

◊: Calculated by the authors of this review based on the presented data in the selected paper

NR: Not reported

?: Unknown

**Appendix S3a**. Summary Information on studies included in the network meta-analyse (NMA), including interventions, data on Difference of Means, Risk of Bias and Indirectness. ***(M)Q&HPI.***

*Risk of Bias: (1) Low,(2) Moderate, (3) High****.*** *Indirectness: (L) Low, (M) Moderate, (H) High*

| Author | Comparison | N | Brushing | | | | | | | |
| --- | --- | --- | --- | --- | --- | --- | --- | --- | --- | --- |
|  |  |  | pre |  | Post |  | Diff |  |  |  |
|  |  |  | Mean | SD | Mean | SD | Mean | SD | Risk of Bias | Indirectness |
| Adam et al 2020^1^ | OR | 27 | 3.987 | 0.35 | 1.702 | 0.49 | -2.250 | 0.49 | 1 | L |
|  | MTB | 27 | 3.924 | 0.35 | 2.631 | 0.49 | -1.321 | 0.49 | 1 | L |
| Heasman et al 1999^2^ | OR | 25 | 2,03 | 0,55 | 0,82 | 0,54 | ? | ? | 1 | L |
|  | MTB | 12 | 2,27 | 0,54 | 1,05 | 0,51 | ? | ? | 1 | L |
| Klukowska et al 2012^3^ | OR | 36 | 2,146 | 0,3551 | 1,104 | 0,4017 | -1.042 | 0.2174 | 1 | L |
|  | MTB | 36 | 2,169 | 0,323 | 1,196 | 0,408 | -0.973 | 0.2516 | 1 | L |
| Kulkarni et al 2017^4^ | OR | 22 | 1,77 | 0,34 | 0,81 | 0,39 | -0.957 | 0.4233 | 3 | L |
|  | MTB | 23 | 1,56 | 0,44 | 0,61 | 0,34 | -0.946 | 0.4066 | 3 | L |
|  | OR | 22 | 1,43 | 0,56 | 0,54 | 0,36 | -0.885 | 0.3938 | 3 | L |
|  | MTB | 23 | 1,53 | 0,54 | 0,58 | 0,42 | -0.957 | 0.4088 | 3 | L |
|  | OR | 22 | 0,98 | 0,45 | 0,25 | 0,19 | -0.725 | 0.3701 | 3 | L |
|  | MTB | 23 | 0,88 | 0,37 | 0,29 | 1,24 | -0.592 | 0.3236 | 3 | L |
|  | OR | 22 | 0,7 | 0,44 | 0,13 | 0,13 | -0.566 | 0.4223 | 3 | L |
|  | MTB | 23 | 0,71 | 0,31 | 0,17 | 0,19 | -0.537 | 0.2619 | 3 | L |
| Kurtz et al 2016^5^ | OR | 90 | 2,331 | 0,4842 | 1,604 | 0,4353 | -0.726 | 0.2996 | 2 | L |
|  | MTB | 90 | 2,307 | 0,5212 | 1,741 | 0,4293 | -0.567 | 0.2429 | 2 | L |
|  | OR | 90 | 2,275 | 0,4306 | 1,527 | 0,377 | -0.748 | 0.2568 | 2 | L |
|  | MTB | 90 | 2,272 | 0,4501 | 1,677 | 0,3891 | -0.594 | 0.2158 | 2 | L |
|  | OR | 60 | 2,325 | 0,3954 | 1,508 | 0,4205 | -0.817 | 0.2705 | 2 | L |
|  | MTB | 60 | 2,31 | 0,4378 | 1,597 | 0,3662 | -0.713 | 0.249 | 2 | L |
|  | OR | 32 | 2,203 | 0,5313 | 1,555 | 0,3931 | -0.649 | 0.247 | 2 | L |
|  | MTB | 32 | 2,167 | 0,4892 | 1,695 | 0,3953 | -0.473 | 0.2117 | 2 | L |
| Moritis et al 2002^6^ | HFS | 25 | 2,7 | 0,4 | 1,73 | 0,47 | ? | ? | 1 | L |
|  | MTB | 25 | 2,55 | 0,44 | 1,89 | 0,47 | ? | ? | 1 | L |
| Pizzo et al 2010^7^ | OR | 33 | 3,4 | 0,67 | 2,43 | 0,78 | -0.97 | 0.43 | 2 | L |
|  | MTB | 66 | 3,42 | 0,69 | 2,65 | 0,86 | -0.78 | 0.38 | 2 | L |
|  | OR | 33 | 3,4 | 0,67 | 2,43 | 0,78 | -0.97 | 0.43 | 2 | L |
|  | MTB | 66 | 3,36 | 2,86 | 2,86 | 0,9 | -0.49 | 0.33 | 2 | L |
| Re et al 2015^8^ | HFS | 40 | 1,85 | 0,51 | 0,66 | 0,47 | -1.19 | 0.37 | 1 | L |
|  | MTB | 40 | 1,83 | 0,65 | 0,66 | 0,48 | -1.05 | 0.22 | 1 | L |
| Renton-Harper et al. 2001^9^ | OR | 16 | 3.137 | 0.147 | 2.481 | 0.349 | -0.656 | ? | 2 | L |
|  | MTB | 8 | 3.179 | 0.208 | 2.402 | 0.414 | -0.777 | ? | 2 | L |
|  | OR | 16 | 3.205 | 0.24 | 2.464 | 0.368 | -0.741 | ? | 2 | L |
|  | MTB | 8 | 3.179 | 0.208 | 2.402 | 0.414 | -0.777 | ? | 2 | L |
|  | OR | 16 | 3.137 | 0.147 | 2.095 | 0.433 | -1.042 | ? | 2 | L |
|  | MTB | 8 | 3.179 | 0.208 | 2.02 | 0.417 | -1.159 | ? | 2 | L |
|  | OR | 16 | 3.205 | 0.24 | 2.132 | 0.467 | -1.073 | ? | 2 | L |
|  | MTB | 8 | 3.179 | 0.208 | 2.02 | 0.417 | -1.159 | ? | 2 | L |
|  | OR | 16 | 3.137 | 0.147 | 1.736 | 0.474 | -1.401 | ? | 2 | L |
|  | MTB | 8 | 3.179 | 0.208 | 1.669 | 0.358 | -1.51 | ? | 2 | L |
|  | OR | 16 | 3.205 | 0.24 | 1.775 | 0.478 | -1.430 | ? | 2 | L |
|  | MTB | 8 | 3.179 | 0.208 | 1.669 | 0.358 | -1.51 | ? | 2 | L |
| Robinson et al1997^10^ | HFS | 29 | 1.99 | 0.44 | 1.78 | 0.39 | ? | ? | 1 | M |
|  | OR | 25 | 1.84 | 0.43 | 1.69 | 0.38 | ? | ? | 1 | M |
|  | HFS | 29 | 1.82 | 0.51 | 1.47 | 0.35 | ? | ? | 1 | M |
|  | OR | 25 | 1.72 | 0.42 | 1.43 | 0.36 | ? | ? | 1 | M |
|  | HFS | 29 | 1.69 | 0.53 | 1.33 | 0.44 | ? | ? | 1 | M |
|  | OR | 25 | 1.65 | 0.43 | 1.37 | 0.39 | ? | ? | 1 | M |
| Rosema et al 2014^11^ | OR | 91 | 2,8 | 0,45 | 1,56 | 0,471 | -1.24 | 0.301 | 1 | L |
|  | MTB | 90 | 2,5 | 0,366 | 1,51 | 0,349 | -0.594 | 0.2158 | 1 | L |
| Yankell et al.1997^12^ | HFS | 31 | 2.69 | 0.46 | 2.22 | 0.59 | ? | ? | 1 | M |
|  | OR | 28 | 2.66 | 0.45 | 2.23 | 0.60 | ? | ? | 1 | M |
|  | HFS | 31 | 2.83 | 0.45 | 2.34 | 0.55 | ? | ? | 1 | M |
|  | OR | 28 | 2.66 | 0.39 | 2.23 | 0.55 | ? | ? | 1 | M |
|  | HFS | 31 | 2.22 | 0.58 | 2.33 | 0.65 | ? | ? | 1 | M |
|  | OR | 28 | 2.66 | 0.39 | 2.72 | 0.44 | ? | ? | 1 | M |
| Van der Wijden et al. 1996^13^ | OR | 20 | 2.1 | 0.3 | 0.8 | 0.3 | -1.3 | ? | 1 | L |
|  | MTB | 20 | 2.2 | 0.3 | 0.9 | 0.3 | -1.3 | ? | 1 | L |
| Williams et al. 2008^14^ | HFS | 47 | 2.87 | 0.39 | ? | ? | -0.95 | 0.40 | 1 | M |
|  | OR | 48 | 2.85 | 0.38 | ? | ? | -1.06 | 0.39 | 1 | M |
| Williams et al. 2009^15^ | HFS | 89 | 3.12 | 0.42 | 2.32 | 0.47 | -0.8 | 0.33 | 1 | M |
|  | OR | 90 | 3.18 | 0.49 | 2.31 | 0.53 | -0.87 | 0.35 | 1 | M |
|  | HFS | 85 | 2.76 | 0.44 | 1.97 | 0.51 | -0.79 | 0.31 | 1 | M |
|  | OR | 87 | 2.75 | 0.48 | 1.97 | 0.52 | -0.79 | 0.32 | 1 | M |
|  | HFS | 85 | 2.81 | 0.50 | 2.07 | 0.60 | -0.75 | 0.36 | 1 | M |
|  | OR | 86 | 2.78 | 0.53 | 2.06 | 0.53 | -0.73 | 0.33 | 1 | M |
|  | HFS | 47 | 2.87 | 0.39 | ? | ? | -0.95 | 0.40 | 1 | M |
|  | OR | 48 | 2.85 | 0.38 | ? | ? | -1.06 | 0.39 | 1 | M |

**Appendix S3b**. Summary Information on studies included in the network meta-analyse (NMA), including interventions, data on Difference of Means, Risk of Bias and Indirectness. ***RMNPI***

*Risk of Bias: (1) Low,(2) Moderate, (3) High****.*** *Indirectness: (L) Low, (M) Moderate, (H) High*

| Author | Comparison | N | Brushing | | | | | | | |
| --- | --- | --- | --- | --- | --- | --- | --- | --- | --- | --- |
|  |  |  | pre |  | Post |  | Diff |  |  |  |
|  |  |  | Mean | SD | Mean | SD | Mean | SD | Risk of Bias | Indirectness |
| Ayad et al 2012^16^ | HFS | 62 | 0.67 | 0.04 | 0.36 | 0.05 | -0.31 | 0.06 | 1 | M |
|  | OR | 60 | 0,67 | 0.05 | 0.35 | 0.06 | -0.32 | 0.06 | 1 | M |
|  | HFS | 62 | 0.68 | 0.05 | 0.29 | 0.07 | -0.39 | 0.08 | 1 | M |
|  | OR | 60 | 0,67 | 0.05 | 0.35 | 0.06 | -0.32 | 0.06 | 1 | M |
| Biesbrock et al 2007^17^ | HFS | 50 | 0.63 | 0.03 | ? | ? | -0.44 | 0.06 | 1 | M |
|  | OR | 50 | 0.62 | 0.03 | ? | ? | -0.54 | 0.03 | 1 | M |
|  | HFS | 48 | 0.62 | 0.03 | ? | ? | -0.45 | 0.03 | 1 | M |
|  | OR | 48 | 0.62 | 0.03 | ? | ? | -0.5 | 0.03 | 1 | M |
| Biesbrock et al 2008^18^ | HFS | 45 | 0.62 | 0.03 | ? | ? | -0.41 | 0.05 | 1 | M |
|  | OR | 45 | 0.62 | 0.03 | ? | ? | -0.5 | 0.05 | 1 | M |
| Gallob et al 2015^19^ | HFS | 39 | 0.66 | 0.04 | 0.27 | 0.07 | -0.39 | 0.06 | 1 | L |
|  | MTB | 40 | 0.65 | 0.04 | 0.41 | 0.09 | -0.24 | 0.07 | 1 | L |
| Goyal et al 2009^20^ | HFS | 87 | 0.59 | 0.04 | 0.16 | 0.06 | -0.43 | 0.07 | 1 | M |
|  | OR | 84 | 0.60 | 0.05 | 0.11 | 0.04 | -0.51 | 0.05 | 1 | M |
|  | HFS | 88 | 0.60 | 0.03 | 0.17 | 0.06 | -0.43 | 0.05 | 1 | M |
|  | OR | 85 | 0.61 | 0.05 | 0.12 | 0.05 | -0.49 | 0.05 | 1 | M |
|  | HFS | 88 | 0.61 | 0.04 | 0.18 | 0.07 | -0.43 | 0.05 | 1 | M |
|  | OR | 87 | 0.62 | 0.04 | 0.11 | 0.05 | -0.49 | 0.05 | 1 | M |
| Nathoo et al 2012^21^ | HFS | 40 | 0.75 | 0.09 | 0.35 | 0.08 | -0.4 | 0.08 | 1 | L |
|  | MTB | 36 | 0.74 | 0.09 | 0.49 | 0.08 | -0.25 | 0.05 | 1 | L |
| Nathoo et al 2014^22^ | HFS | 40 | 0.71 | 0.1 | 0.3 | 0.1 | -0.41 | 0.07 | 1 | L |
|  | MTB | 20 | 0.72 | 0.14 | 0.35 | 0.08 | -0.27 | 0.15 | 1 | L |
|  | HFS | 40 | 0.75 | 0.11 | 0.32 | 0.1 | -0.43 | 0.09 | 1 | L |
|  | MTB | 20 | 0.75 | 0.14 | 0.45 | 0.13 | -0.27 | 0.15 | 1 | L |
| Sharma et al 1998^23^ | HFS | 44 | 0.68 | 0.05 | 0.34 | 0.08 | -0.34 | 0.08 | 1 | M |
|  | OR | 44 | 0.67 | 0.04 | 0.22 | 0.08 | -0.45 | 0.08 | 1 | M |
| Sharma et al 2005^24^ | HFS | 61 | 0.61 | 0.03 | 0.31 | 0.09 | -0.33 | 0.07 | 1 | M |
|  | OR | 61 | 0.61 | 0.03 | 0.26 | 0.08 | -0.38 | 0.06 | 1 | M |
|  | HFS | 61 | 0.61 | 0.03 | 0.31 | 0.09 | -0.33 | 0.07 | 1 | M |
|  | OR | 61 | 0.63 | 0.03 | 0.26 | 0.07 | -0.38 | 0.06 | 1 | M |
| Sharma et al 2006^25^ | HFS | 35 | 0.66 | 0.04 | 0.16 | 0.09 | -0.5 | 0.08 | 1 | L |
|  | MTB | 12 | 0.65 | 0.03 | 0.14 | 0.07 | -0.51 | 0.06 | 1 | L |
|  | HFS | 36 | 0.66 | 0.04 | 0.11 | 0.06 | -0.54 | 0.05 | 1 | L |
|  | MTB | 12 | 0.65 | 0.03 | 0.14 | 0.07 | -0.51 | 0.06 | 1 | L |
|  | HFS | 36 | 0.66 | 0.04 | 0.07 | 0.05 | -0.59 | 0.05 | 1 | L |
|  | MTB | 12 | 0.65 | 0.03 | 0.14 | 0.07 | -0.51 | 0.06 | 1 | L |
| Sharma et al 2011^26^ | OR | 40 | 0.632 | 0.0223 | 0.076 | 0.026 | -0.554 | 0.034 | 1 | L |
|  | MTB | 40 | 0.625 | 0.0264 | 0.195 | 0.0557 | -0.43 | 0.034 | 1 | L |
| Strate et al 2005^27^ | HFS | 61 | 0.63 | 0.03 | 0.25 | 0.10 | -0.38 | 0.08 | 1 | M |
|  | OR | 61 | 0.63 | 0.03 | 0.08 | 0.05 | -0.55 | 0.05 | 1 | M |
| Terézhalmy et al. 2005^28^ | OR | 108 | 0.374 | 0.101 | ? | ? | -0.27 | 0.07 | 1 | L |
|  | MTB | 216 | 0.376 | 0.093 | ? | ? | -0.211 | 0.07 | 1 | L |
|  | OR | 108 | 0.374 | 0.101 | ? | ? | -0.27 | 0.07 | 1 | L |
|  | MTB | 216 | 0.373 | 0.087 | ? | ? | -0.19 | 0.07 | 1 | L |

**Appendix S4**. Methodological quality and potential risk of bias scores of the individual included studies

| Study  Quality criteria | | Vd Weijden et al. 1996 | Robinson et al. 1997 | Yankel & Emling 1997 | Sharma et al. 1998 | Sharma et al. 2005 | Strate et al. 2005 | Biesbrock et al. 2007 | Biesbrock et al. 2008 | William et al. 2008 | Goyal et al. 2009 | William et al. 2009 | Ayad et al. 2012 |
| --- | --- | --- | --- | --- | --- | --- | --- | --- | --- | --- | --- | --- | --- |
|  | Study design | PA | PA | PA | PA | CO | CO | CO | CO | CO | PA | PA | PA |
| **Internal validity** | Random allocation* | + | + | + | + | + | + | + | + | + | + | + | + |
|  | Allocation concealment | ? | ? | ? | ? | ? | ? | ? | ? | ? | ? | ? | ? |
|  | Blinded to product* | NA | NA | NA | NA | NA | NA | NA | NA | NA | NA | NA | NA |
|  | Blinded to examiner* | + | + | + | + | + | + | + | + | + | + | + | + |
|  | Blinding during statistical analysis | ? | ? | ? | ? | ? | ? | ? | ? | ? | ? | ? | ? |
|  | Balanced experimental groups* | + | + | + | + | + | + | + | + | + | + | + | + |
|  | Reported loss to follow up* | - | + | + | + | + | + | + | + | + | + | + | + |
|  | # (%) of drop-outs | ? | 12  (18,2%◊) | 13◊  (10,2%◊) | 0  (0%◊) | 0  (0%◊) | 0  (0%◊) | 0  (0%◊) | 3  (6,3%◊) | 1  (2,1%◊) | 4  (2,3%◊) | 8  (4,5%◊) | 0  (0%◊) |
|  | Treatment identical, except for intervention* | + | + | + | + | + | + | + | + | + | + | + | + |
| **Externl validity** | Representative population group | + | + | + | ? | + | + | ? | ? | ? | ? | + | + |
|  | Eligibility criteria defined* | + | + | + | + | + | + | + | + | + | + | + | + |

| **Statistical validity** | Sample size calculation and power | - | - | - | - | + | + | - | - | - | + | + | - |
| --- | --- | --- | --- | --- | --- | --- | --- | --- | --- | --- | --- | --- | --- |
|  | Point estimates presented for the primary outcome | + | + | + | + | + | + | + | + | + | + | + | + |
|  | Measures of variability presented for the primary outcome | + | + | + | + | + | + | + | + | + | + | + | + |
|  | Unit of analysis | subject | subject | group | subject | subject | subject | subject | subject | subject | subject | subject | ? |
|  | Include an per protocol analysis | - | + | ? | ? | ? | ? | ? | ? | ? | ? | ? | ? |
|  | Include an intention- to-treat analysis | ? | ? | ? | ? | ? | ? | ? | ? | ? | ? | ? | ? |
|  | Correction for multiple comparisons | NA | NA | NA | NA | NA | NA | NA | NA | NA | NA | NA | NA |
| **Clinical aspects** | Validated measurement | + | + | + | + | + | + | + | + | + | + | + | + |
|  | Calibration examiner | - | + | + | + | + | - | ? | - | + | + | + | + |
|  | Reproducibility data shown | - | - | + | - | - | - | - | - | - | - | - | - |
| **Authors estimated risk of bias** | | **low** | **low** | **low** | **low** | **low** | **low** | **low** | **low** | **low** | **low** | **low** | **low** |

| Study  Quality criteria | | Van der Weijden et al 1996 | Heasman et al. 1999 | Renton-Harper et all. 2001 | Moritis et al. 2002 | Sharma et al. 2006 | Pizzo et al. 2010 | Sharma et al. 2011 | Nathoo et al. 2012 | Klukowska et al. 2012 | Nathoo et al. 2014 | Rosema et al. 2014 | Gallob et al. 2015 | Re et al. 2015 | Kurtz et al. 2016 | Kulkarni et al/. 2018 | Adam et al 2020 |
| --- | --- | --- | --- | --- | --- | --- | --- | --- | --- | --- | --- | --- | --- | --- | --- | --- | --- |
|  | Study design | cross-over | parallel | cross-over | cross-over | parallel | cross-over | cross-over | parallel | cross-over | parallel | cross  sectional | parallel | cross-over | cross-over | parallel | Cross-over |
| **internal validity** | Random allocation * | + | + | + | + | + | + | + | + | + | + | NA | + | + | + | ? | + |
|  | Allocation concealment | ? | ? | ? | ? | ? | + | + | ? | + | ? | - | + | ? | + | ? | ? |
|  | Blinded to  product * | NA | NA | NA | NA | NA | NA | NA | NA | NA | NA | NA | NA | NA | NA | NA | NA |
|  | Blinded to examiner * | + | + | + | + | + | + | + | + | + | + | + | + | + | + | ? | + |
|  | Blinding during statistical analysis | ? | ? | ? | ? | ? | ? | ? | ? | ? | ? | ? | ? | ? | ? | ? | ? |
|  | Balanced experimental groups * | + | + | + | + | + | + | + | + | + | + | + | + | + | + | + | + |
|  | Reported loss to follow up* | + | + | + | + | + | + | + | + | + | + | + | + | + | + | + | + |
|  | # (%) of drop-outs | 0 | 1 (1%)◊ | 0 | 0 | 0 | 0 | 0 | 6 (7%)◊ | 0 | 0 | 0 | 1 (1%)◊ | 0 | 8 (8%)◊ | 0 | 0 |
|  | Treatment identical, except for intervention * | + | + | - | + | + | - | + | + | + | + | + | + | + | - | + | + |
| **External validity** | Representative population group | + | + | + | + | + | + | + | + | + | + | + | + | + | + | + | + |
|  | Eligibility criteria defined * | + | + | + | + | + | + | + | + | + | + | + | + | + | + | + | + |
| **Statistical validity** | Sample size calculation and power | ? | + | ? | ? | ? | + | + | ? | + | ? | - | + | ? | + | + | + |
|  | ADA sample size n≥30^⌂⌂^ | - | - | - | - | + | + | + | + | + | + | + | + | + | + | - | - |
|  | Point estimates presented for the primary outcome | + | + | + | + | + | + | + | + | + | + | + | + | + | + | + | + |
|  | Measures of variability presented for the primary outcome | + | + | + | + | + | + | + | + | + | + | + | + | + | + | + | + |
|  | Unit of analysis | full mouth | full mouth | full mouth | full mouth | full mouth | full mouth | full mouth | full mouth | full mouth | full mouth | full mouth | full mouth | full mouth | full mouth | full mouth | Full mouth |
|  | Include an per protocol analysis | ? | ? | ? | ? | ? | ? | ? | ? | ? | ? | ? | ? | ? | ? | ? | ? |
|  | Include an intention- to-treat analysis | + | + | + | + | + | + | + | + | + | + | + | + | + | + | + | + |
|  | Correction for multiple comparisons | ? | ? | ? | ? | ? | ? | ? | ? | ? | ? | ? | ? | ? | ? | ? | ? |
|  | Validated measurement | + | + | + | + | + | + | + | + | + | + | + | + | + | + | + | + |
|  | Calibration examiner | ? | + | ? | + | + | + | ? | + | + | + | + | + | - | - | + | + |
|  | Reproducibility data shown | - | - | - | - | - | - | - | - | - | - | - | - | - | - | - | - |
| **Authors estimated risk of bias** | | **Low** | **Low** | **moderate** | **Low** | **Low** | **moderate** | **Low** | **Low** | **Low** | **Low** | **Low** | **Low** | **Low** | **moderate** | **High** | **Low** |

**Appendix S5a**. Forest plot of the overall analysis of the comparisons at pre-brushing.


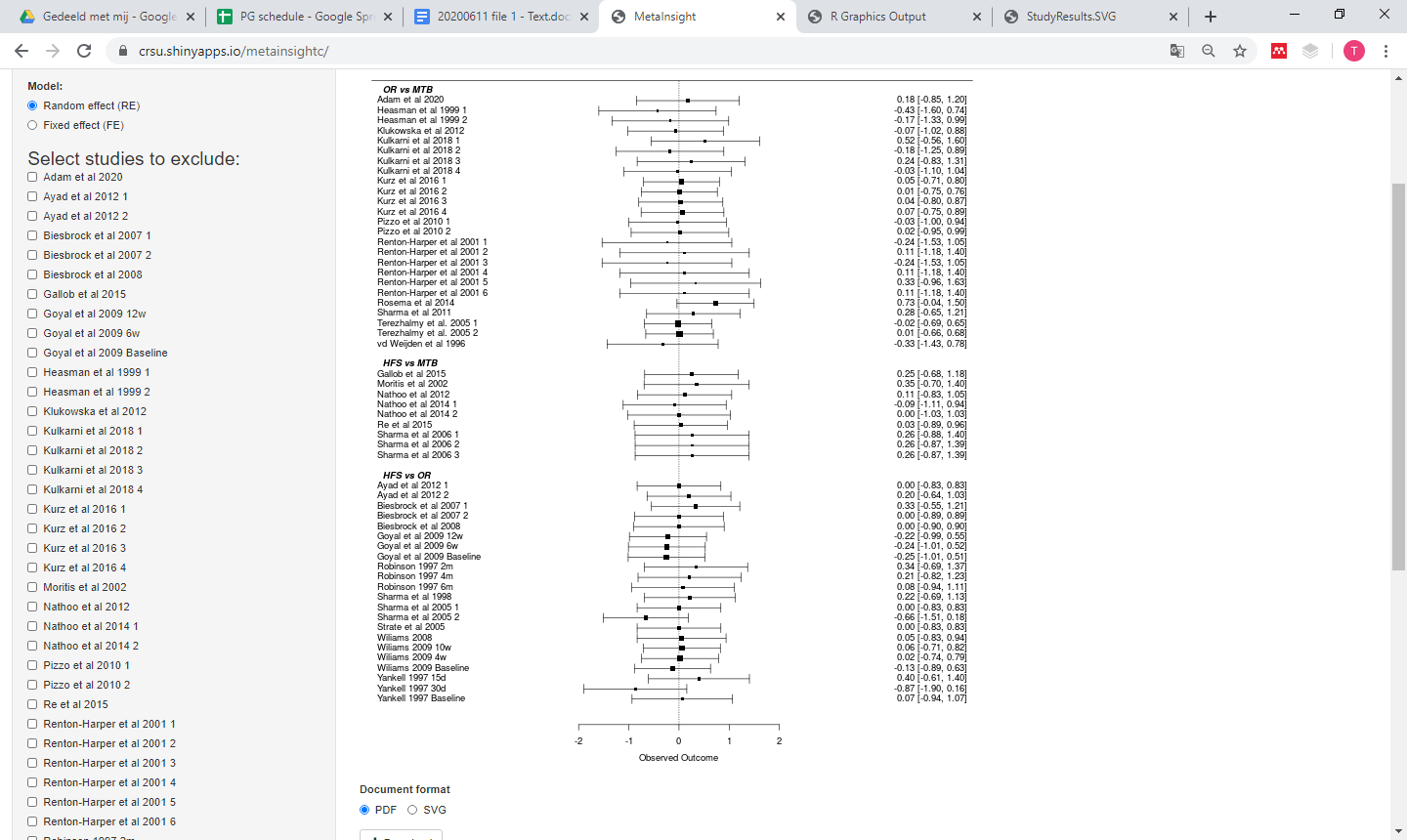


**Appendix S5b**. Forest plot comparing OR and HFS with the MTB. Overall. *Pre-Brushing*


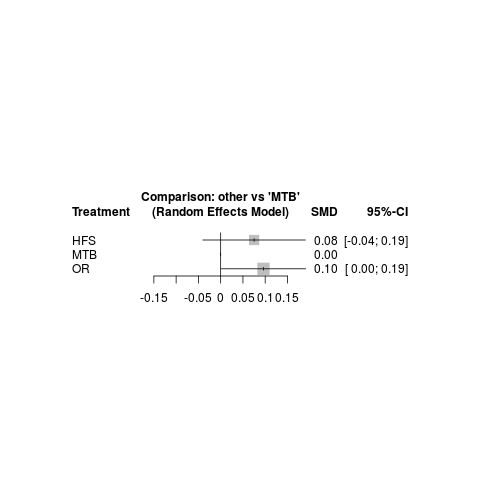


**Appendix S6a**. Forest plot of the sub analysis of the comparisons. (M)Q&HPI. *Pre-brushing*


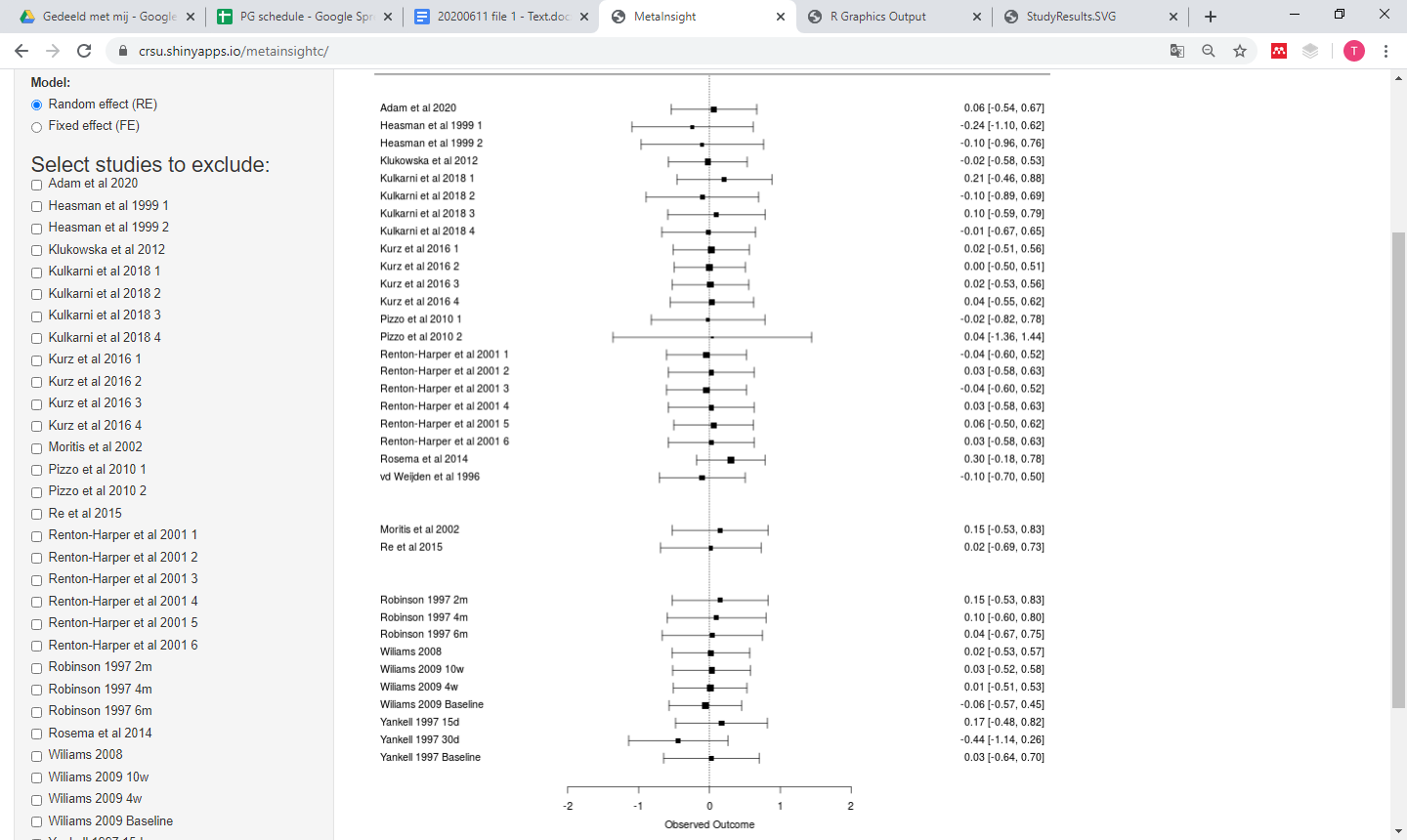


**Appendix S6b**. Forest plot of the sub analysis comparing OR and HFS with the MTB. (M)Q#HPI. *Pre-Brushing*

*
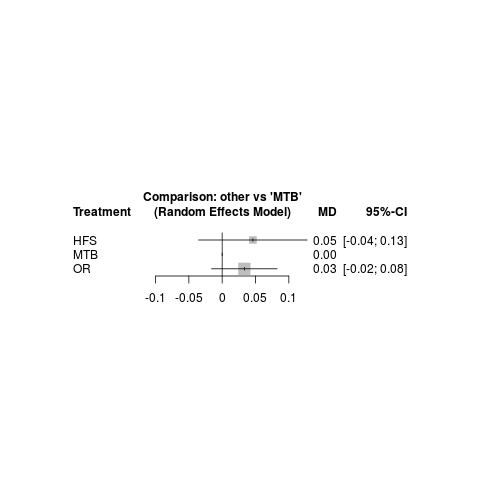
*

**Appendix S7a**. Forest plot of the sub analysis of the comparisons. RMNPI. *Pre-brushing*


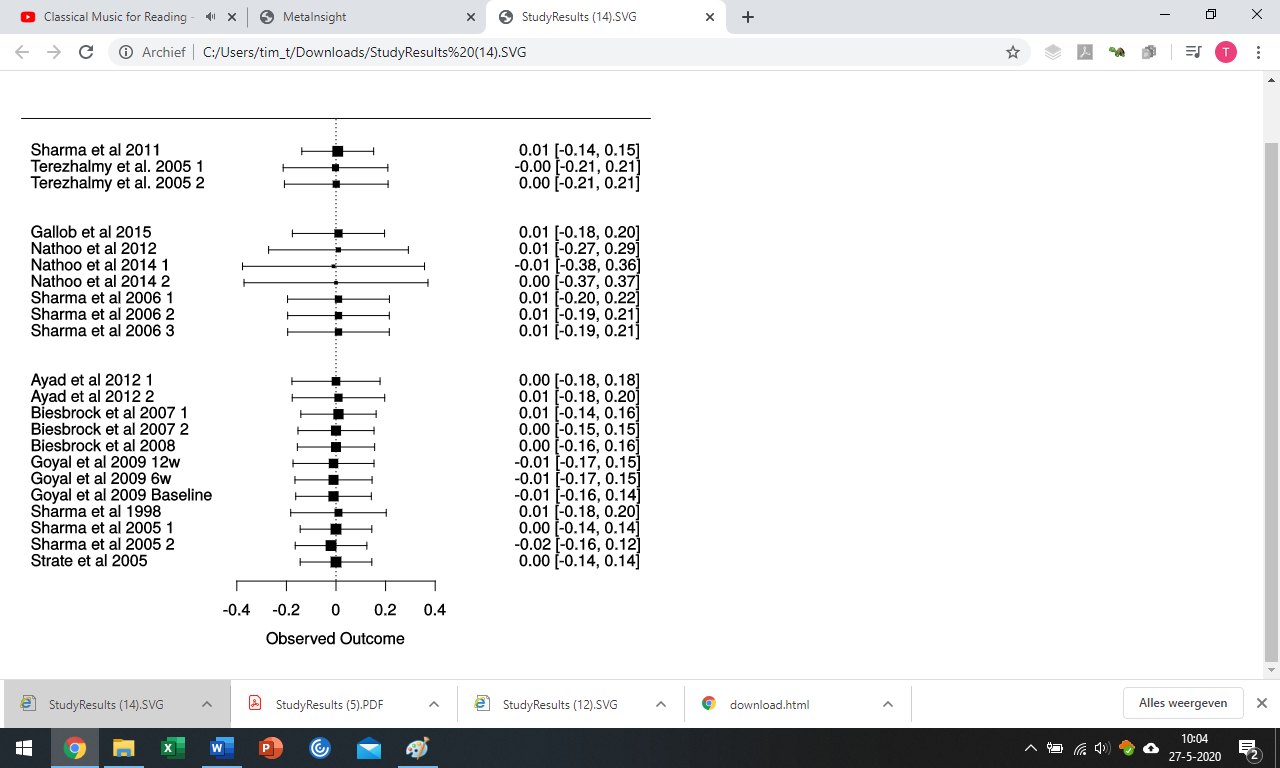


**Appendix S7b**. Forest plot of the sub analysis comparing OR and HFS with the MTB. RMNPI. *Pre-Brushing*


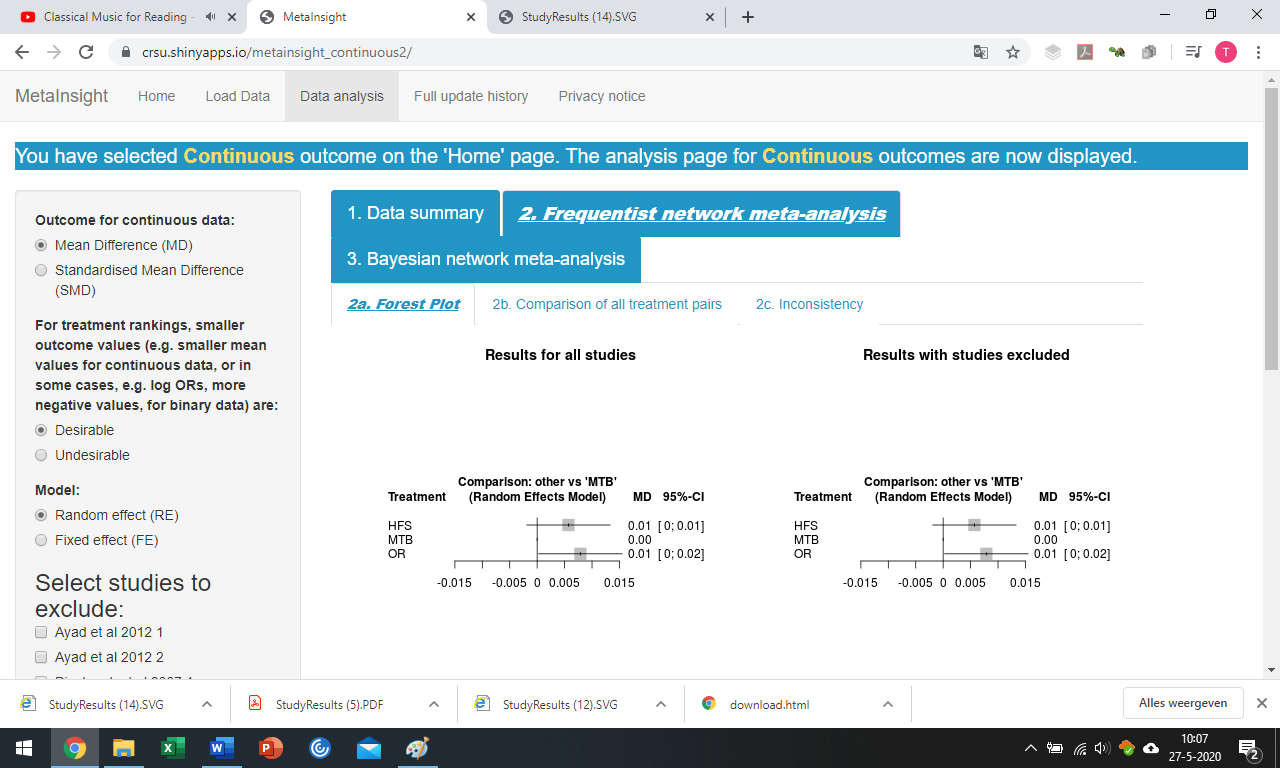


**
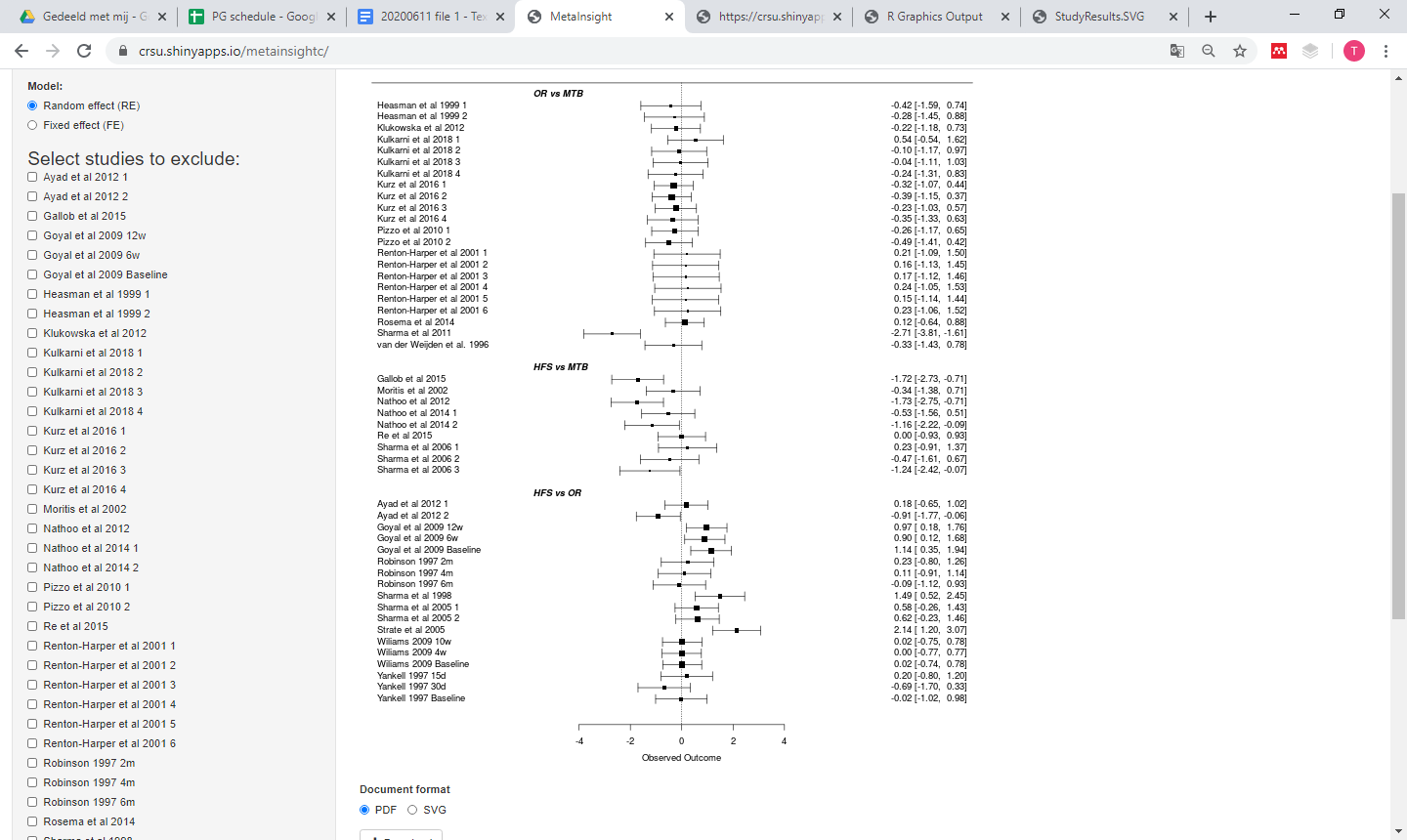
Appendix S8a**. Forest plot of the overall analysis of the comparisons at post-brushing.

**Appendix S8b**. Treatments are ranked from best to worst along the leading diagonal. Above the leading diagonal are estimates from pairwise meta-analyses (Direct comparison), below the leading diagonal are estimates from network meta-analyses (presented as NMA estimates (95%CI)). Overall results presented irrespective of indices used. *Post-Brushing*

| OR | -0.39 [-0.70; -0.08] | -0.23 [-0.52; -0.07] |
| --- | --- | --- |
| -0.16 [-0.43; -0.11] | HFS | -0.77 [-1.24; -0.31] |
| -0.43 [-0.70; -0.17] | -0.27 [-0.59; -0.04] | MTB |

**Appendix S8c.** Forest plot comparing OR and HFS with the MTB. *Post-Brushing*


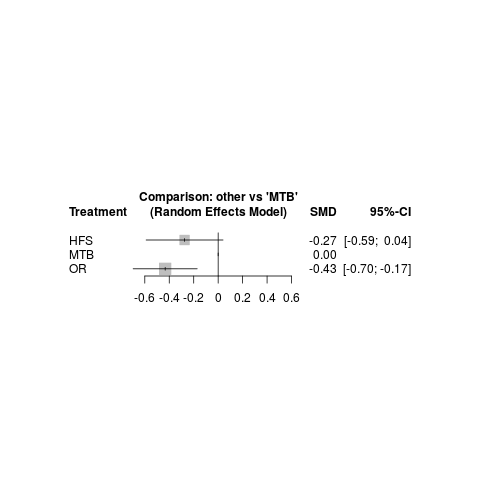


**
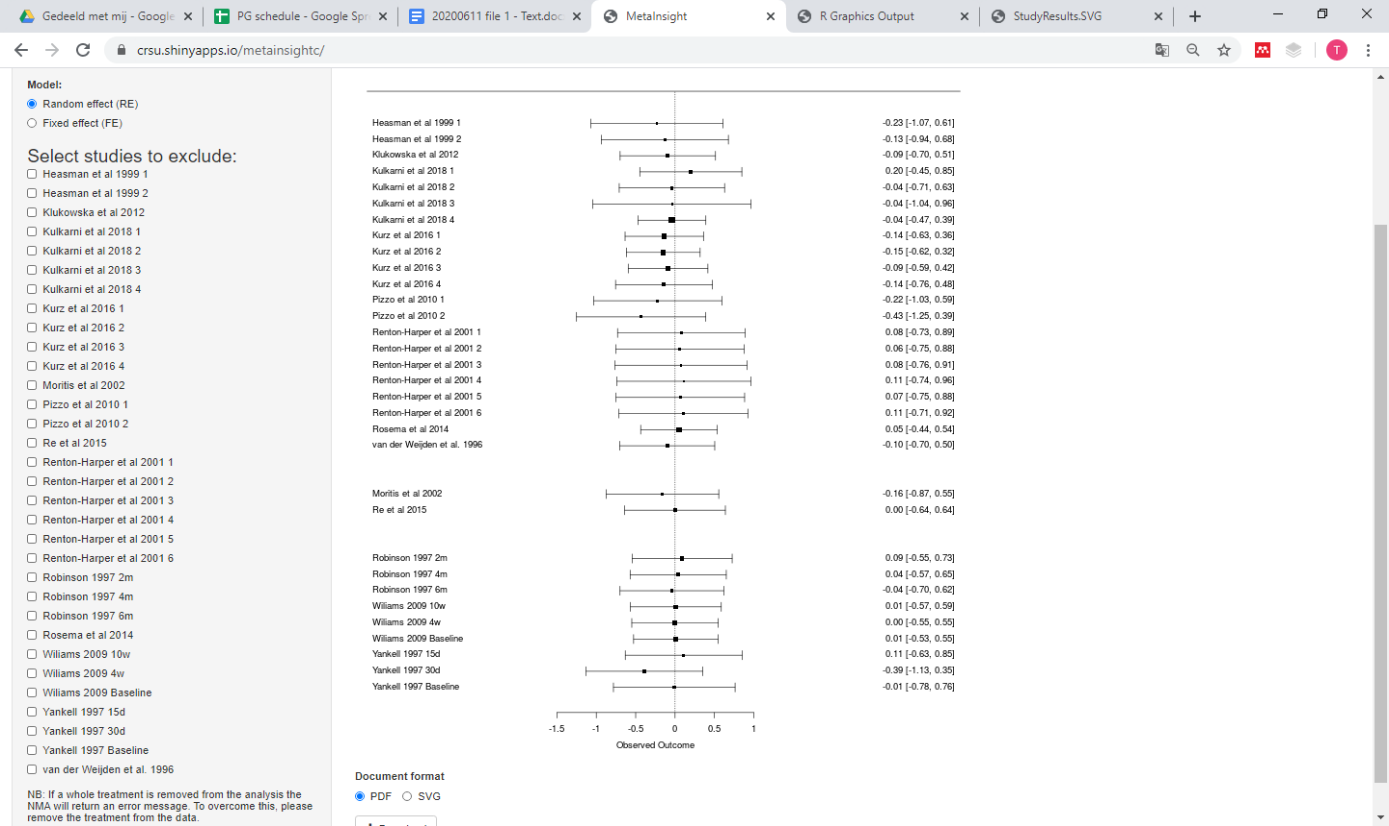
Appendix S9a**. Forest plot of the sub analysis of the comparisons. (M)QHPI. *Post-brushing*

**Appendix S9b**. Treatments are ranked from best to worst along the leading diagonal. Above the leading diagonal are estimates from pairwise meta-analyses (Direct comparison), below the leading diagonal are estimates from network meta-analyses (presented as NMA estimates (95%CI)). Sub-analysis on (M)Q&HPI. *Post-Brushing*

| HFS | -0.00 [-0.07;0.07] | -0.06 [-0.24; -0.11] |
| --- | --- | --- |
| -0.00 [-0.07; 0.06] | OR | -0.06 [-0.11; -0.01] |
| -0.06 [-0.14;-0.01] | -0.06 [-0.11; -0.02] | MTB |

**Appendix S9c**. Forest plot comparing OR and HFS with the MTB. Sub-analysis on (M)Q&HPI. *Post-Brushing*

**
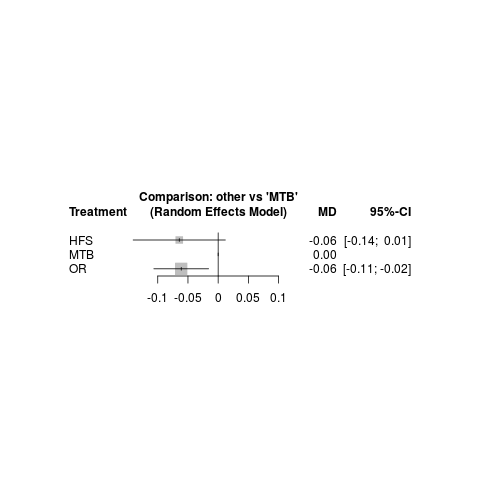
**

**Appendix S9d**. Specific **Ranking table for all studies using (M)Q&HPI- Probability for each treatment to be the best.** *Post-Brushing*


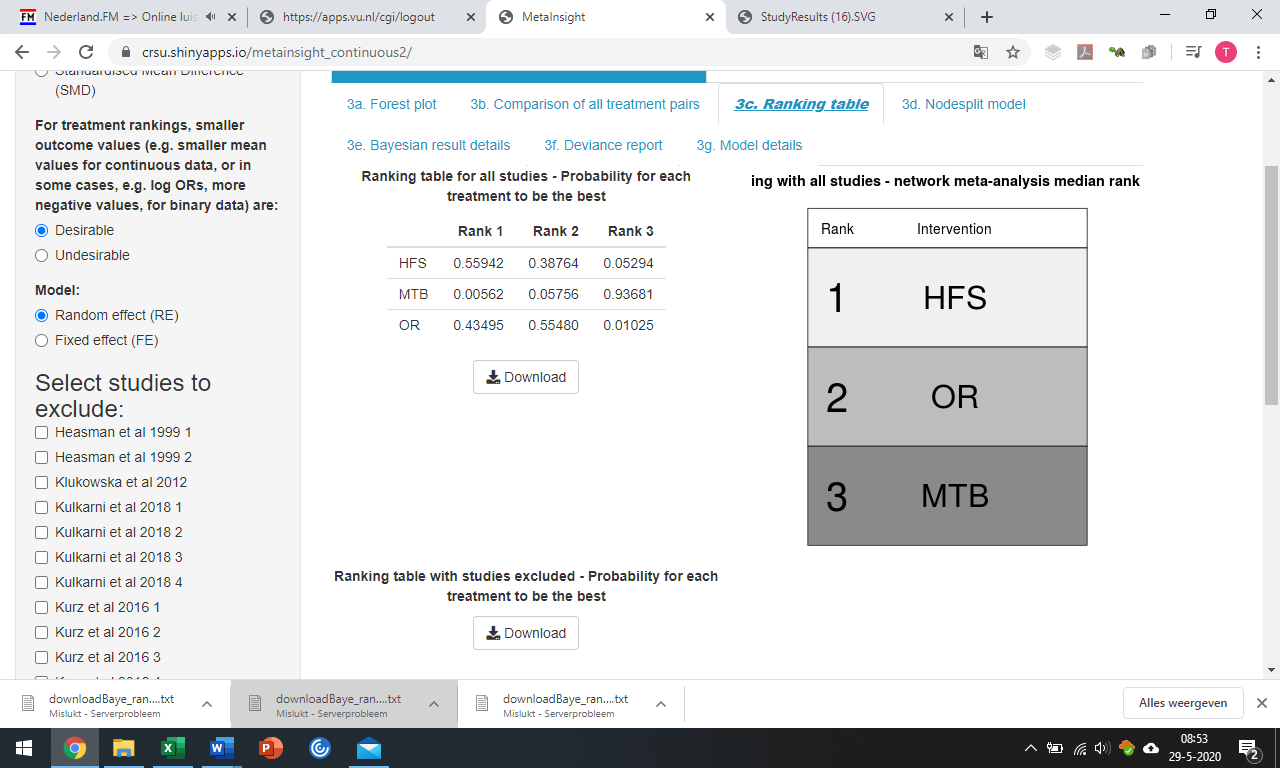


|  | Rank 1 | Rank 2 | Rank 3 |
| --- | --- | --- | --- |
| HFS | 0.53 | 0.42 | 0.05 |
| MTB | 0.00 | 0.06 | 0.94 |
| OR | 0.47 | 0.53 | 0.01 |

**Appendix S10a**. Forest plot of the sub analysis of the comparisons. RMNPI. *Post-brushing*


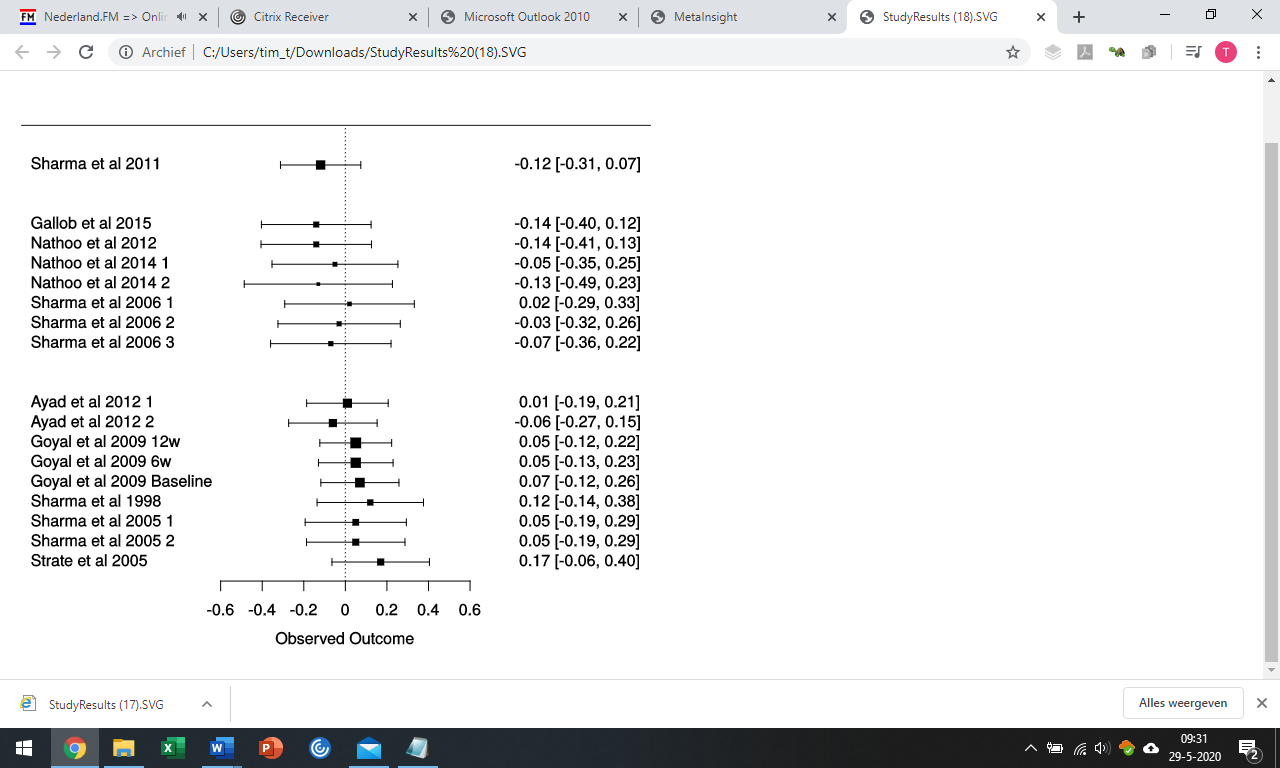


**Appendix S10b**. Treatments are ranked from best to worst along the leading diagonal. Above the leading diagonal are estimates from pairwise meta-analyses (Direct comparison), below the leading diagonal are estimates from network meta-analyses (presented as NMA estimates (95%CI)). Sub-analysis on RMNPI. *Post-Brushing*

| OR | -0.06 [-0.09; -0.02] | -0.12 [-0.22; -0.02] |
| --- | --- | --- |
| -0.05 [-0.09; -0.02] | HFS | -0.08 [-0.12; -0.04] |
| -0.13 [-0.18; -0.08] | -0.08 [-0.11; -0.04] | MTB |

**Appendix S10c**. Forest plot comparing OR and HFS with the MTB. Sub-analysis on RMNPI. *Post-Brushing*


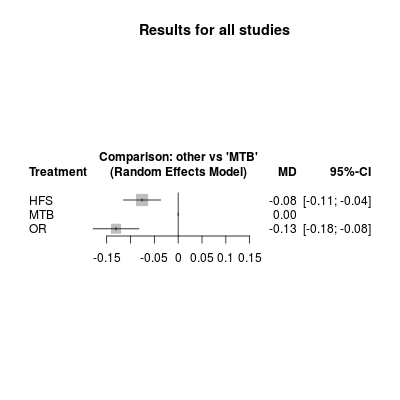


**Appendix S10d**. Specific **Ranking table for all studies using RMNPI- Probability for each treatment to be the best.** *Post-Brushing*


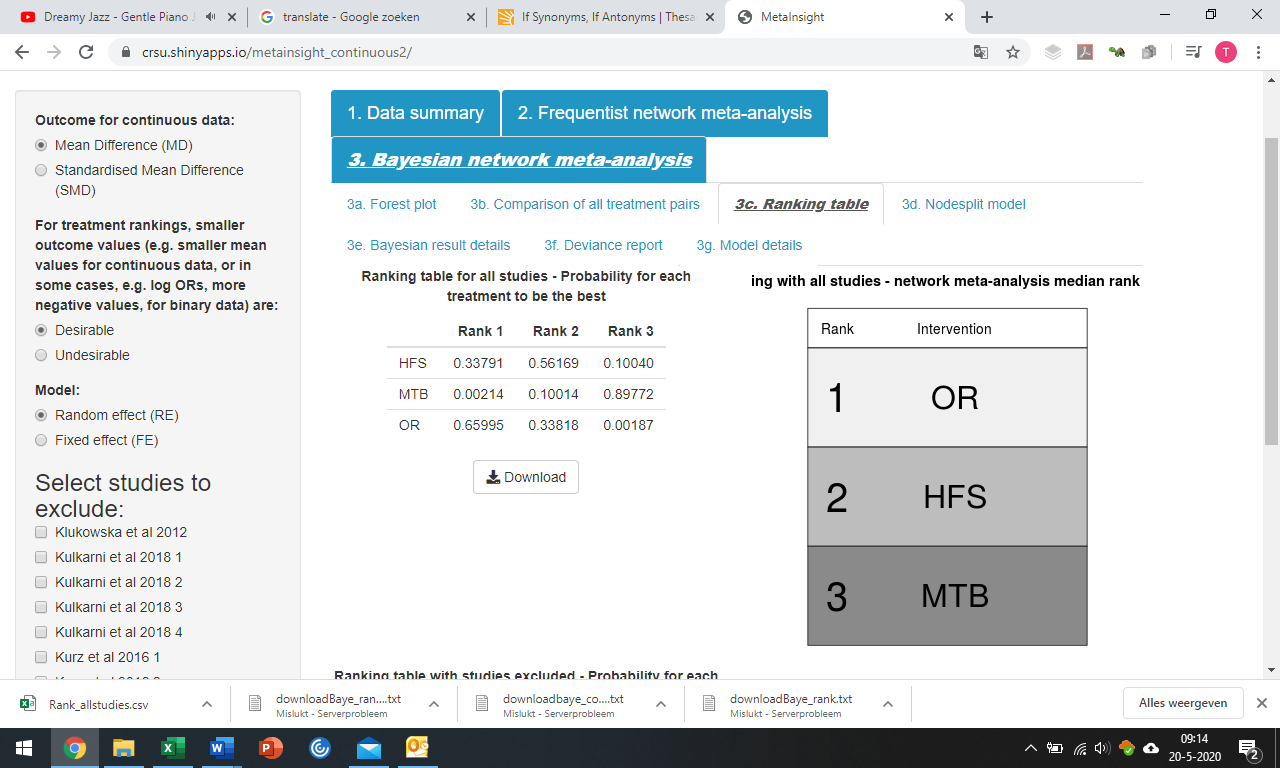


|  | Rank 1 | Rank 2 | Rank 3 |
| --- | --- | --- | --- |
| HFS | 0.00 | 0.99 | 0.00 |
| MTB | 0.00 | 0.00 | 1.00 |
| OR | 0.99 | 0.00 | 0.00 |

**Appendix S11a**. Forest plot of the overall analysis of the comparisons of the change in plaque scores, irrespective of the plaque indices used.


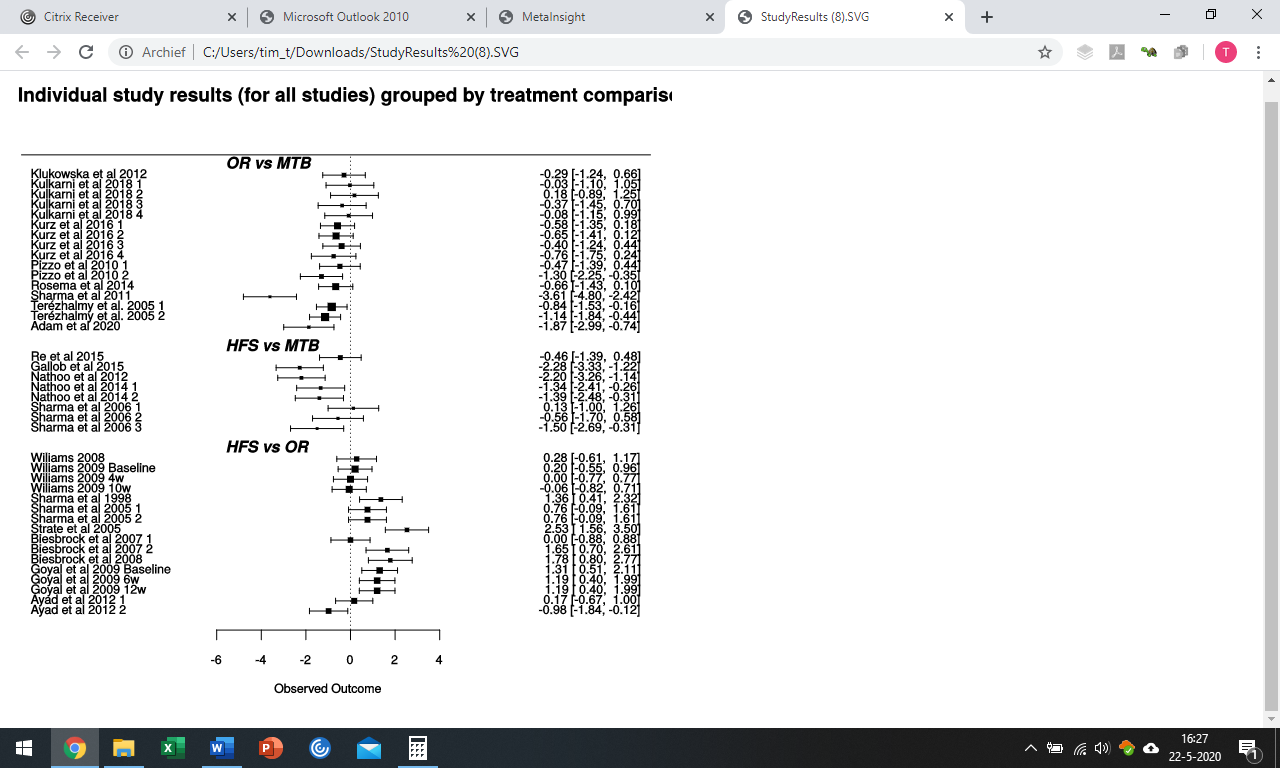


**Appendix S11b.** Treatments are ranked from best to worst along the leading diagonal. Above the leading diagonal are estimates from pairwise meta-analyses (Direct comparison), below the leading diagonal are estimates from network meta-analyses (presented as NMA estimates (95%CI)). Overall results presented irrespective of indices used. *Incremental reduction between pre- and post brushing*

| OR | -0.75 [-1.12; -0.38] | -0.79 [-1.17; -0.41] |
| --- | --- | --- |
| -0.47 [-0.80; -0.15] | HFS | -1.20 [-1.75; -0.65] |
| -1.07 [-1.40; -0.74] | -0.60 [-0.98; -0.21] | MTB |

**Appendix S11c**. Forest plot comparing OR and HFS with the MTB. *Incremental reduction between pre- and post brushing*


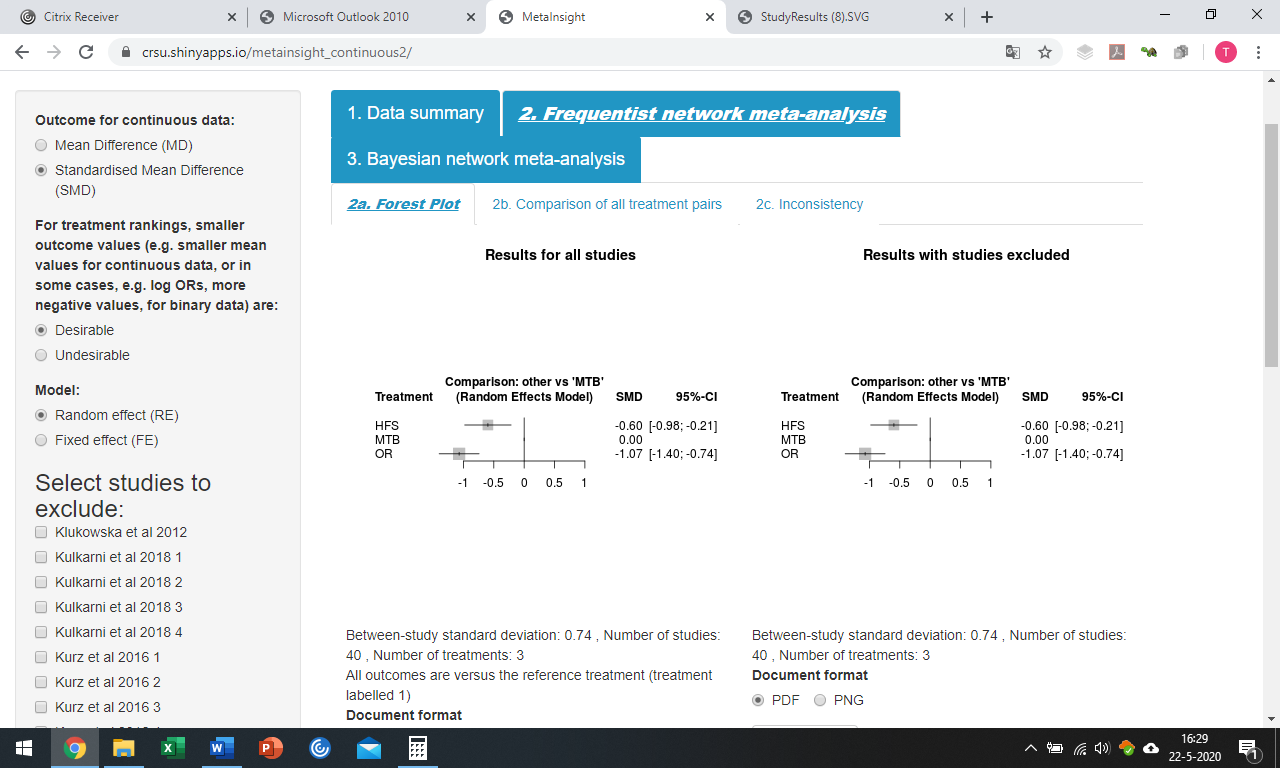


**Appendix S12a.** Forest plot of the overall analysis of the comparisons of the change in plaque scores on the (M)Q&HPI


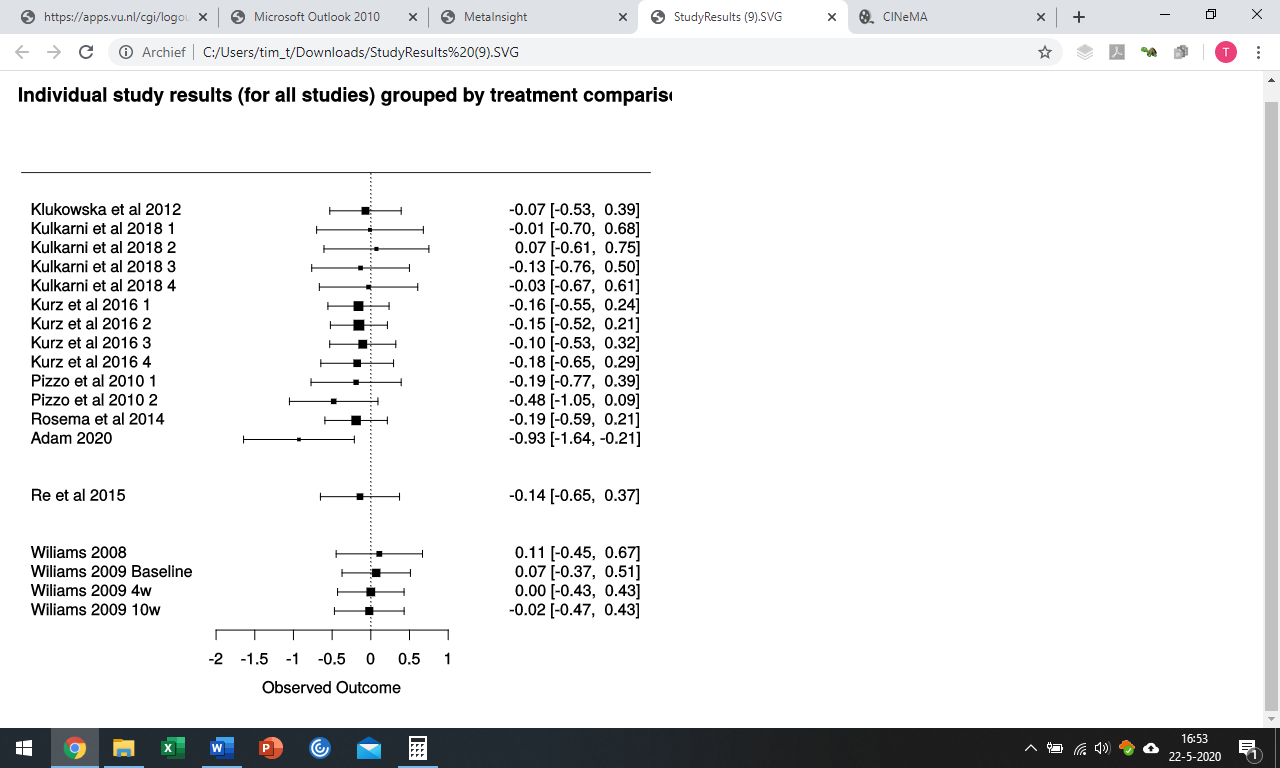


**Appendix S12b**. Treatments are ranked from best to worst along the leading diagonal. Above the leading diagonal are estimates from pairwise meta-analyses (Direct comparison), below the leading diagonal are estimates from network meta-analyses (presented as NMA estimates (95%CI)). Sub-analysis on (M)Q&HPI. *Incremental reduction between pre- and post brushing*

| OR | -0.04 [-0.15; 0.08] | -0.18 [-0.25; -0.11] |
| --- | --- | --- |
| -0.04 [-0.14; 0.07] | HFS | -0.14 [-0.38; 0.10] |
| -0.18 [-0.25; -0.11] | -0.14 [-0.26; -0.03] | MTB |

**Appendix S12c.** Forest plot comparing OR and HFS with the MTB. Sub-analysis on (M)Q&HPI. *Incremental reduction between pre- and post brushing*


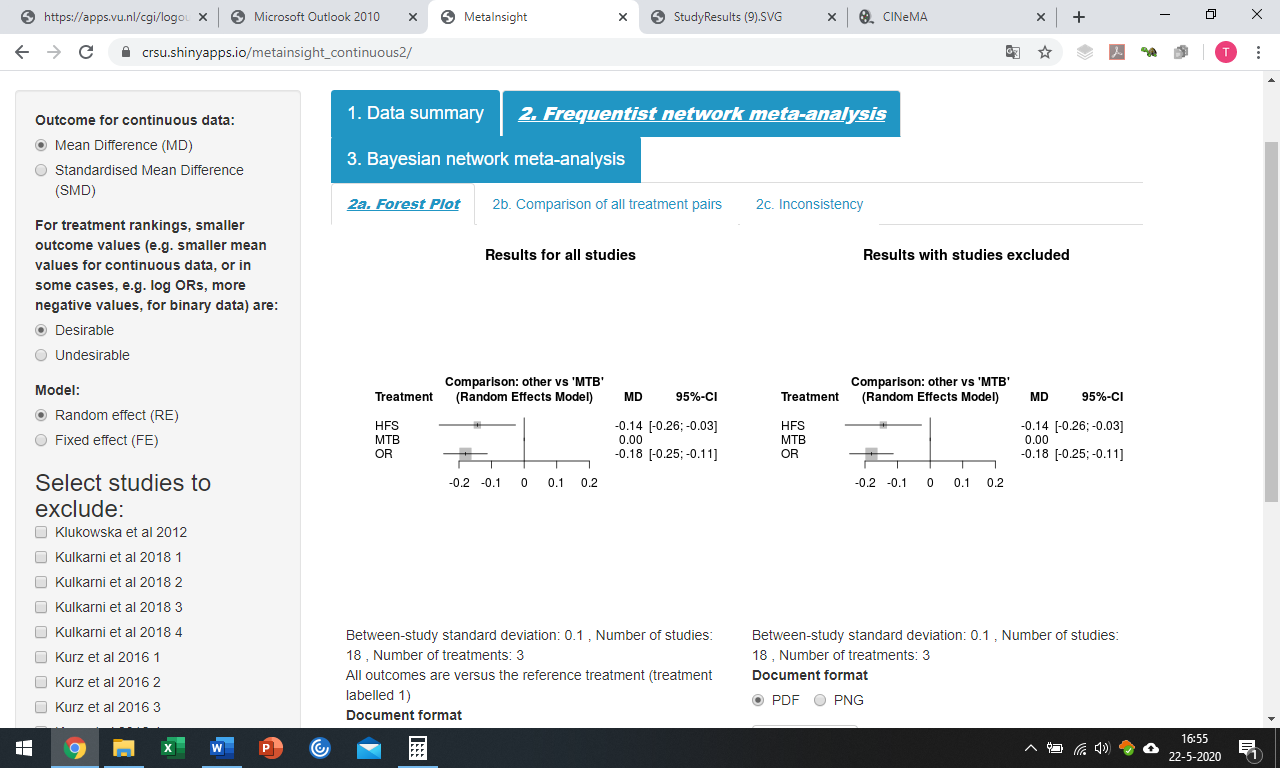


**Appendix S12d**. Specific **Ranking table for all studies using (M)Q&HPI- Probability for each treatment to be the best.** *Incremental reduction between pre- and post brushing*


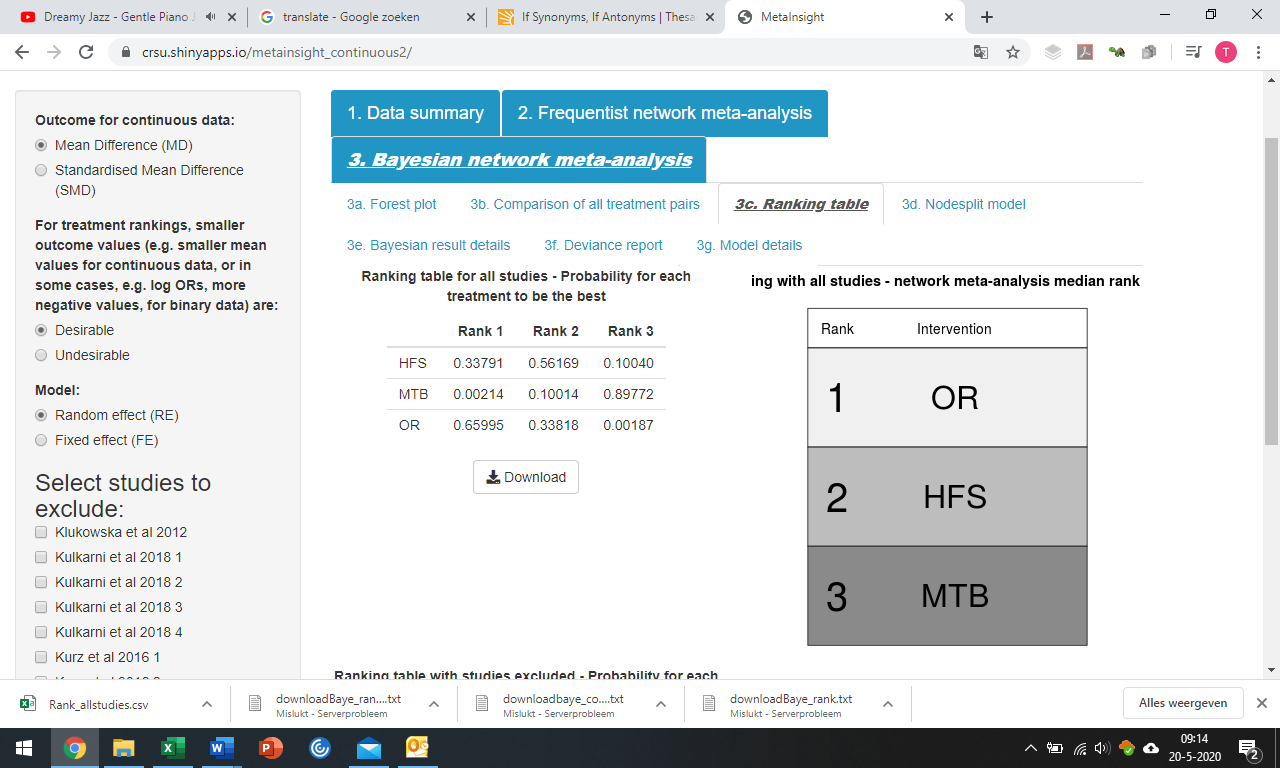


|  | Rank 1 | Rank 2 | Rank 3 |
| --- | --- | --- | --- |
| HFS | 0.31 | 0.64 | 0.05 |
| MTB | 0.00 | 0.05 | 0.95 |
| OR | 0.69 | 0.30 | 0.00 |

**Appendix S13a.** Forest plot of the overall analysis of the comparisons of theincremental reduction in plaque scores on the RMNPI.


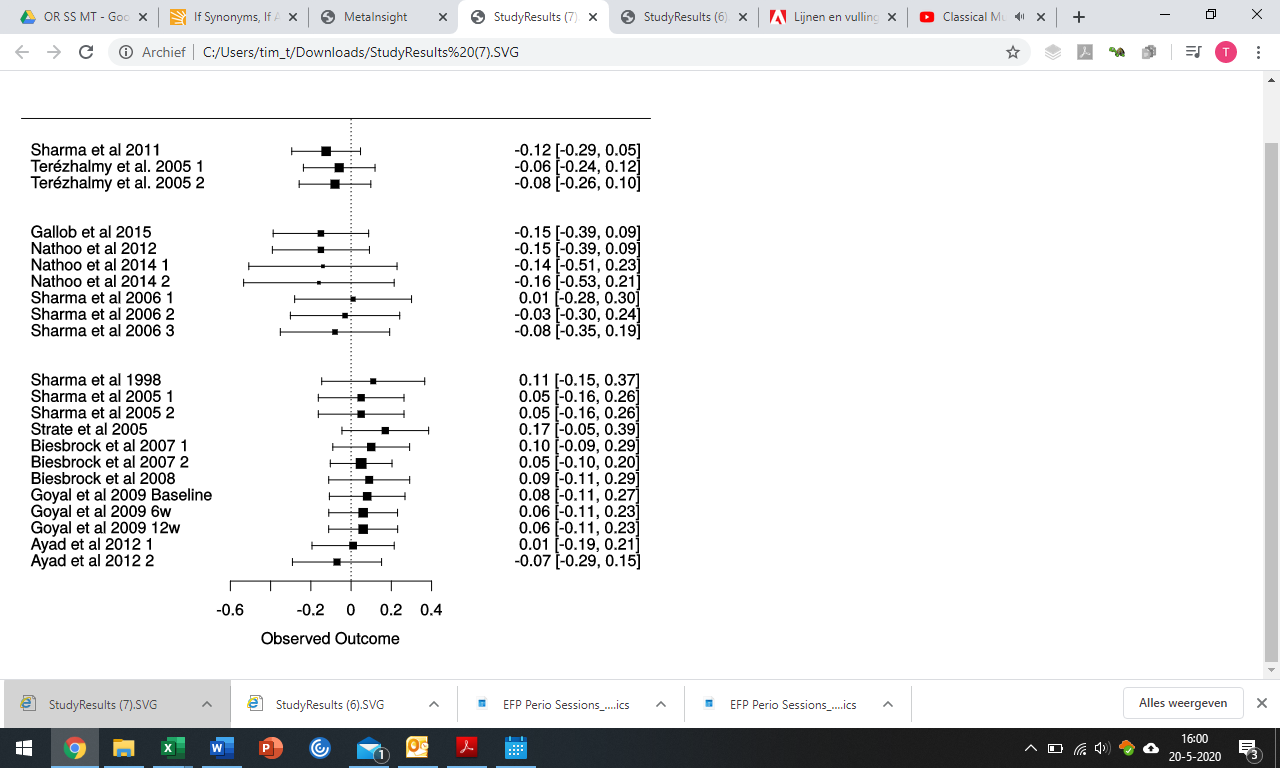


**Appendix S13b.** Treatments are ranked from best to worst along the leading diagonal. Above the leading diagonal are estimates from pairwise meta-analyses (Direct comparison), below the leading diagonal are estimates from network meta-analyses (presented as NMA estimates (95%CI)). Sub-analysis on RMNPI. *Incremental reduction between pre- and post brushing*

| OR | -0.06 [-0.09; -0.04] | -0.09 [-0.14; -0.03] |
| --- | --- | --- |
| -0.05 [-0.08; -0.03] | HFS | -0.10 [-0.14; -0.06] |
| -0.13 [-0.17; -0.09] | -0.08 [-0.11; -0.04] | MTB |

**Appendix S13c**. Forest plot comparing OR and HFS with the MTB. Sub-analysis on RMNPI. *Incremental reduction between pre- and post brushing*


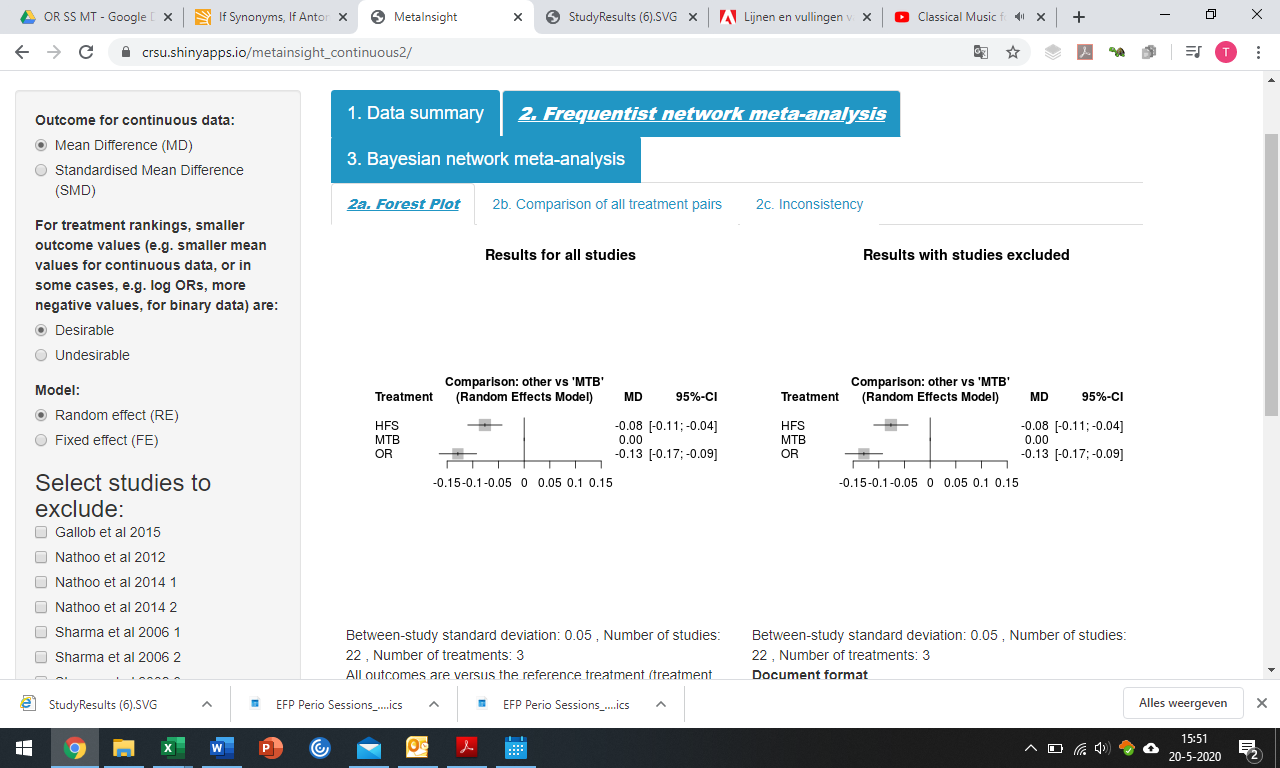


**Appendix S13d**. Specific **Ranking table for all studies using RMNPI- Probability for each treatment to be the best.** *Incremental reduction between pre- and post brushing*


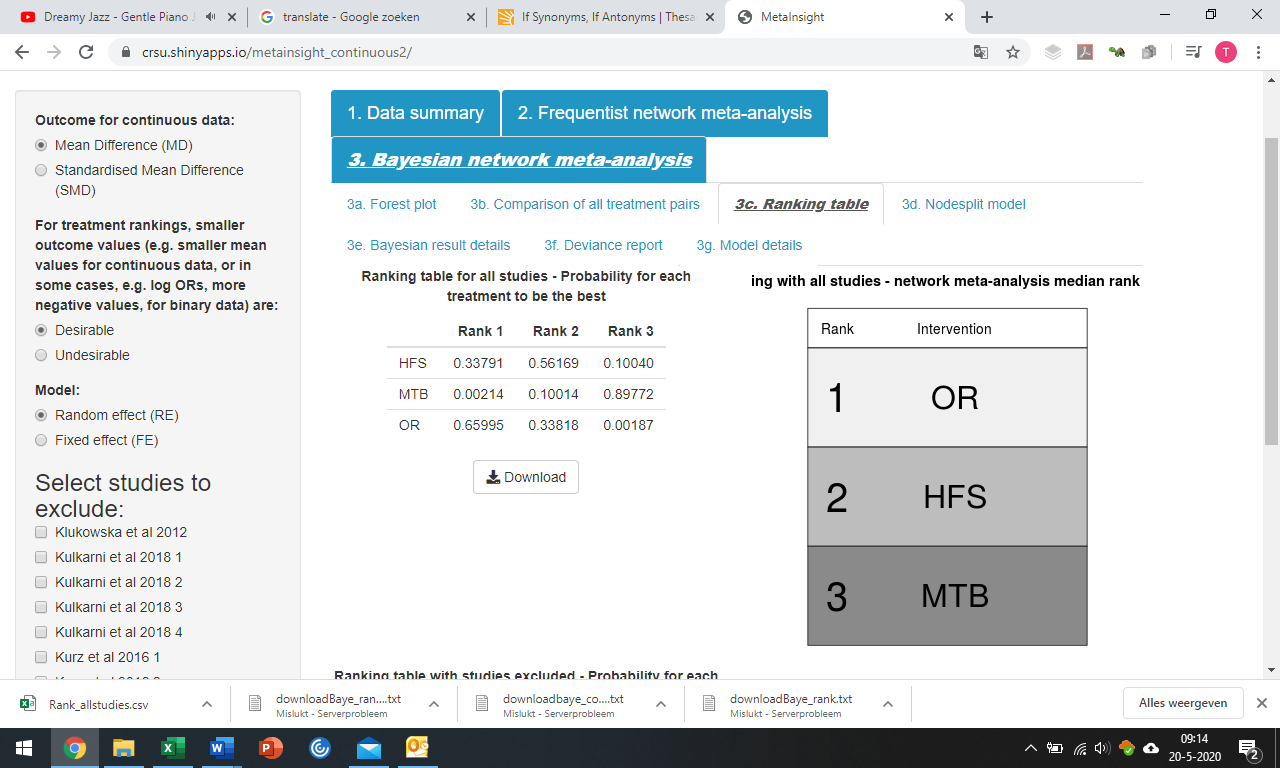


|  | Rank 1 | Rank 2 | Rank 3 |
| --- | --- | --- | --- |
| HFS | 0.00 | 1.00 | 0.00 |
| MTB | 0.00 | 0.00 | 1.00 |
| OR | 1.00 | 0.00 | 0.00 |

**Appendix S14**. MetaInsight Network meta-analysis graphs. Each node represents a device, manual toothbrush (MTB), an oscillating-rotating power toothbrush (OR) an a high frequency sonic power toothbrush (HFS). Each line between the nodes show the amount of comparisons. *Post-Brushing*

**Appendix S14a**: Network meta-analysis graph irrespective of the plaque indices. *Post-Brushing*

**Appendix S14b**: Network meta-analysis graph using only the (M)Q&HPI. *Post-Brushing*

**Appendix S14c**: Network meta-analysis graph using only the RMNPI. *Post-Brushing*


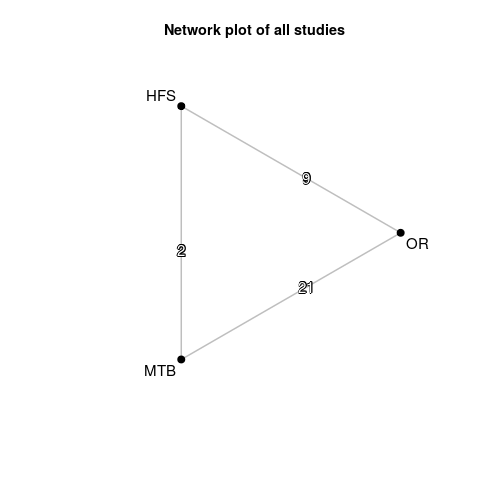

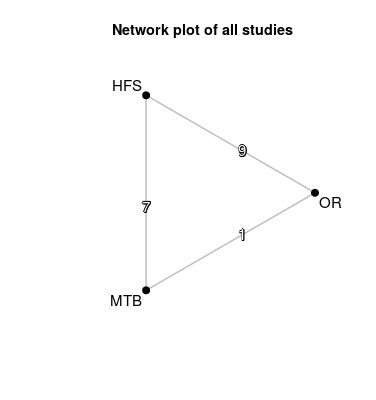

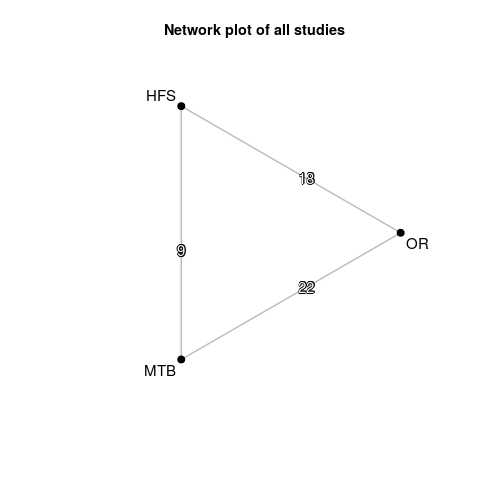
S14a S14b S14c

**Appendix S15**. MetaInsight Network meta-analysis graphs. Each node represents a device, manual toothbrush (MTB), an oscillating-rotating power toothbrush (OR) an a high frequency sonic power toothbrush (HFS). Each line between the nodes show the amount of comparisons. *Incremental reduction between pre- and post brushing*

**Appendix S15a:** Network meta-analysis graph irrespective of the plaque indices. *Difference*

**Appendix S15b**: Network meta-analysis graph using only the (M)Q&HPI. *Difference*

**Appendix S15c**: Network meta-analysis graph using only the RMNPI. *Difference*

S15a S15b S15c


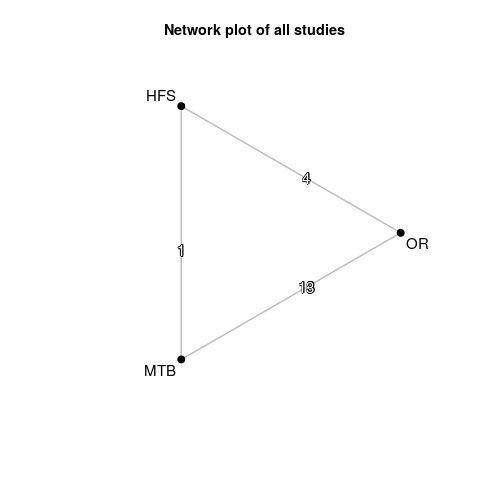

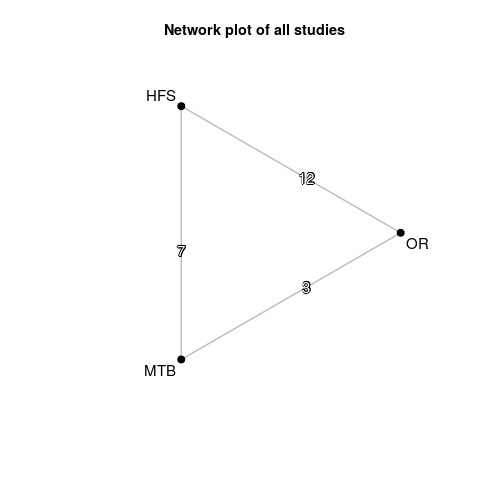

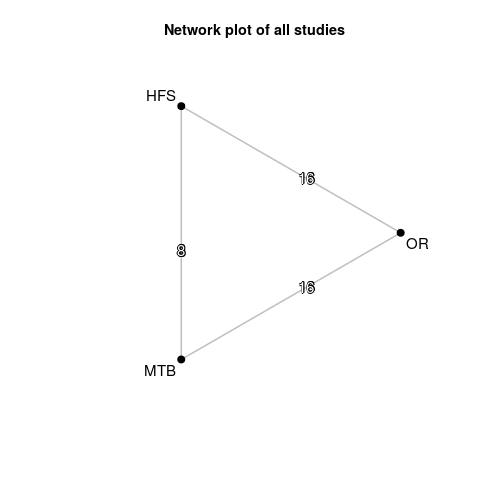


**Appendix S16a**. Confidence in Network Meta-Analysis (CINeMA). Confidence Rating. Overall. *Post-Brushing*

| Comparison | Number of studies | Within-study bias | Reporting bias | Indirectness | Imprecision | Heterogeneity | Incoherence | Confidence rating |
| --- | --- | --- | --- | --- | --- | --- | --- | --- |
| HFS:MTB | 8 | No concerns | Undetected | No concerns | Some concerns | Some concerns | Major concerns | Moderate |
| HFS:OR | 16 | No concerns | Undetected | Some concerns | Some concerns | Some concerns | Major concerns | Moderate |
| MTB:OR | 16 | Some concerns | Undetected | No concerns | No concerns | Major concerns | Some concerns | Moderate |

**Appendix S16b**. Confidence in Network Meta-Analysis (CINeMA). Risk of bias contributions. The bar chart shows the contributions of each piece of study to the network estimate. Overall. *Post-Brushing*


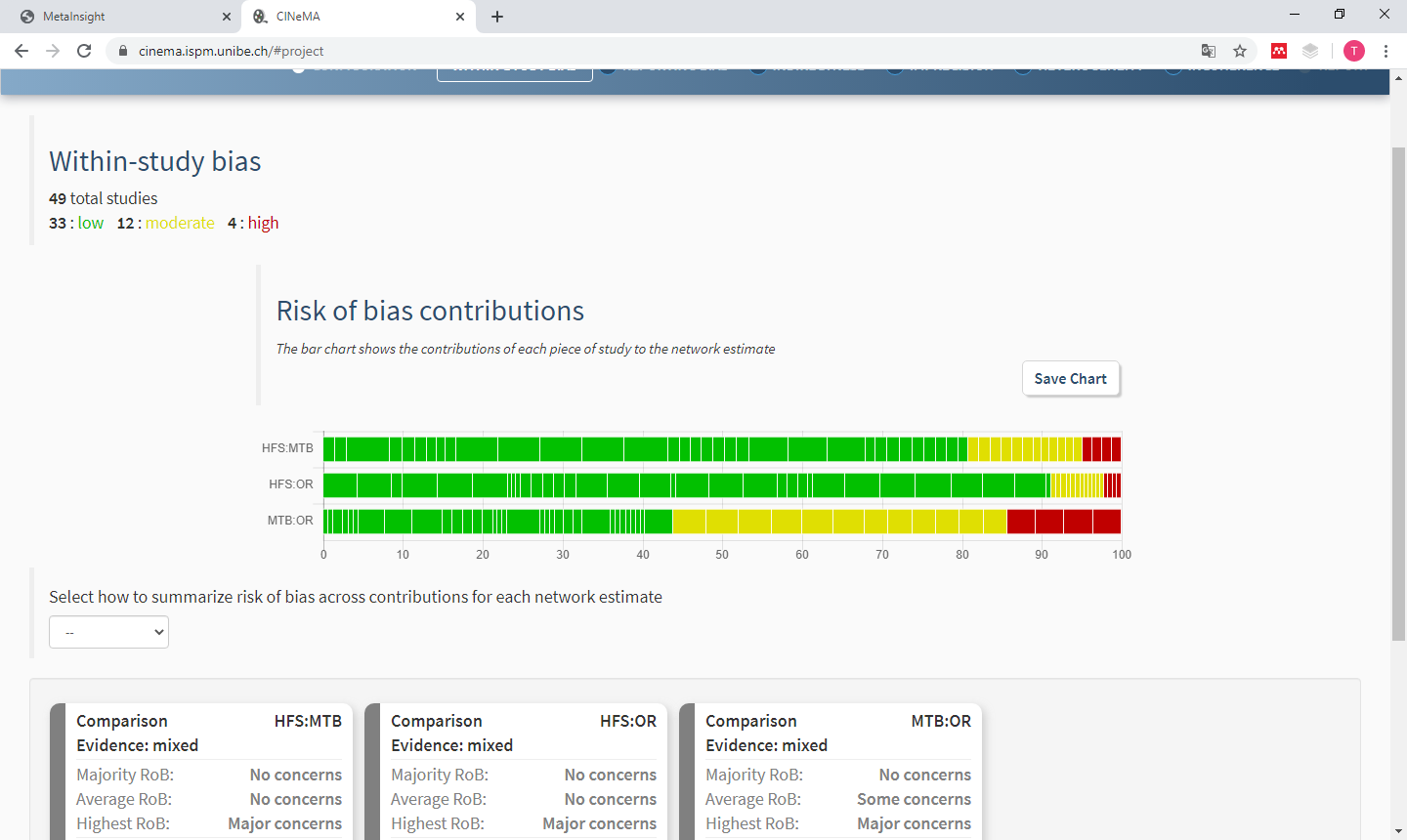


**Appendix S16c.** Confidence in Network Meta-Analysis (CINeMA). Reporting Bias. Overall. *Post-Brushing*


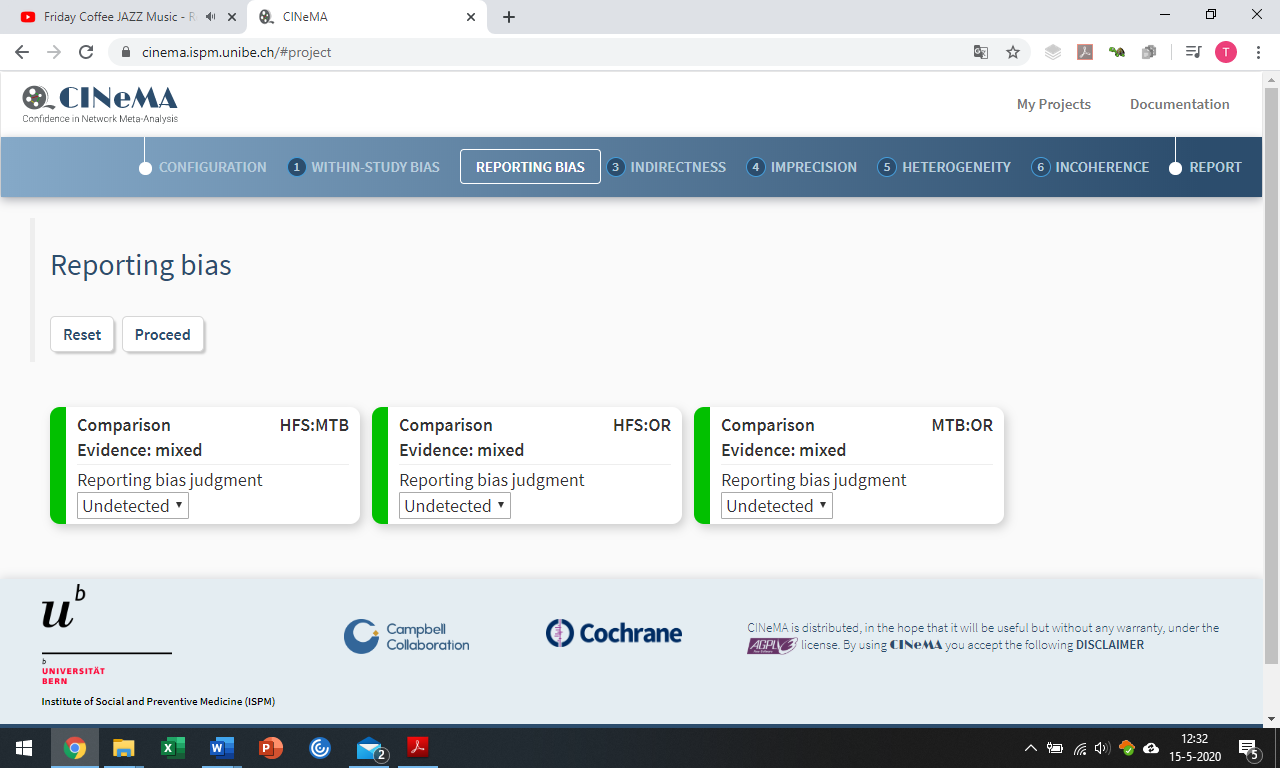


**Appendix S16d**. Confidence in Network Meta-Analysis (CINeMA). Indirectness contributions The bar chart shows the contributions of each study to the network estimate.Overall. *Post-Brushing*


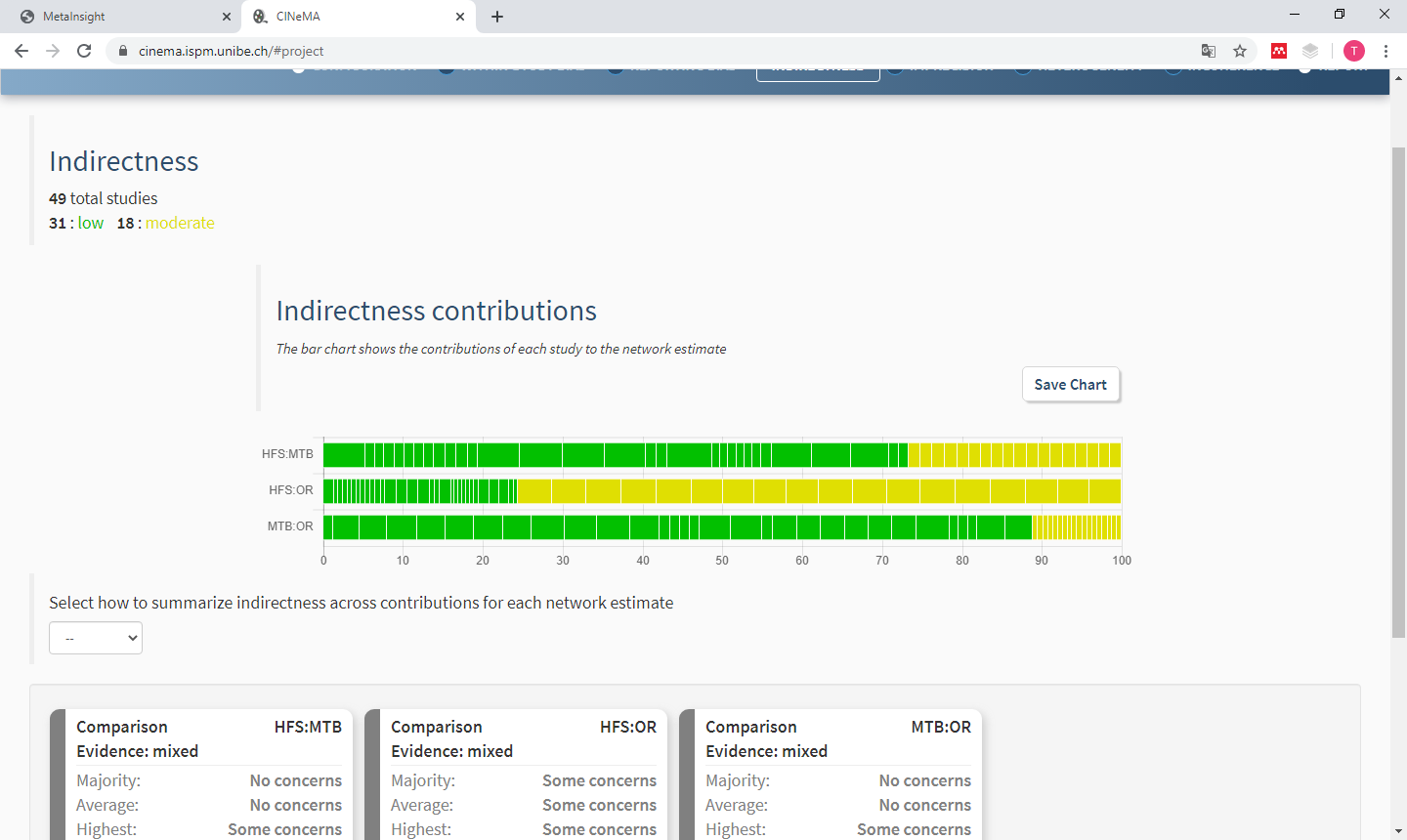


**Appendix S16e.** Confidence in Network Meta-Analysis (CINeMA). Imprecision.Overall. *Post-Brushing*


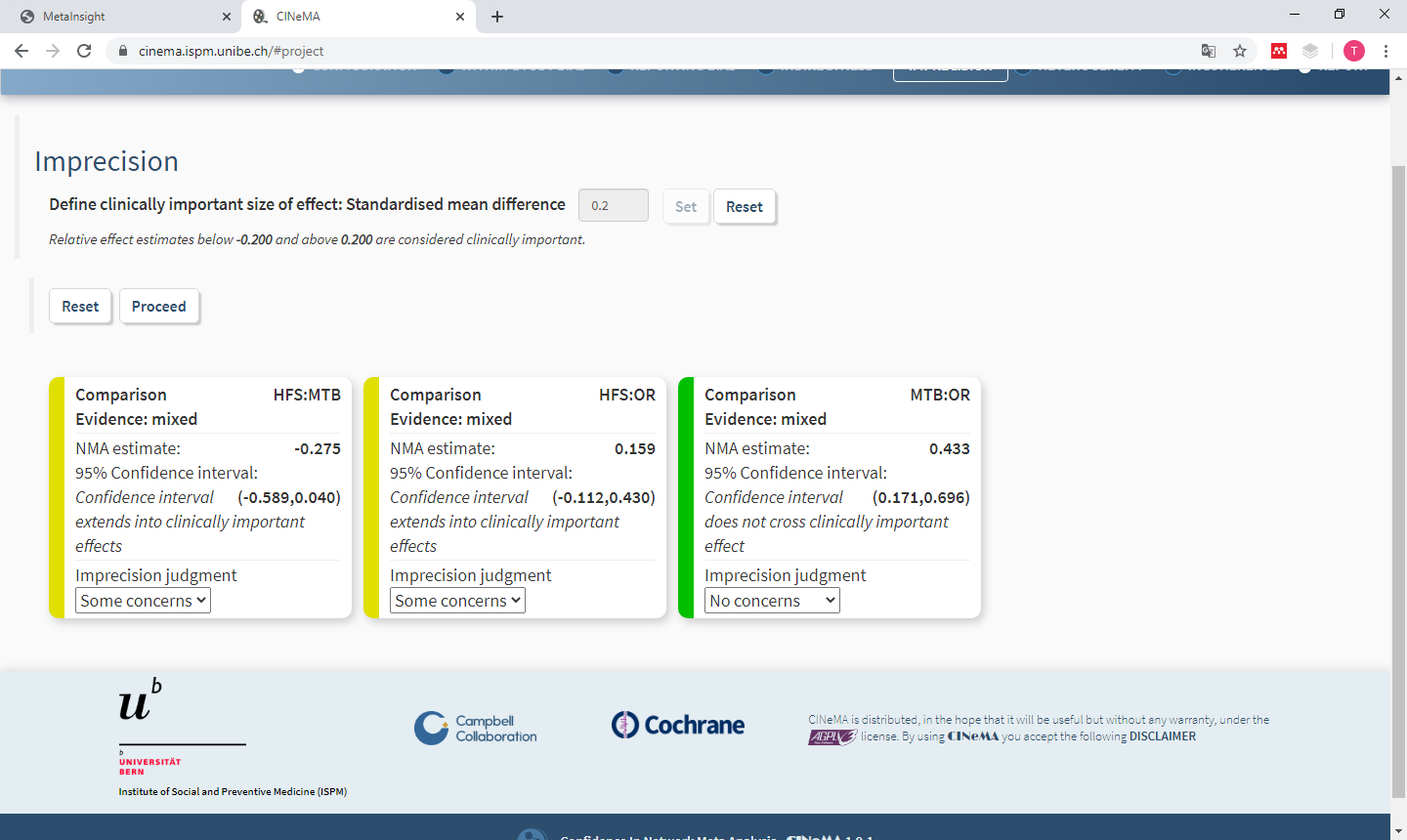


**Appendix S16f.** Confidence in Network Meta-Analysis (CINeMA). Heterogeneity.Overall. *Post-Brushing*


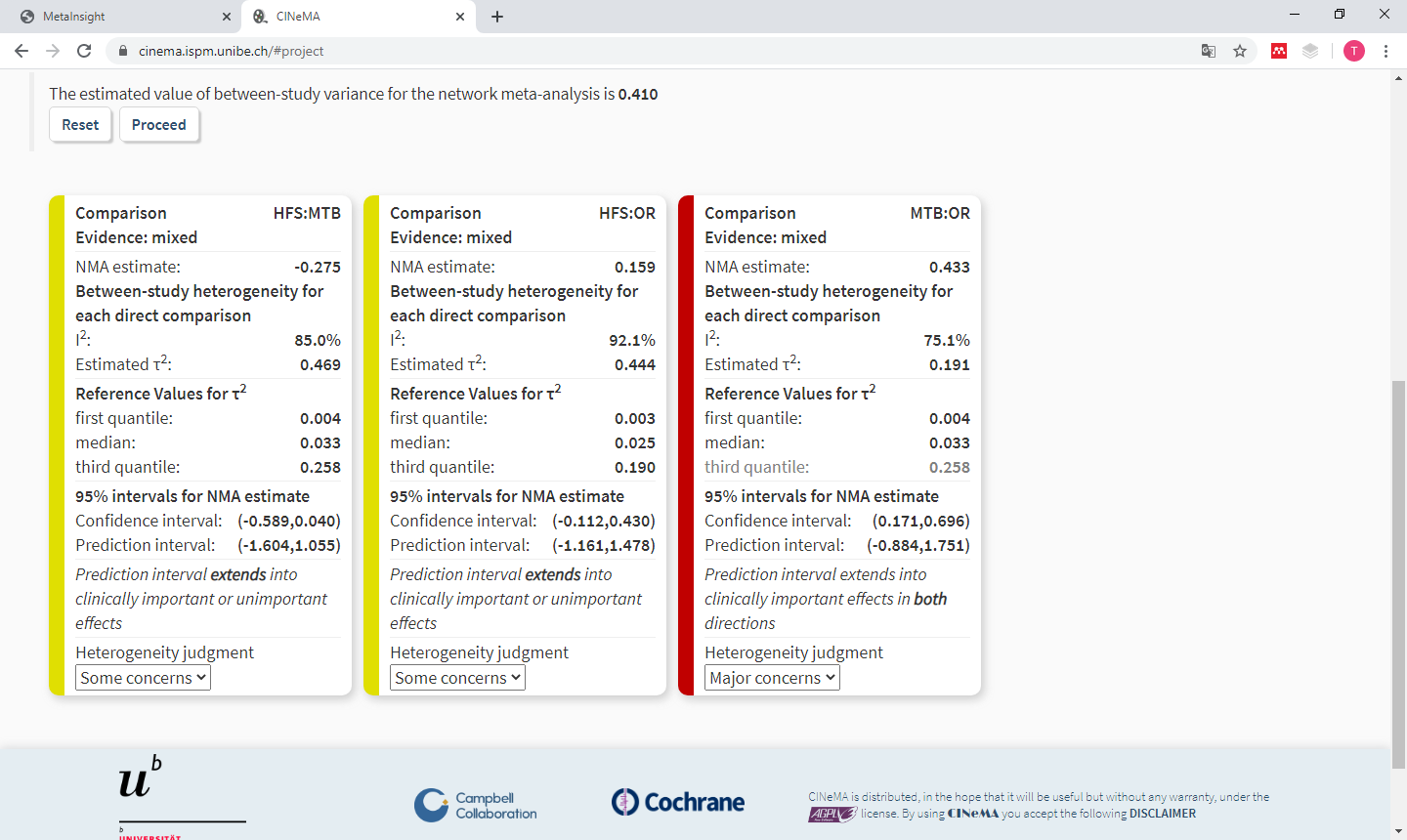


**Appendix S16g.** Confidence in Network Meta-Analysis (CINeMA). Incoherence. Separating indirect from direct evidence. Overall. *Post-Brushing*


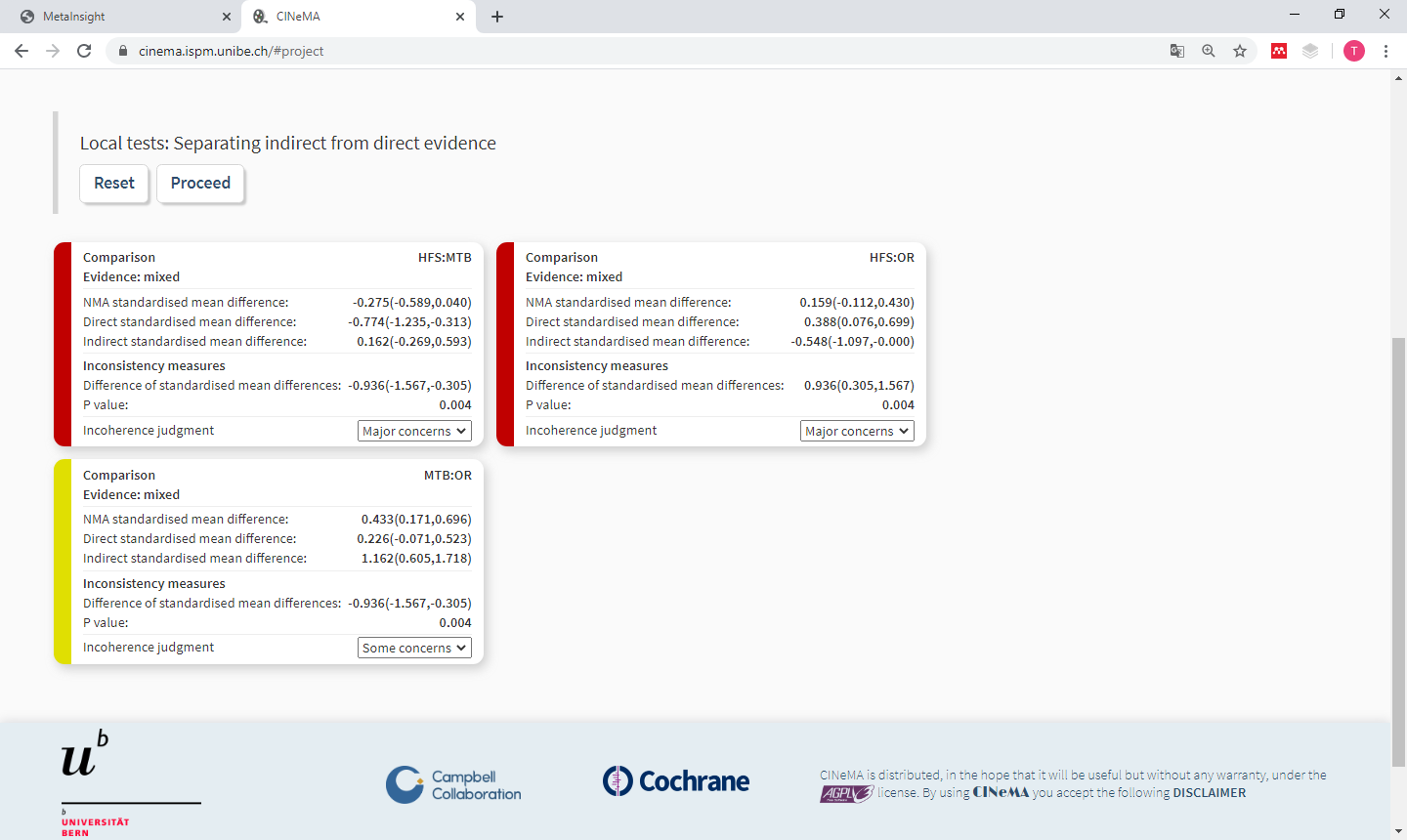


**Appendix S17a.** Confidence in Network Meta-Analysis (CINeMA). Confidence Rating. Sub-analysis (M)Q&HPI. *Post-Brushing*

| Comparison | Number of studies | Within-study bias | Reporting bias | Indirectness | Imprecision | Heterogeneity | Incoherence | Confidence rating |
| --- | --- | --- | --- | --- | --- | --- | --- | --- |
| HFS:MTB | 1 | No concerns | Undetected | No concerns | No concerns | No concerns | No concerns | High |
| HFS:OR | 4 | No concerns | Undetected | Some concerns | No concerns | No concerns | No concerns | High |
| MTB:OR | 13 | Some concerns | Undetected | No concerns | No concerns | No concerns | No concerns | High |

**Appendix S17b.** Confidence in Network Meta-Analysis (CINeMA). Risk of bias contributions. The bar chart shows the contributions of each piece of study to the network estimate. Sub-analysis (M)Q&HPI. *Post-Brushing*


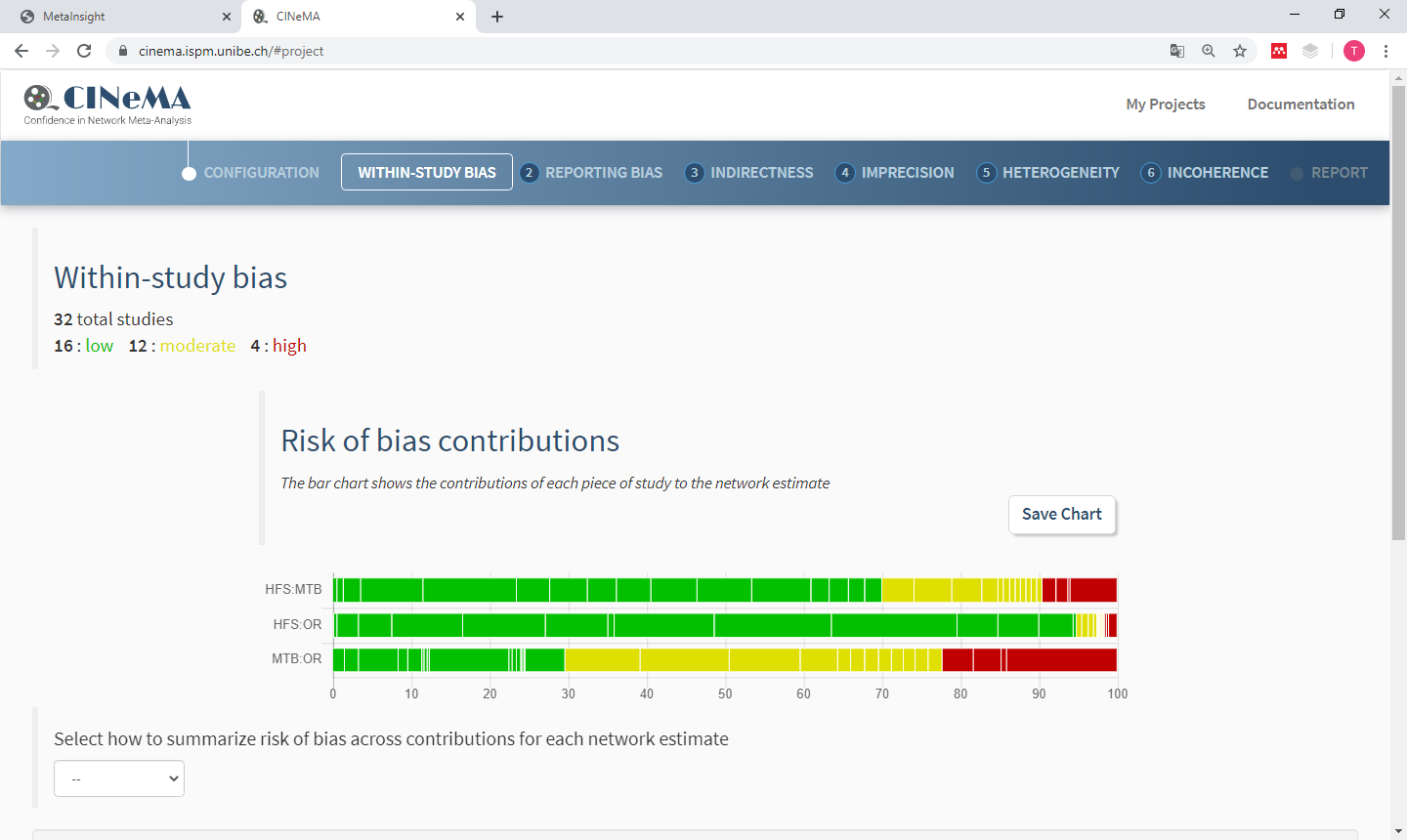


**Appendix S17c.** Confidence in Network Meta-Analysis (CINeMA). Reporting Bias. Sub-analysis (M)Q&HPI. *Post-Brushing*


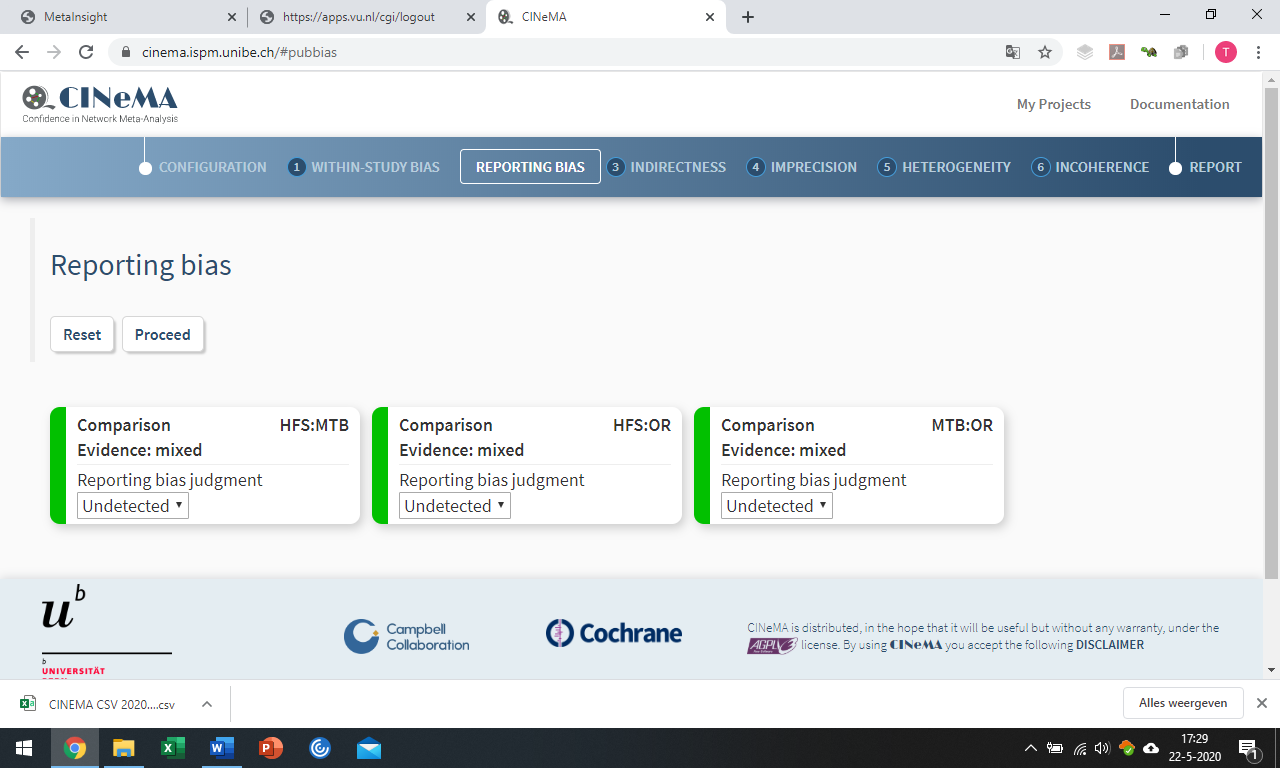


**
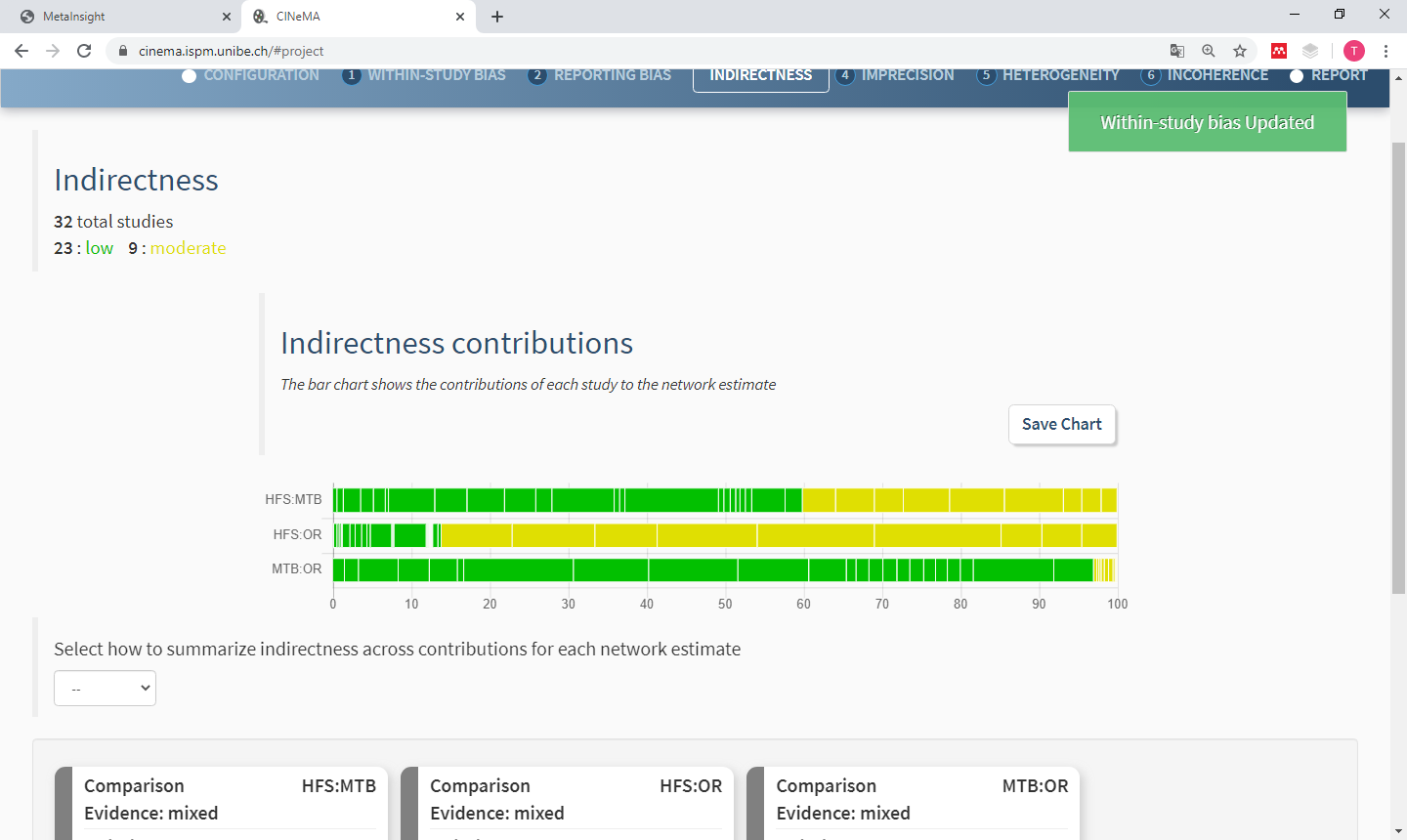
Appendix S17d.** Confidence in Network Meta-Analysis (CINeMA). Indirectness contributions The bar chart shows the contributions of each study to the network estimate. Sub-analysis (M)Q&HPI. *Post-Brushing*

**Appendix S17e**. Confidence in Network Meta-Analysis (CINeMA). Imprecision. Sub-analysis (M)Q&HPI. *Post-Brushing*


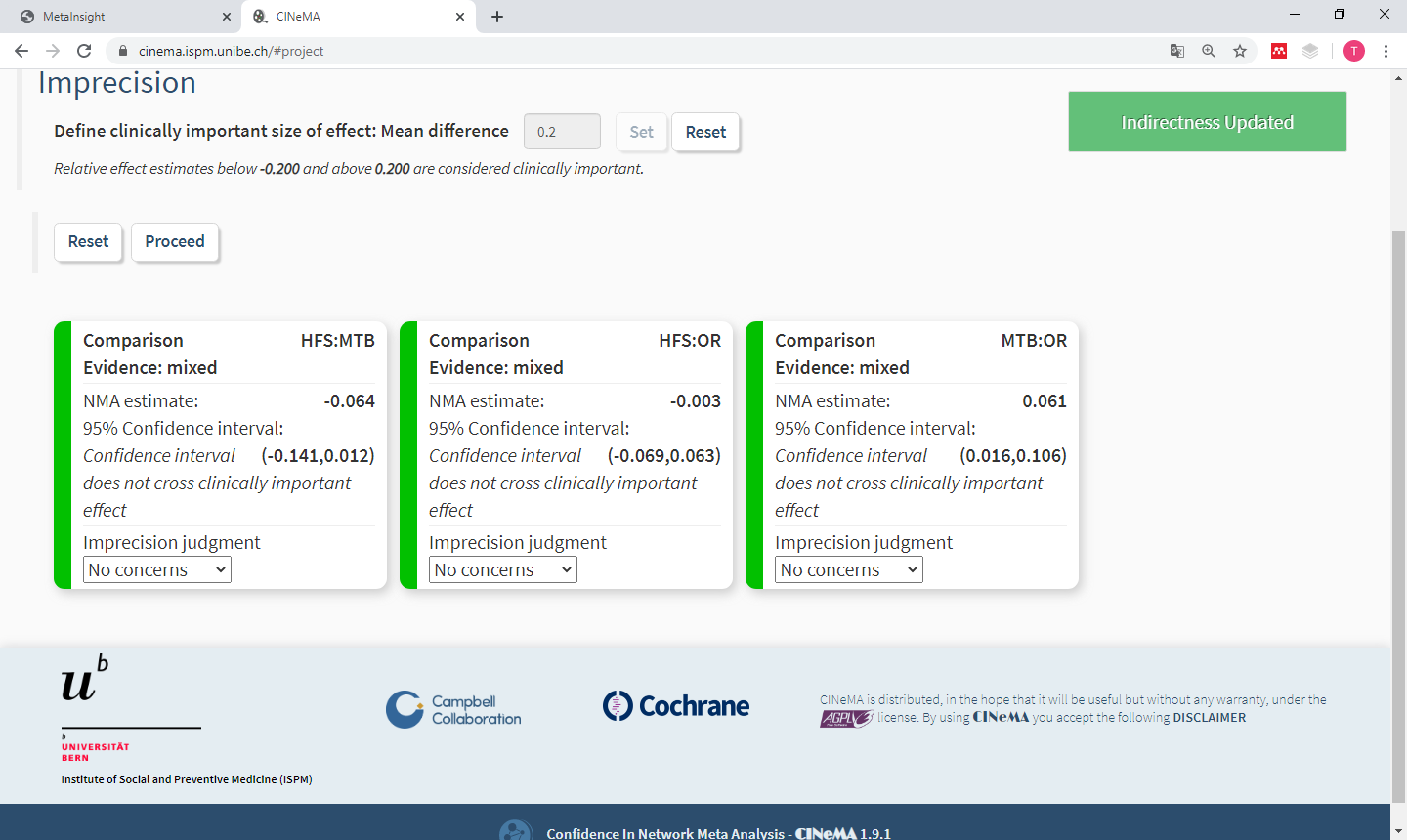


**Appendix S17f.** Confidence in Network Meta-Analysis (CINeMA). Heterogeneity. Sub-analysis (M)Q&HPI. *Post-Brushing*


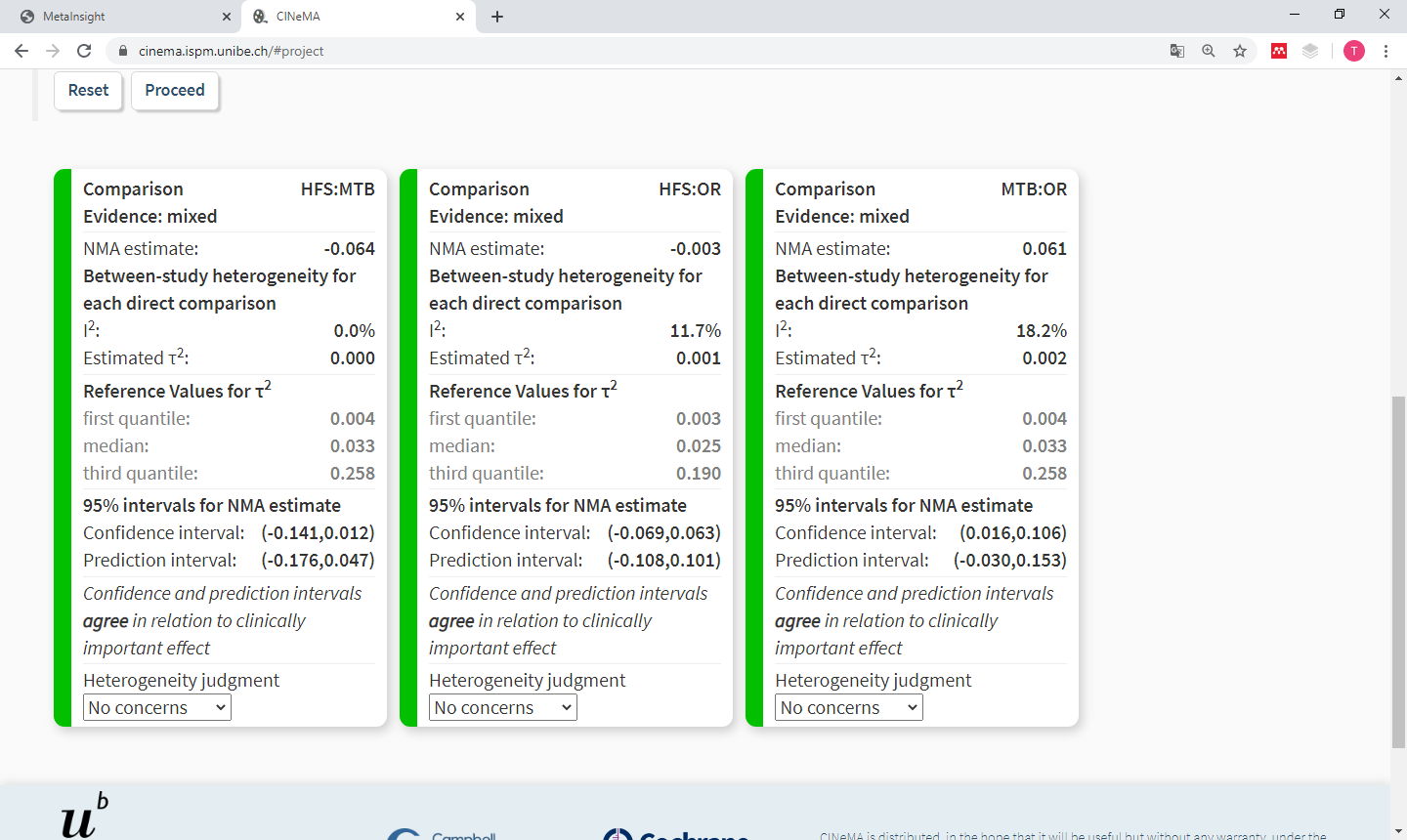


**Appendix S17g.** Confidence in Network Meta-Analysis (CINeMA). Incoherence. Separating indirect from direct evidence. Sub-analysis (M)Q&HPI. *Post-Brushing*


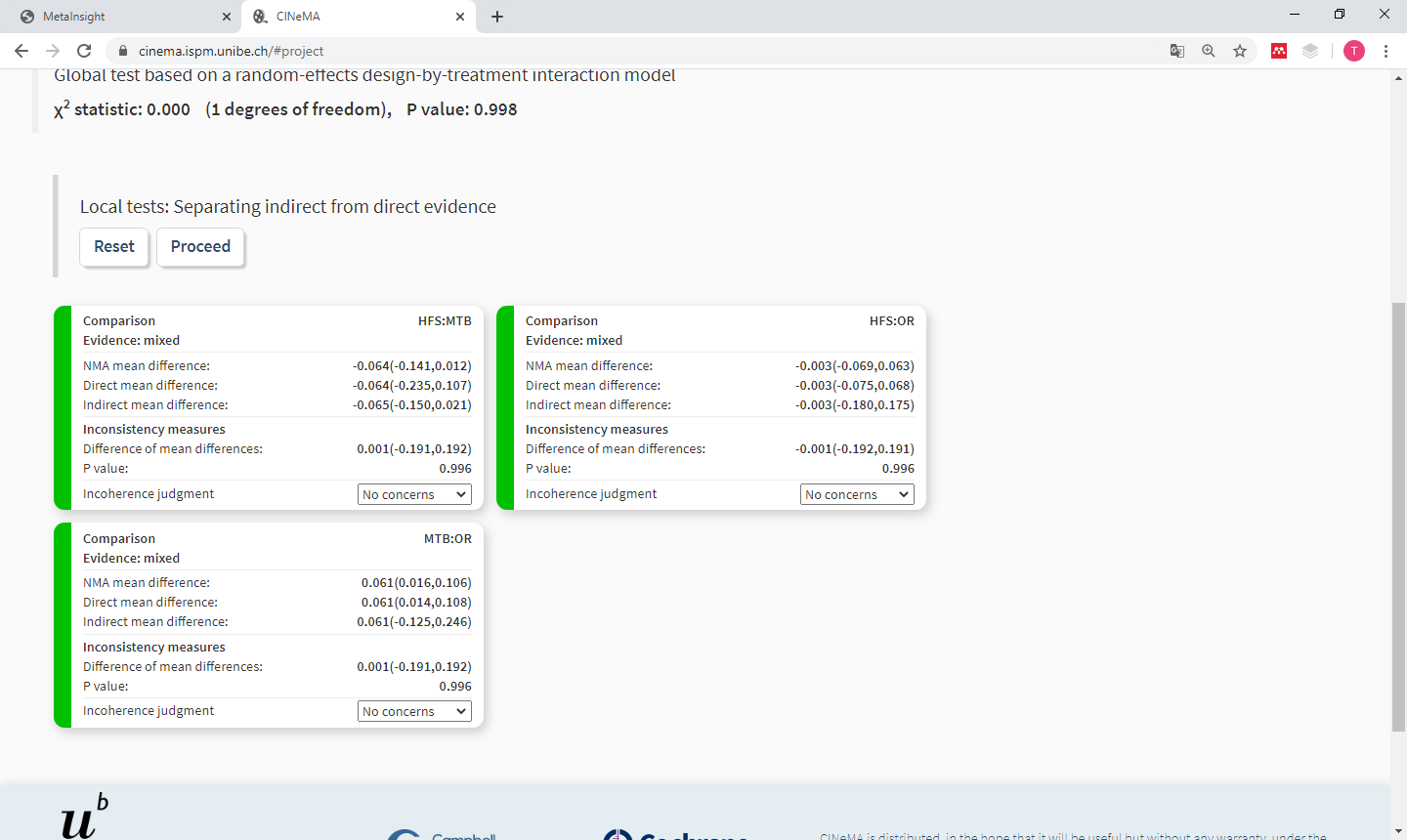


**Appendix S18a.** Confidence in Network Meta-Analysis (CINeMA). Confidence Rating. Sub-analysis RMNPI. *Post-Brushing*

| Comparison | Number of studies | Within-study bias | Reporting bias | Indirectness | Imprecision | Heterogeneity | Incoherence | Confidence rating |
| --- | --- | --- | --- | --- | --- | --- | --- | --- |
| HFS:MTB | x | No concerns | Undetected | No concerns | No concerns | No concerns | No concerns | High |
| HFS:OR | x | No concerns | Undetected | Some concerns | No concerns | No concerns | No concerns | High |
| MTB:OR | x | No concerns | Undetected | No concerns | No concerns | Some concerns | No concerns | High |

**Appendix S18b**. Confidence in Network Meta-Analysis (CINeMA). Risk of bias contributions. The bar chart shows the contributions of each piece of study to the network estimate. Sub-analysis RMNPI. *Post-Brushing*


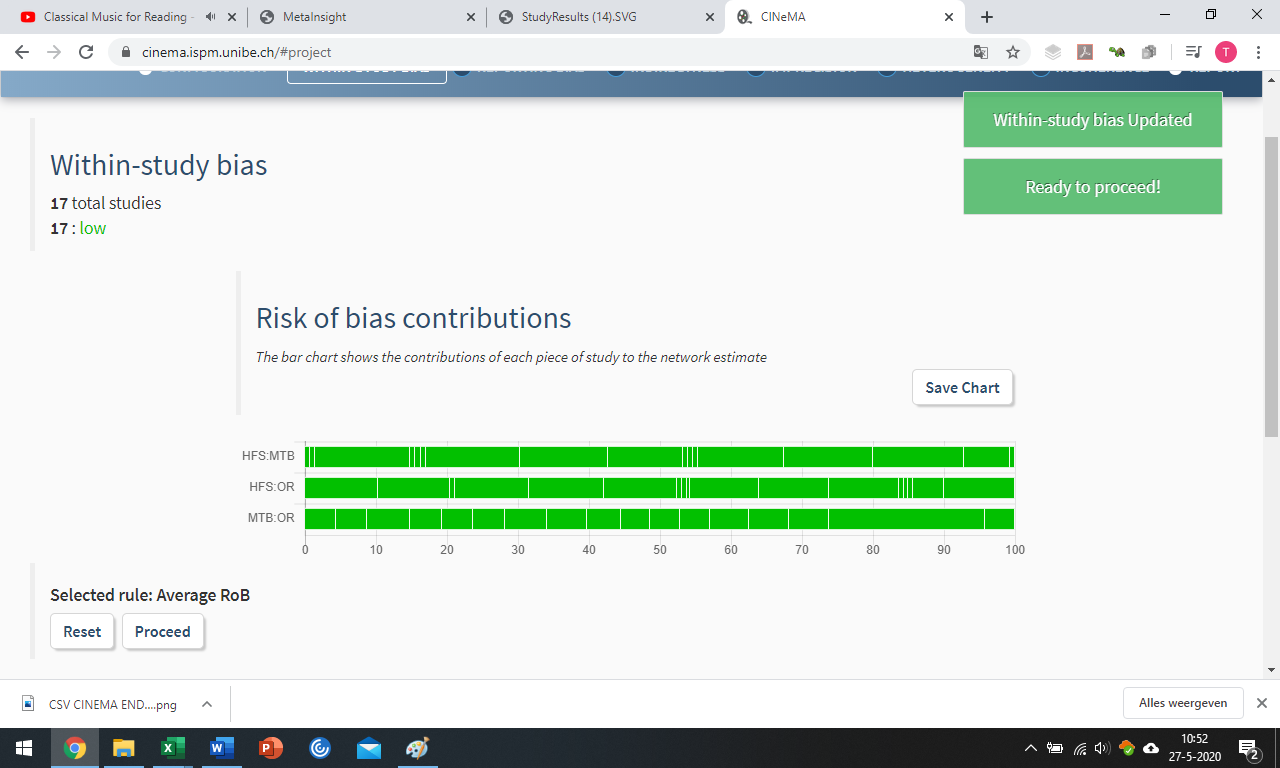


**Appendix S18c**. Confidence in Network Meta-Analysis (CINeMA). Reporting Bias. Sub-analysis RMNPI. *Post-Brushing*


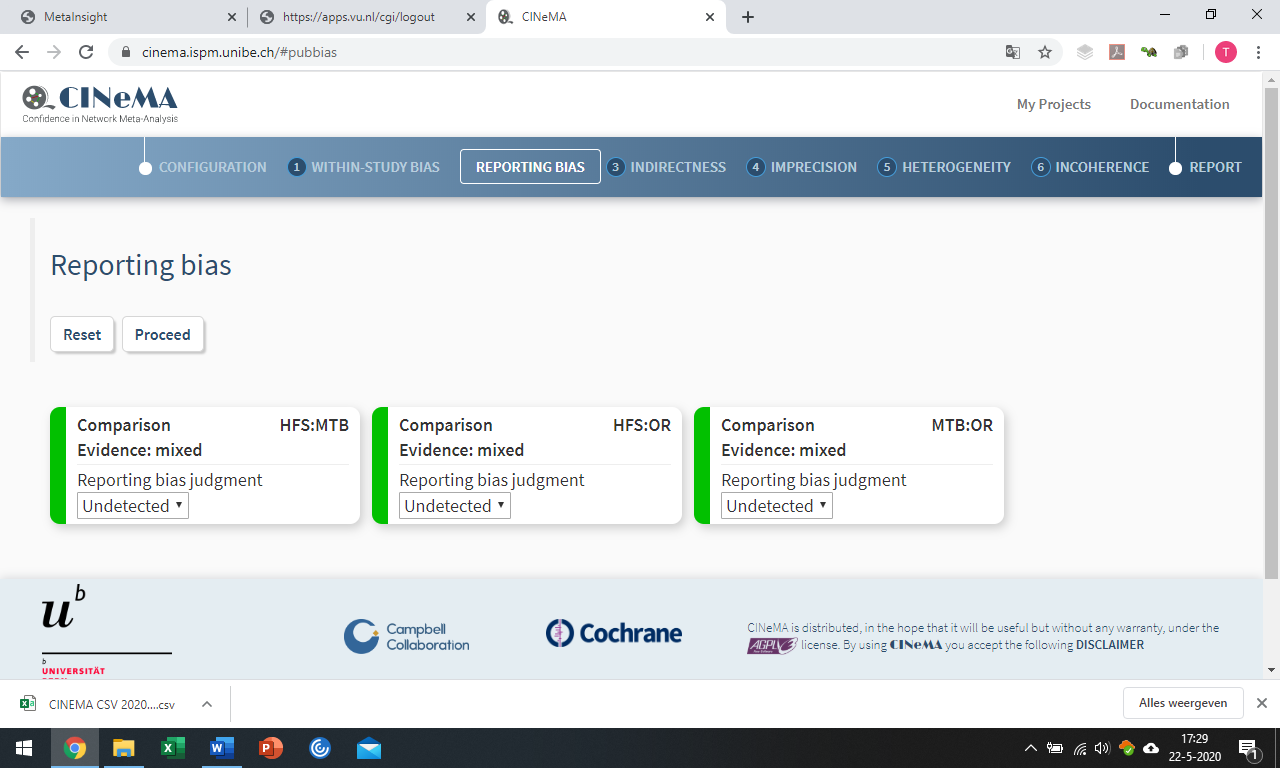


**Appendix S18d.** Confidence in Network Meta-Analysis (CINeMA). Indirectness contributions The bar chart shows the contributions of each study to the network estimate. Sub-analysis RMNPI. *Post-Brushing*


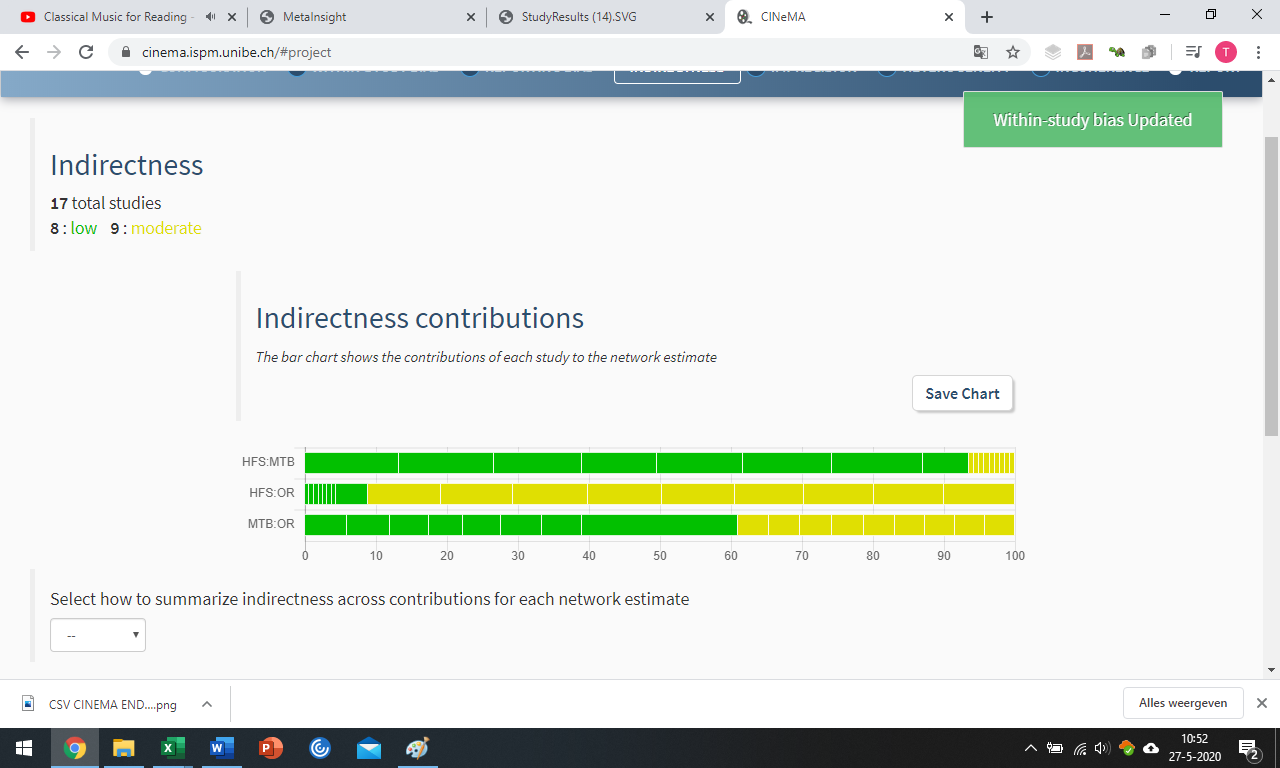


**Appendix S18e.** Confidence in Network Meta-Analysis (CINeMA). Imprecision. Sub-analysis RMNPI. *Post-Brushing*


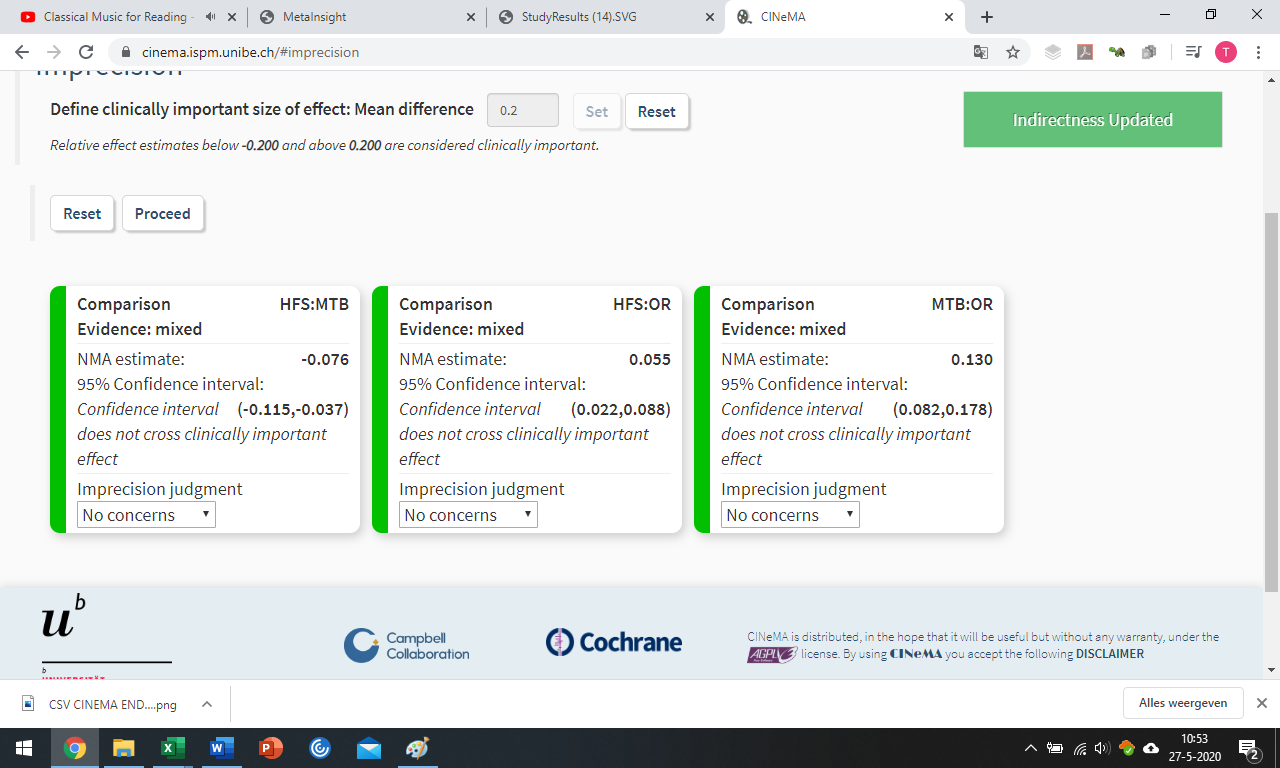


**Appendix S18f**. Confidence in Network Meta-Analysis (CINeMA). Heterogeneity. Sub-analysis RMNPI. *Post-Brushing*


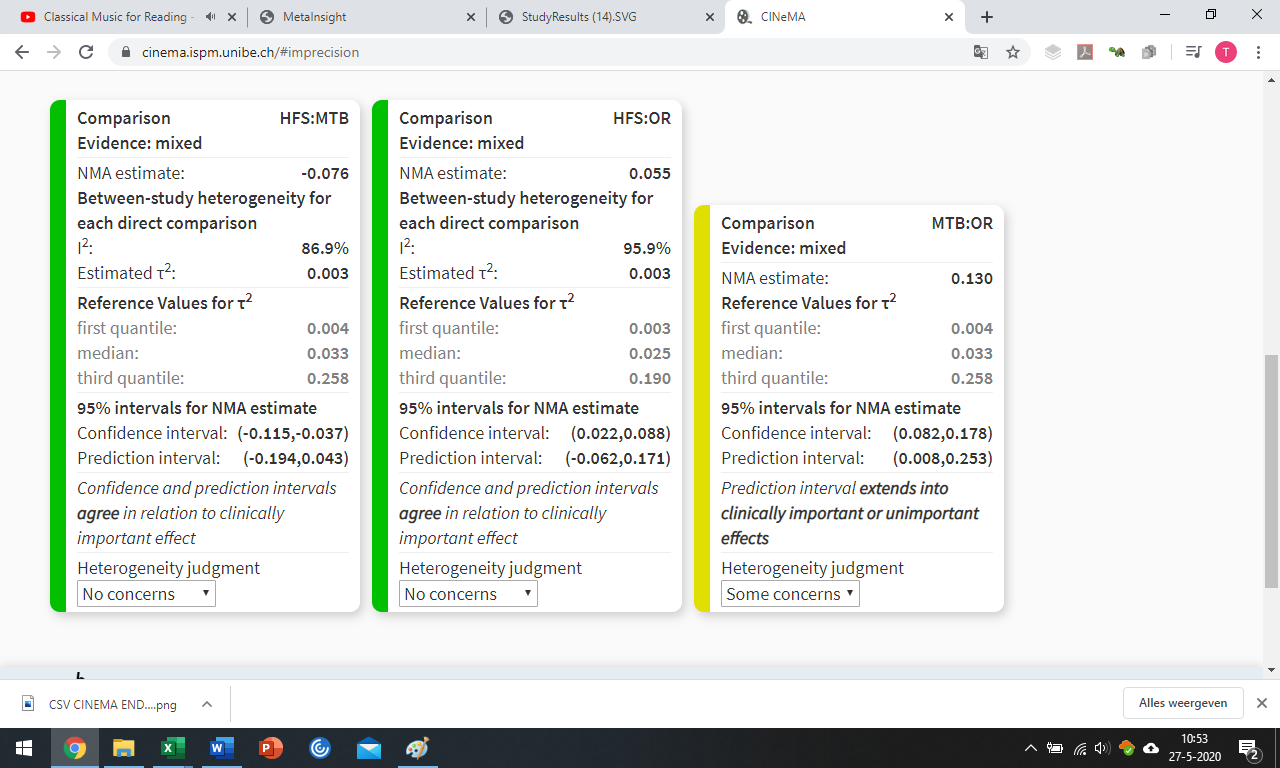


**Appendix S18g**. Confidence in Network Meta-Analysis (CINeMA). Incoherence. Separating indirect from direct evidence. Sub-analysis RMNPI. *Post-Brushing*


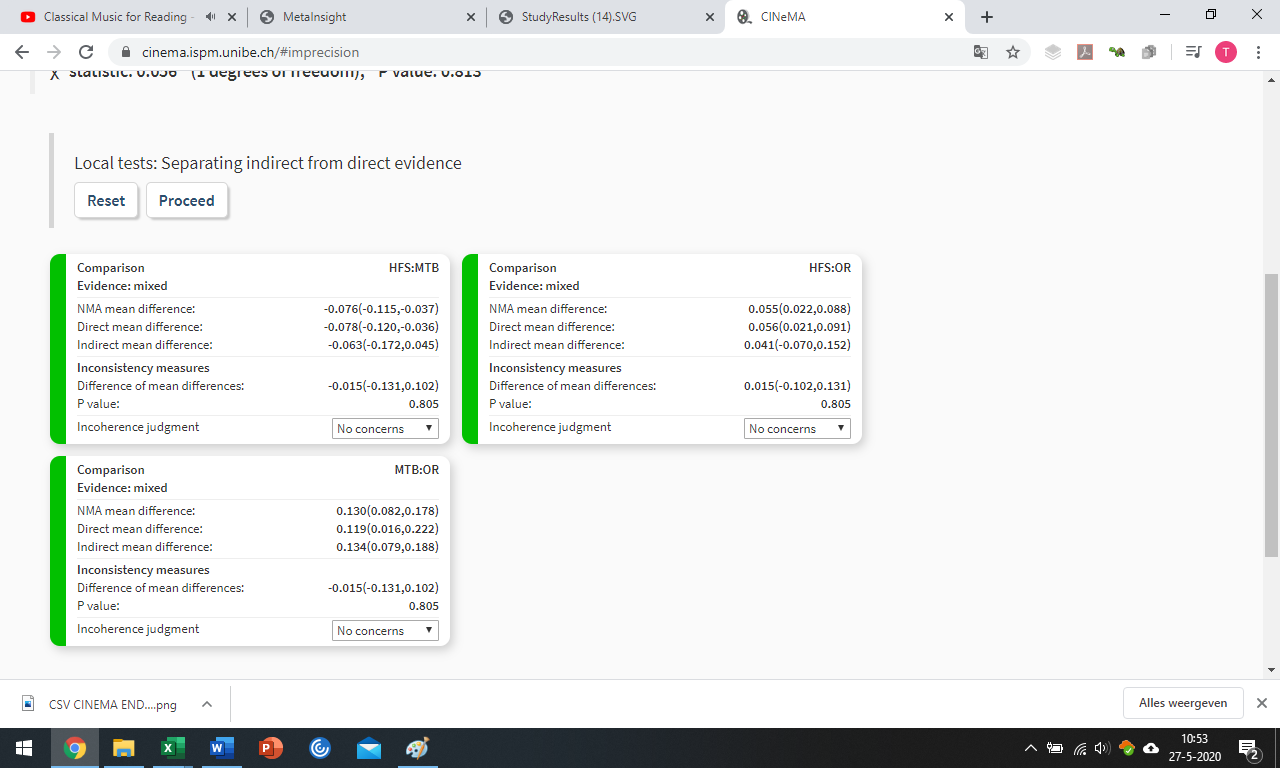


**Appendix S19a.** Confidence in Network Meta-Analysis (CINeMA). Confidence Rating. *Incremental reduction between pre- and post brushing*

| Comparison | Number of studies | Within-study bias | Reporting bias | Indirectness | Imprecision | Heterogeneity | Incoherence | Confidence rating |
| --- | --- | --- | --- | --- | --- | --- | --- | --- |
| HFS:MTB | 8 | No concerns | Undetected | No concerns | No concerns | Major concerns | Major concerns | Moderate |
| HFS:OR | 16 | No concerns | Undetected | Some concerns | No concerns | Major concerns | Major concerns | Moderate |
| MTB:OR | 16 | Some concerns | Undetected | No concerns | No concerns | Major concerns | No concerns | Moderate |

**Appendix S19b.** Confidence in Network Meta-Analysis (CINeMA). Risk of bias contributions. The bar chart shows the contributions of each piece of study to the network estimate. *Incremental reduction between pre- and post brushing*


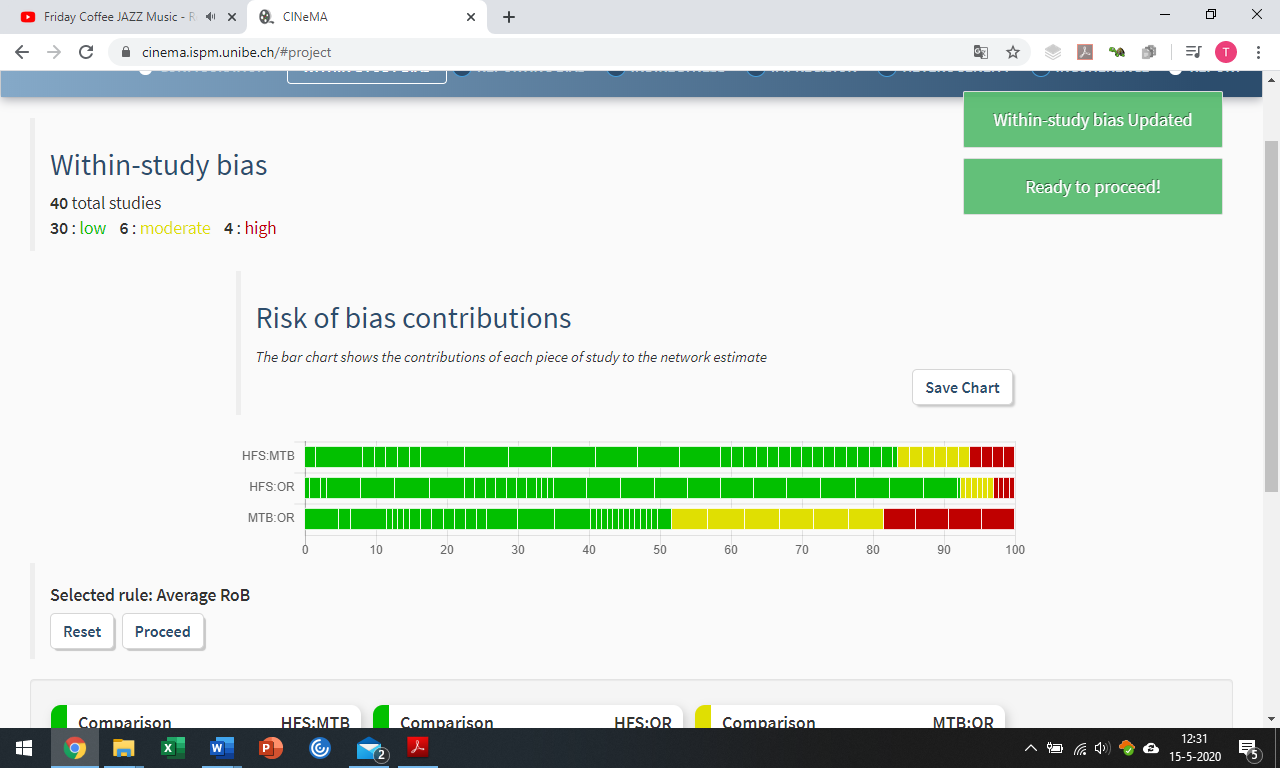


**Appendix S19c.** Confidence in Network Meta-Analysis (CINeMA). Reporting Bias. *Incremental reduction between pre- and post brushing*


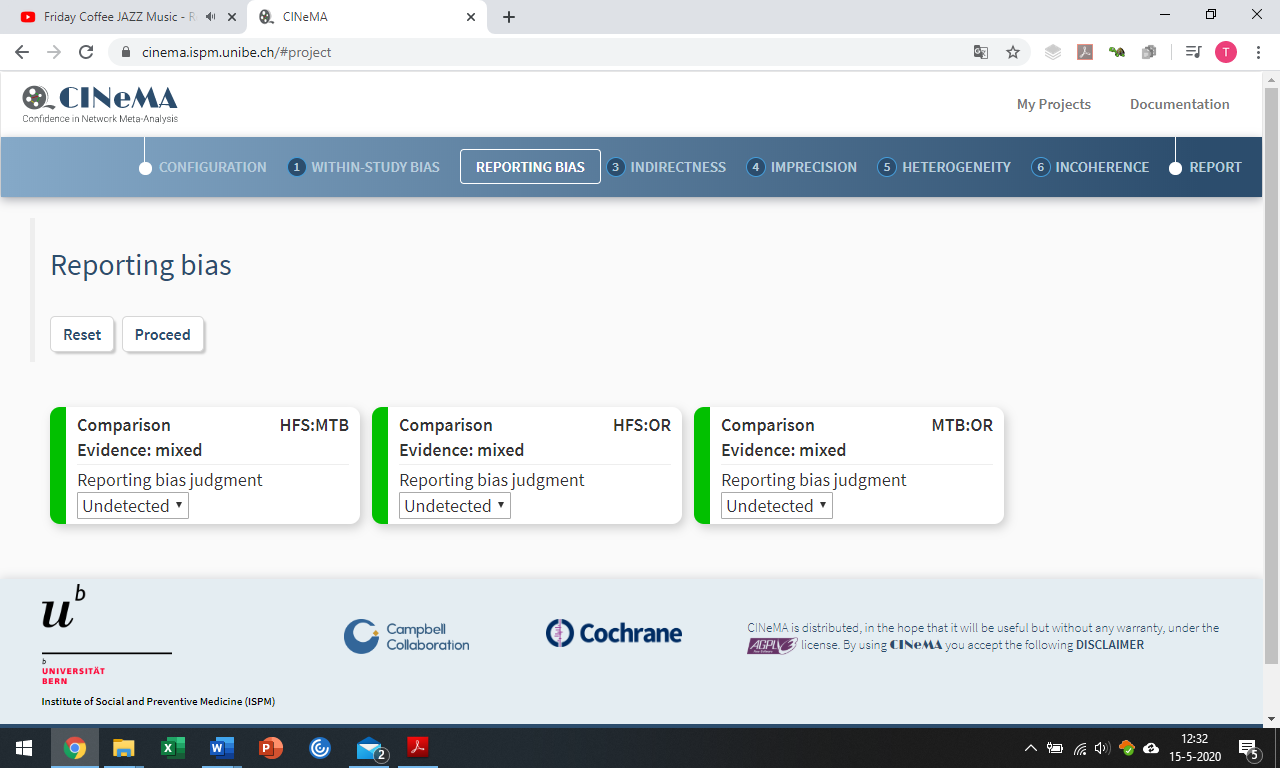


**Appendix S19d.** Confidence in Network Meta-Analysis (CINeMA). Indirectness contributions The bar chart shows the contributions of each study to the network estimate. *Incremental reduction between pre- and post brushing*


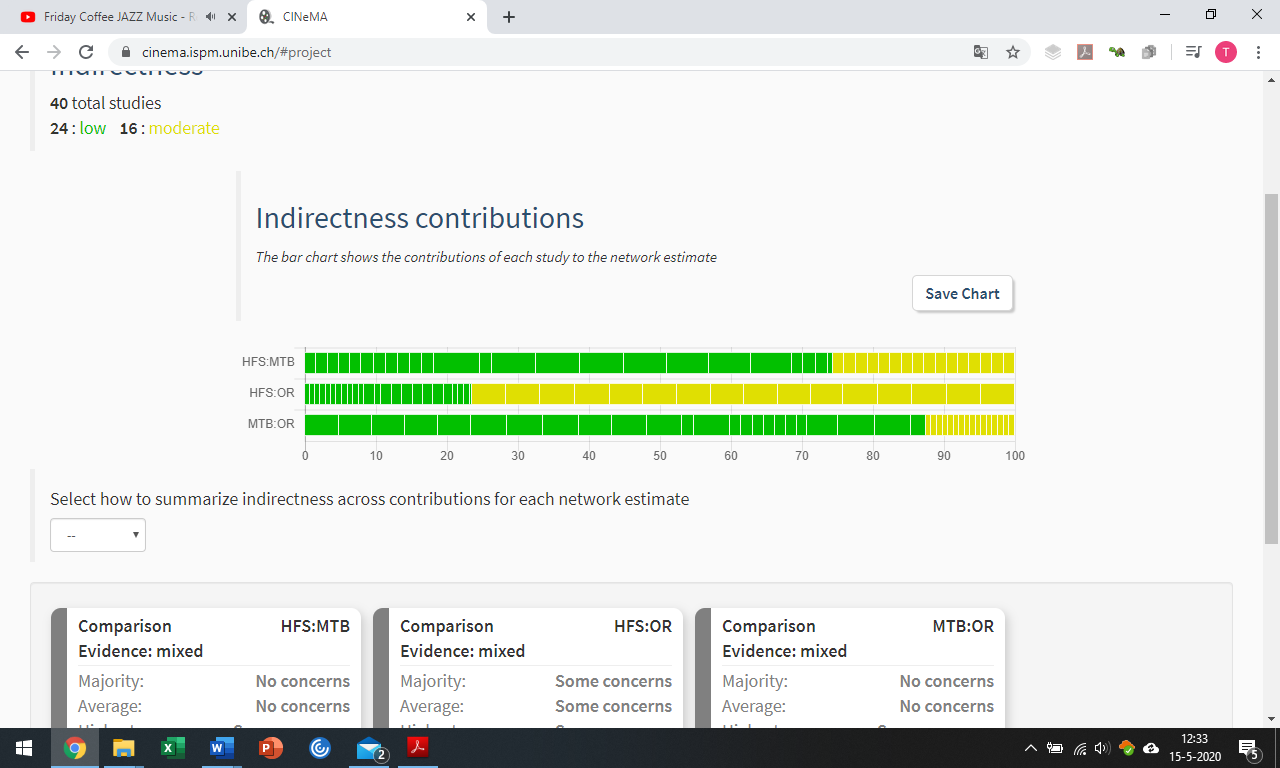


**Appendix S19e**. Confidence in Network Meta-Analysis (CINeMA). Imprecision. *Incremental reduction between pre- and post brushing*


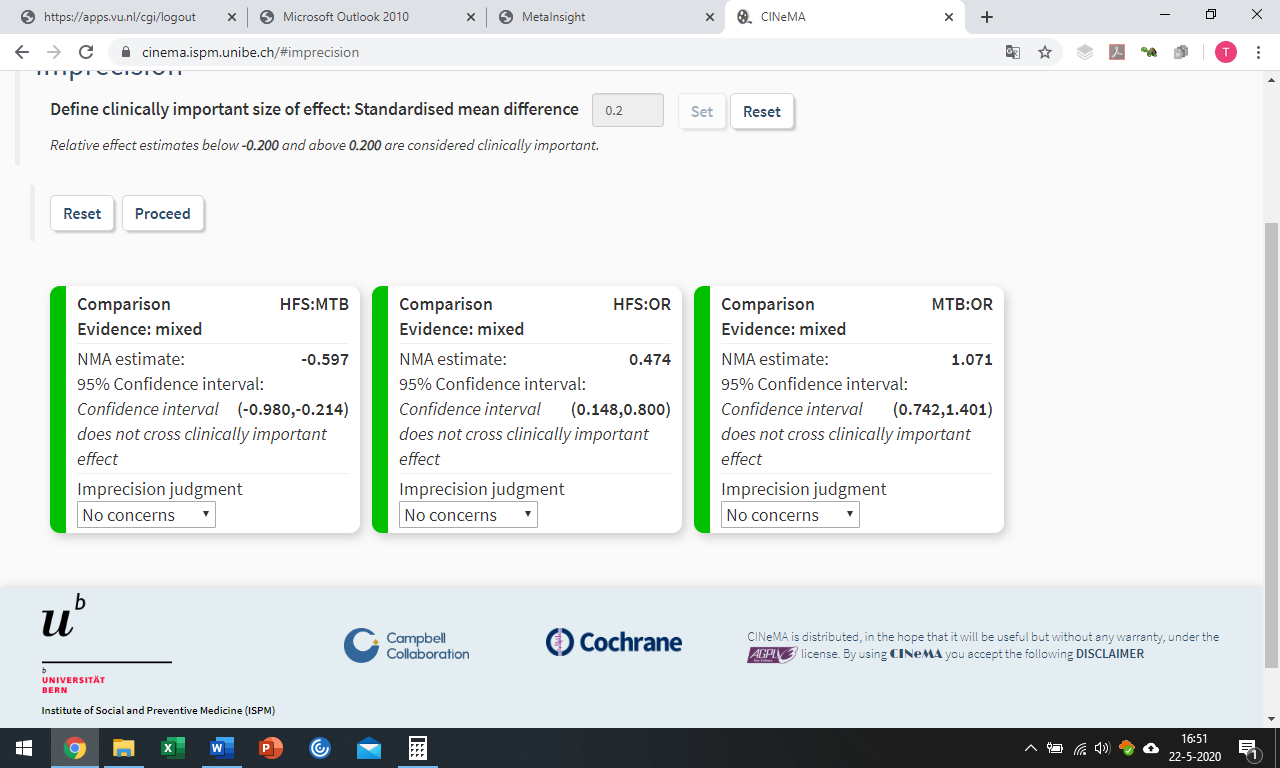


**Appendix S19f.** Confidence in Network Meta-Analysis (CINeMA). Heterogeneity. *Incremental reduction between pre- and post brushing*


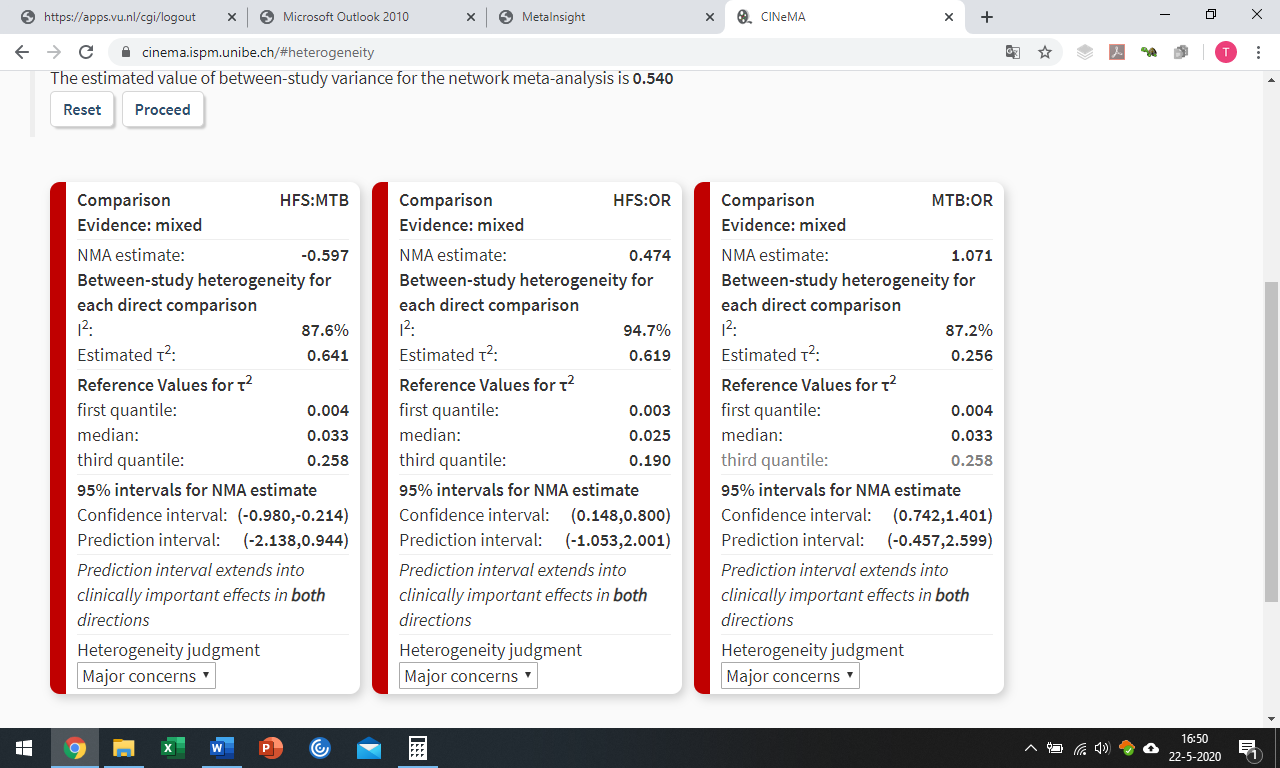


**Appendix S19g.** Confidence in Network Meta-Analysis (CINeMA). Incoherence. Separating indirect from direct evidence. *Incremental reduction between pre- and post brushing*


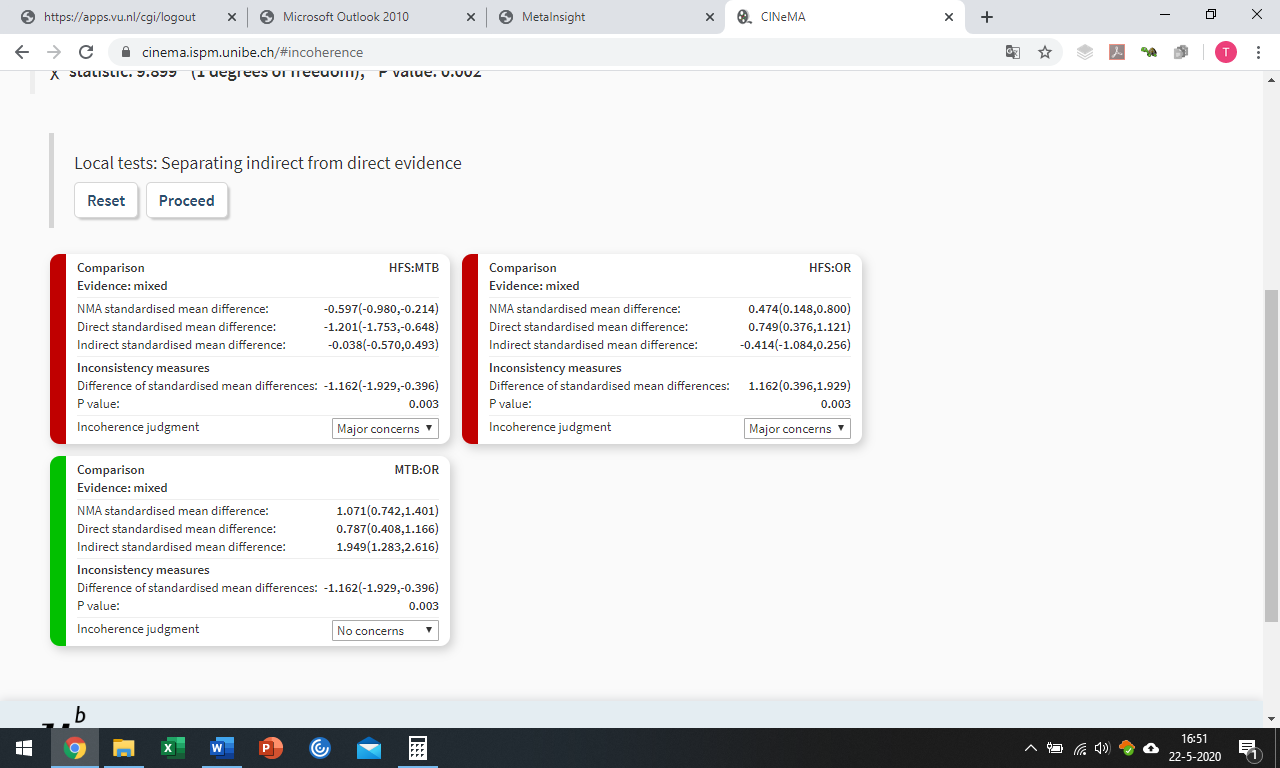


**Appendix S20a**. Confidence in Network Meta-Analysis (CINeMA). Confidence Rating. Sub-analysis (M)Q&HPI. *Incremental reduction between pre- and post brushing*

| Comparison | Number of studies | Within-study bias | Reporting bias | Indirectness | Imprecision | Heterogeneity | Incoherence | Confidence rating |
| --- | --- | --- | --- | --- | --- | --- | --- | --- |
| HFS:MTB | 1 | No concerns | Undetected | No concerns | No concerns | Some concerns | No concerns | High |
| HFS:OR | 4 | No concerns | Undetected | Some concerns | No concerns | Major concerns | No concerns | Moderate |
| MTB:OR | 13 | Some concerns | Undetected | No concerns | No concerns | Some concerns | No concerns | High |

**Appendix 20b.** Confidence in Network Meta-Analysis (CINeMA). Risk of bias contributions. The bar chart shows the contributions of each piece of study to the network estimate. Sub-analysis (M)Q&HPI. *Incremental reduction between pre- and post brushing*


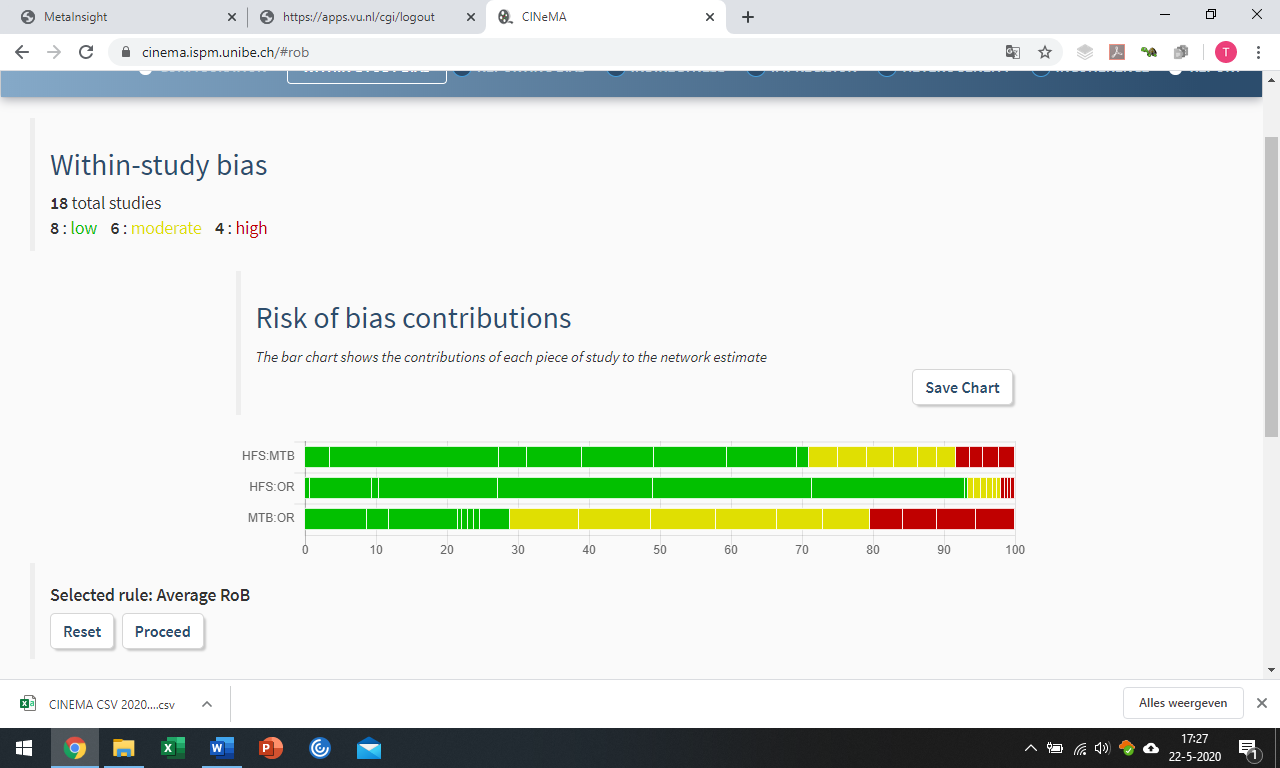


**Appendix S20c.** Confidence in Network Meta-Analysis (CINeMA). Reporting Bias. Sub-analysis (M)Q&HPI. *Incremental reduction between pre- and post brushing*


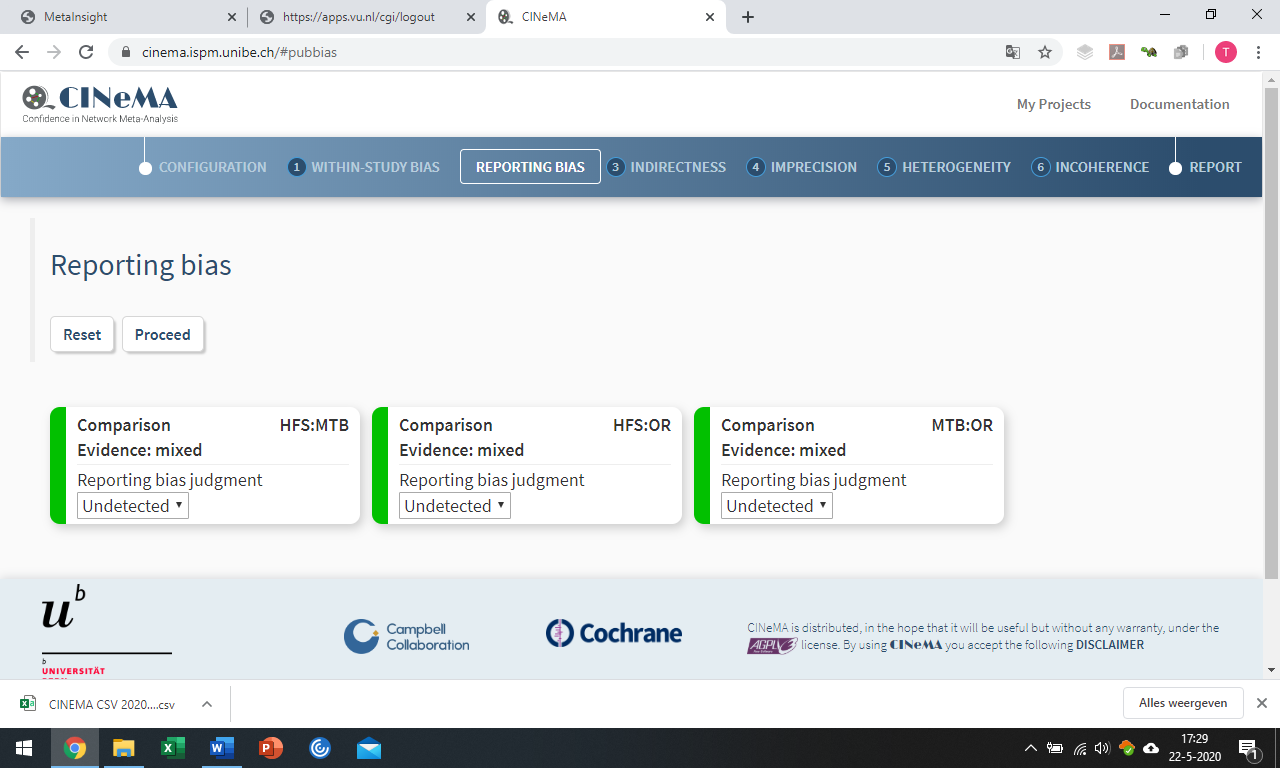


**Appendix S20d.** Confidence in Network Meta-Analysis (CINeMA). Indirectness contributions The bar chart shows the contributions of each study to the network estimate. Sub-analysis (M)Q&HPI. *Incremental reduction between pre- and post brushing*


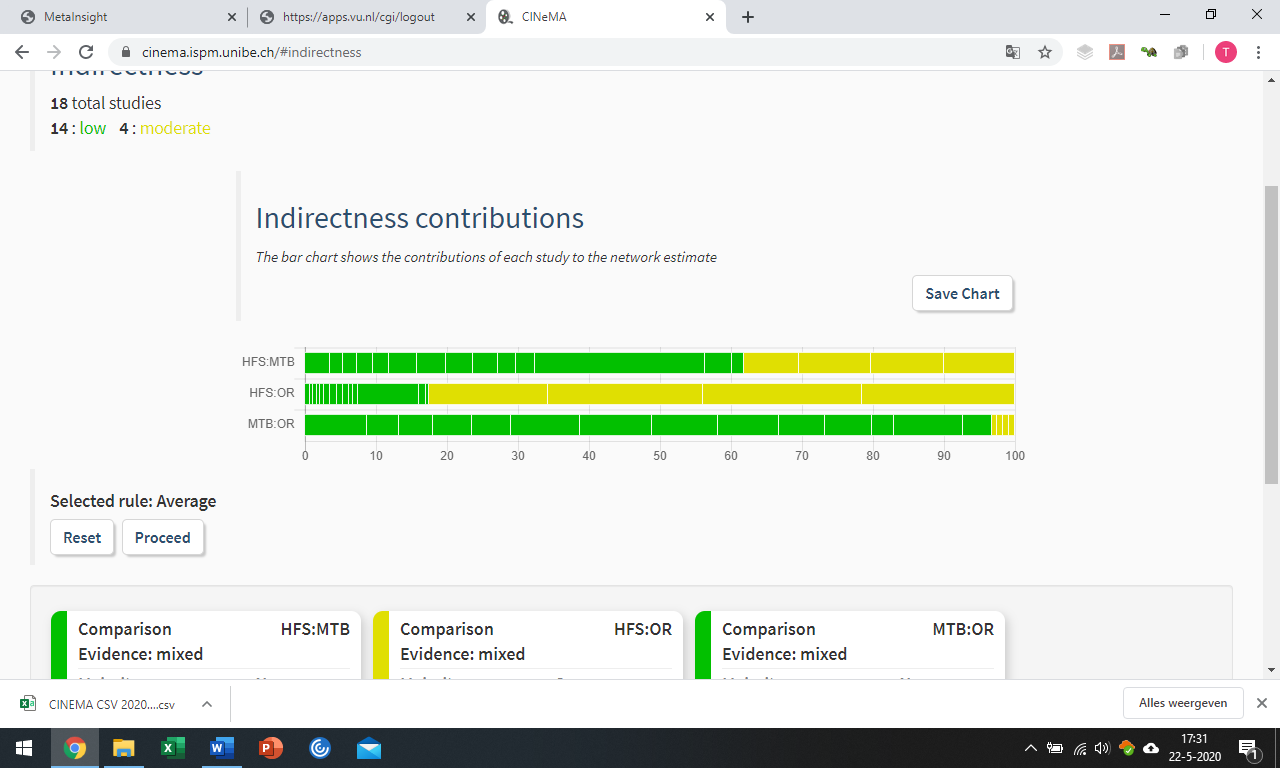


**Appendix S20e.** Confidence in Network Meta-Analysis (CINeMA). Imprecision. Sub-analysis (M)Q&HPI. *Incremental reduction between pre- and post brushing*


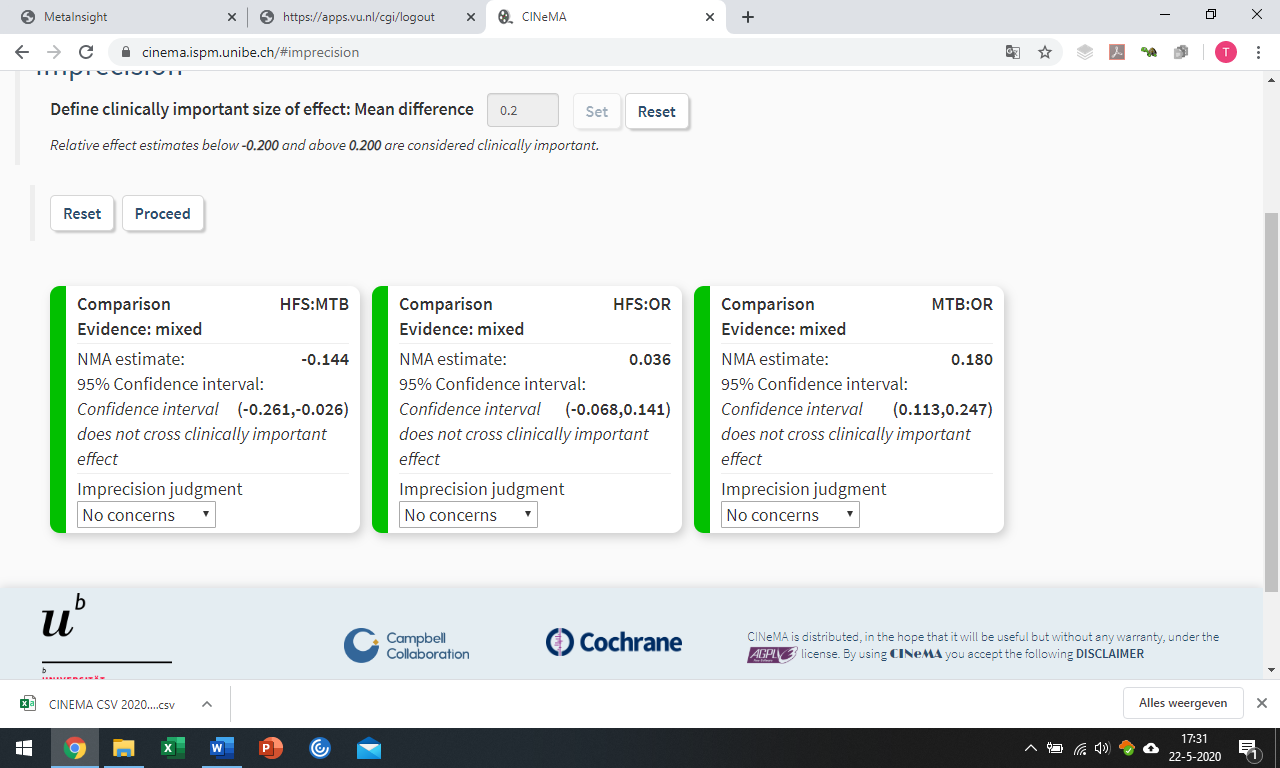


**Appendix S20f.** Confidence in Network Meta-Analysis (CINeMA). Heterogeneity. Sub-analysis (M)Q&HPI. *Incremental reduction between pre- and post brushing*


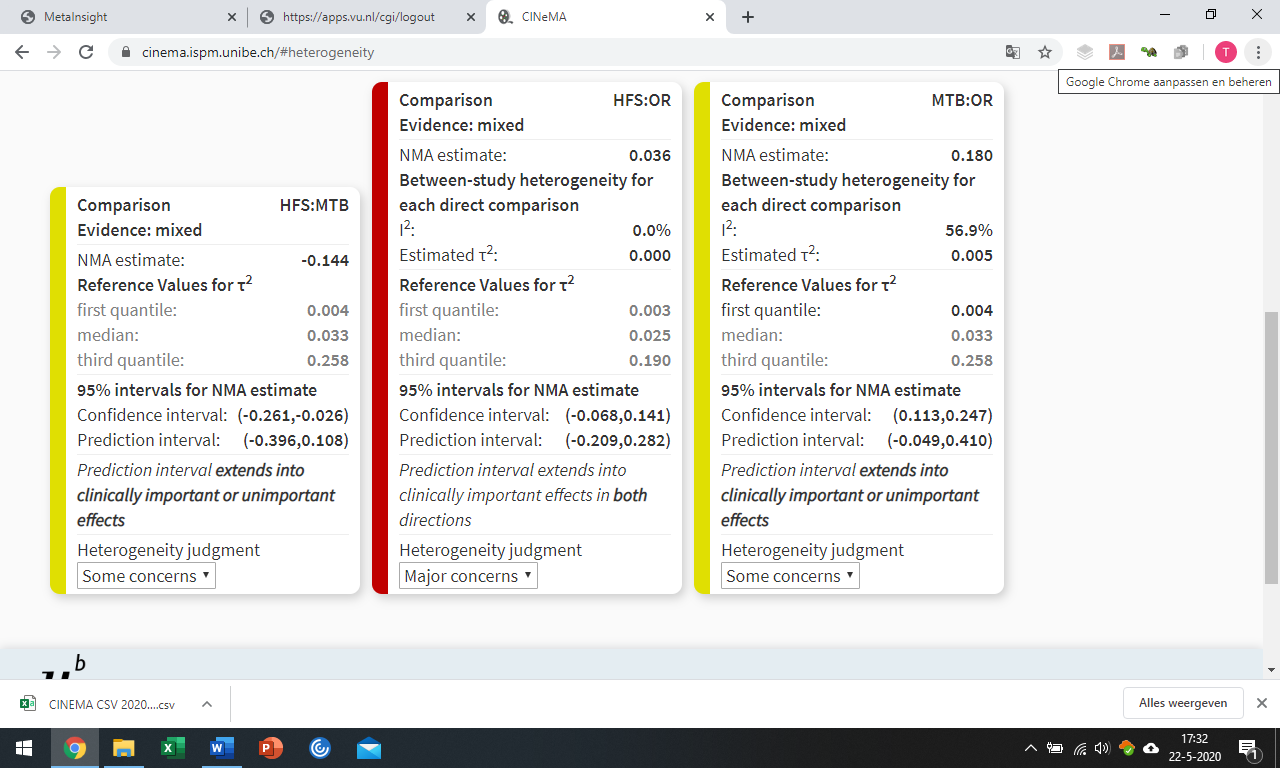


**Appendix S20g.** Confidence in Network Meta-Analysis (CINeMA). Incoherence. Separating indirect from direct evidence. Sub-analysis (M)Q&HPI. *Incremental reduction between pre- and post brushing*


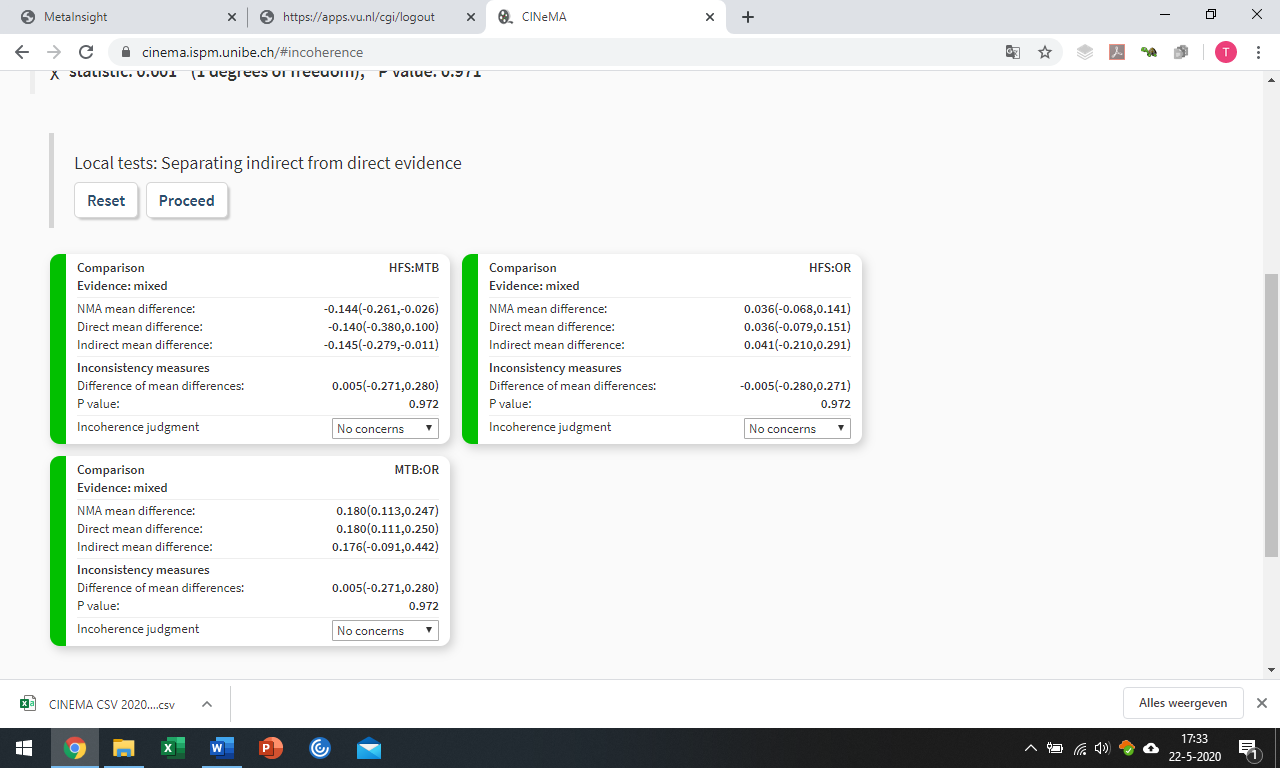


**Appendix S21a**. Confidence in Network Meta-Analysis (CINeMA). Confidence Rating. Sub-analysis RMNPI. *Incremental reduction between pre- and post brushing*

| Comparison | Number of studies | Within-study bias | Reporting bias | Indirectness | Imprecision | Heterogeneity | Incoherence | Confidence rating |
| --- | --- | --- | --- | --- | --- | --- | --- | --- |
| HFS:MTB | x | No concerns | Undetected | No concerns | No concerns | No concerns | No concerns | High |
| HFS:OR | x | No concerns | Undetected | Some concerns | No concerns | No concerns | No concerns | High |
| MTB:OR | x | No concerns | Undetected | No concerns | No concerns | Some concerns | Some concerns | Moderate |

**Appendix S21b.** Confidence in Network Meta-Analysis (CINeMA). Risk of bias contributions. The bar chart shows the contributions of each piece of study to the network estimate. Sub-analysis RMNPI. *Incremental reduction between pre- and post brushing*


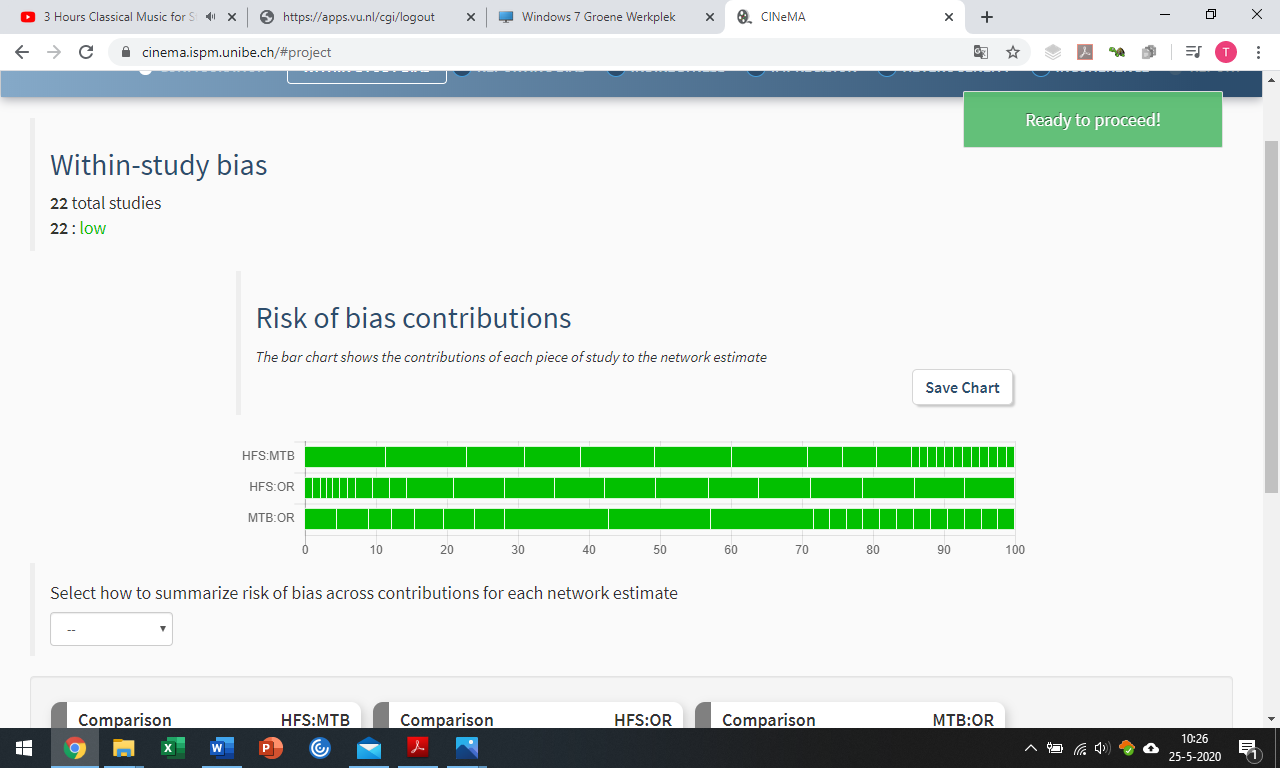


**Appendix S21c.** Confidence in Network Meta-Analysis (CINeMA). Reporting Bias. Sub-analysis RMNPI. *Incremental reduction between pre- and post brushing*


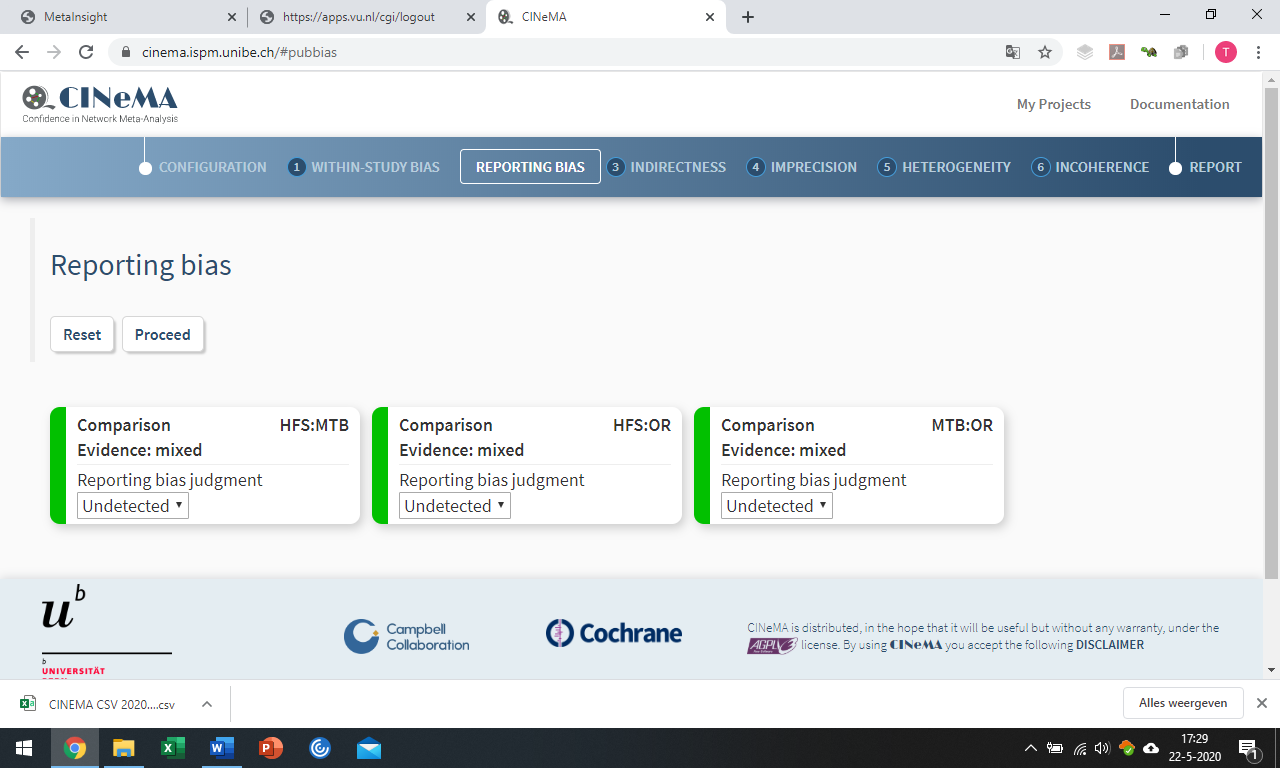


**Appendix S21d.** Confidence in Network Meta-Analysis (CINeMA). Indirectness contributions The bar chart shows the contributions of each study to the network estimate. Sub-analysis RMNPI. *Incremental reduction between pre- and post brushing*


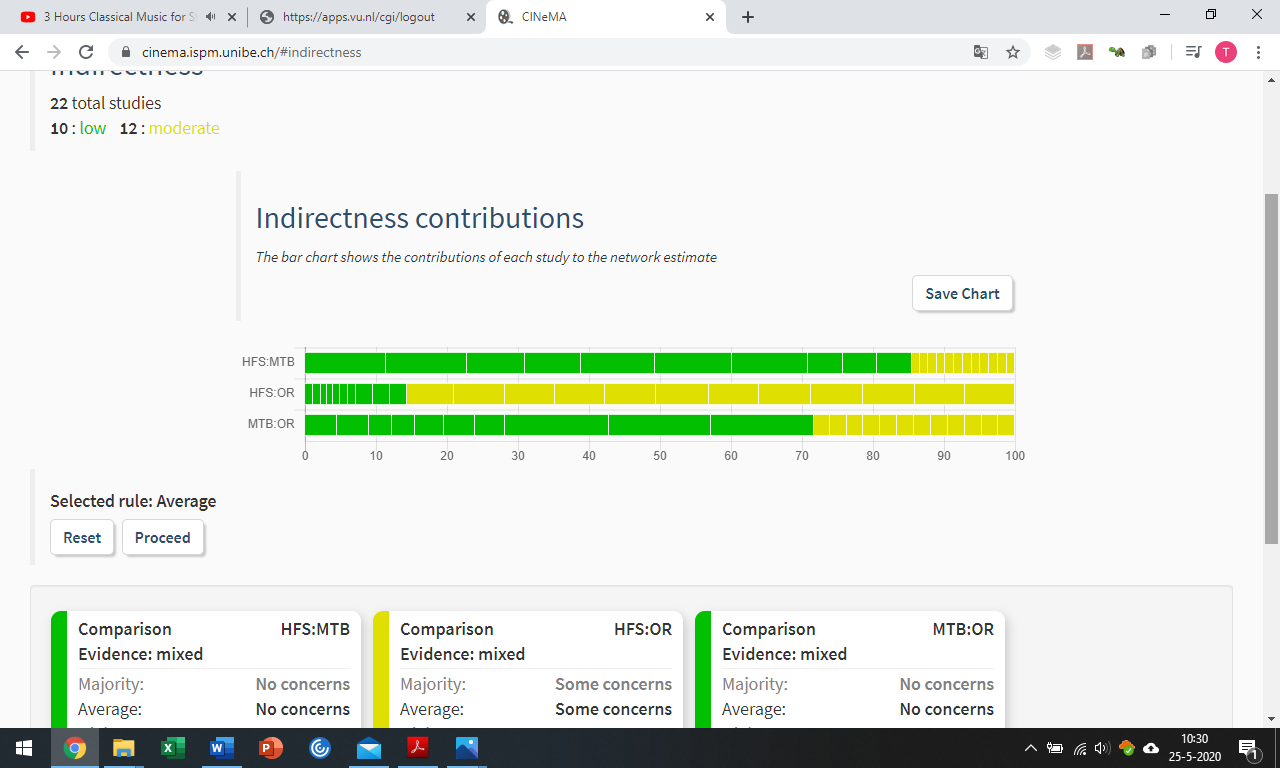


**Appendix S21e.** Confidence in Network Meta-Analysis (CINeMA). Imprecision. Sub-analysis RMNPI. *Incremental reduction between pre- and post brushing*


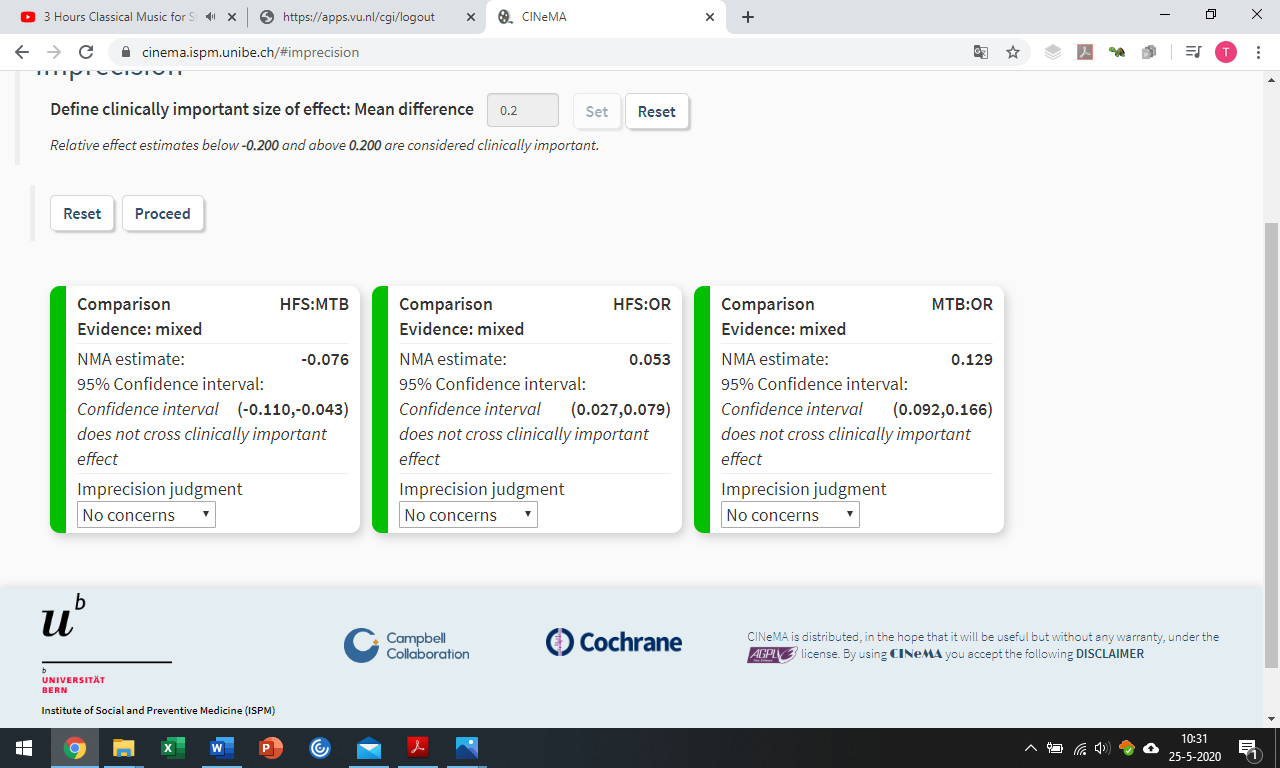


**Appendix S21f.** Confidence in Network Meta-Analysis (CINeMA). Heterogeneity. Sub-analysis RMNPI. *Incremental reduction between pre- and post brushing*


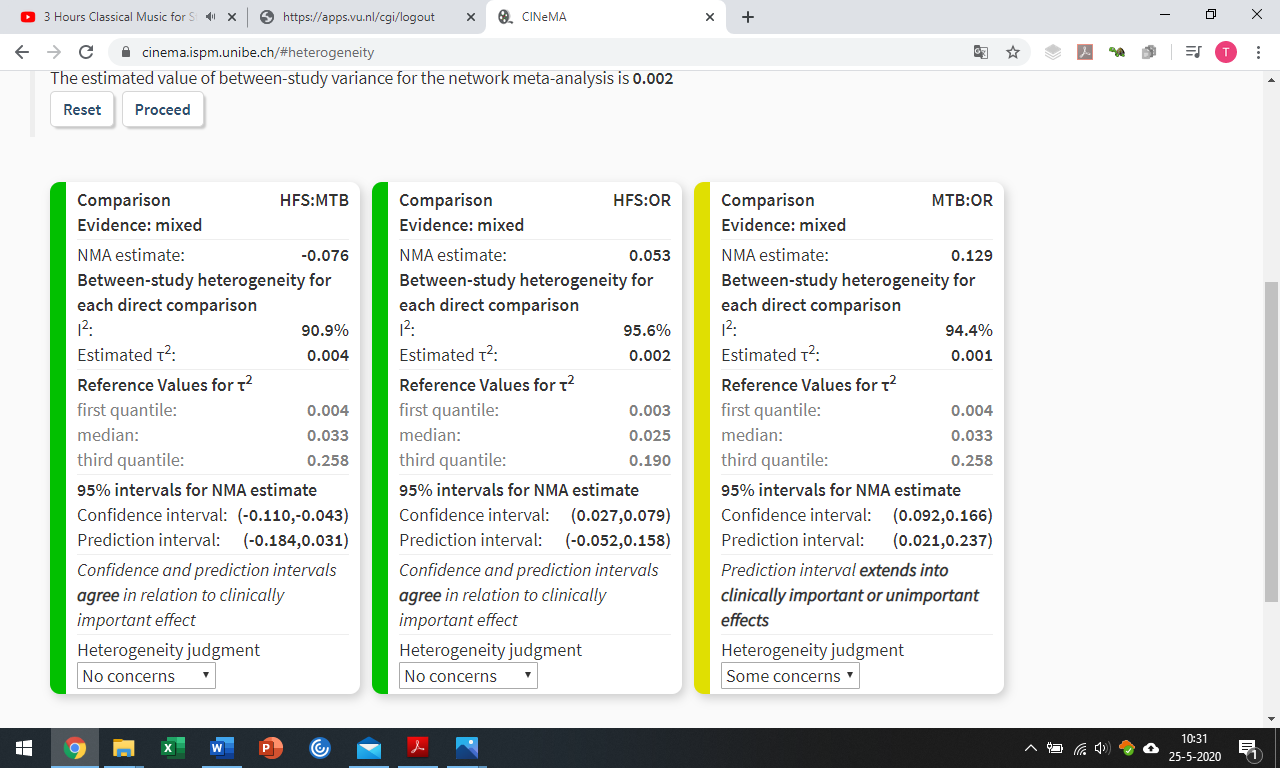


**
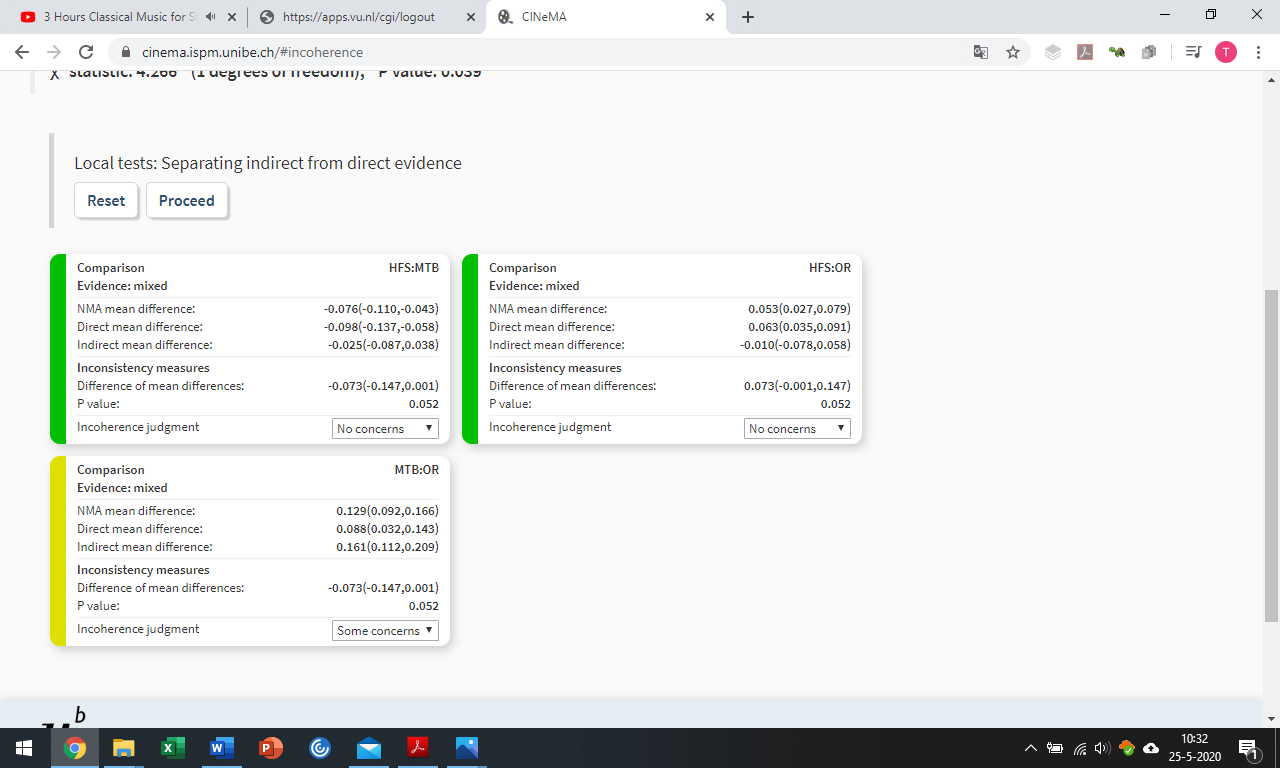
Appendix S21g.** Confidence in Network Meta-Analysis (CINeMA). Incoherence. Separating indirect from direct evidence. Sub-analysis RMNPI. *Incremental reduction between pre- and post brushing*

**Appendix S22.**

References used in the online appendices

1. Adam R, Erb J, Grender J. Randomized controlled trial assessing plaque removal of an oscillating-rotating electric toothbrush with micro-vibrations. *Int Dent J*. 2020;70:S22-S27. doi:10.1111/idj.12568

2. Heasman PA, Stacey F, Heasman L, Sellers P, Macgregor IDM, Kelly PJ. A comparative study of the Philips HP 735, Braun/Oral B D7 and the Oral B 35 Advantage toothbrushes. *J Clin Periodontol*. 1999;26:85-90. doi:10.1034/j.1600-051X.1999.260204.x

3. Klukowska M, Grender JM, Timm H. A single-brushing study to compare plaque removal efficacy of a new power brush to an ADA reference manual toothbrush. *Am J Dent*. 2012;25(SPEC. ISSUE A):10A-13A

4. Kulkarni P, Singh DK, Jalaluddin M. Comparison of efficacy of manual and powered toothbrushes in plaque control and gingival inflammation: A clinical study among the population of east indian region. *J Int Soc Prev Community Dent*. 2017;7:168-174. doi:10.4103/jispcd.JISPCD_133_17

5. Kurtz B, Reise M, Klukowska M, Grender JM, Timm H, Sigusch BW. A randomized clinical trial comparing plaque removal efficacy of an oscillating–rotating power toothbrush to a manual toothbrush by multiple examiners. *Int J Dent Hyg*. 2016;14:278-283. doi:10.1111/idh.12225

6. Moritis K, Delaurenti M, Johnson MR, Berg J, Boghosian AA. Comparison of the Sonicare Elite and a manual toothbrush in the evaluation of plaque reduction. *Am J Dent*. 2002;15 Spec No:23B-25B. http://www.ncbi.nlm.nih.gov/pubmed/12516678. Accessed June 1, 2021

7. Pizzo G, Licata ME, Pizzo I, D’Angelo M. Plaque removal efficacy of power and manual toothbrushes: A comparative study. *Clin Oral Investig*. 2010;14:375-381. doi:10.1007/s00784-009-0303-3

8. Re D, Augusti G, Battaglia D, Giannì AB, Augusti D. Is a new sonic toothbrush more effective in plaque removal than a manual toothbrush? *Eur J Paediatr Dent*. 2015;16:13-18.

9. Renton-Harper P, Addy M, Newcombe RG. Plaque removal with the uninstructed use of electric toothbrushes: Comparison with a manual brush and toothpaste slurry. *J Clin Periodontol*. 2001;28:325-330. doi:10.1034/j.1600-051x.2001.028004325.x

10. Robinson PJ, Maddalozzo D, Breslin S. A Six-Month Clinical Comparison of the Efficacy of the Sonicare® and the Braun Oral-B® Electric Toothbrushes on Improving Periodontal Health in Adult Periodontitis Patients. *J Clin Dent*. 1997;8:4-9.

11. Rosema NAM, Adam R, Grender JM, Van der Sluijs E, Supranoto SC, Van der Weijden GA. Gingival abrasion and recession in manual and oscillating-rotating power brush users. *Int J Dent Hyg*. 2014;12:257-266. doi:10.1111/idh.12085

12. Yankell SL, Emling RC. A Thirty-Day Safety and Efficacy Evaluation of the Rowenta, Braun and Sonicare Powered Toothbrushes and a Manual Toothbrush. *J Clin Dent*. 1997;8(4):120-123.. http://www.ncbi.nlm.nih.gov/pubmed/26630722. Accessed June 1, 2021

13. Van der Weijden GA, Timmerman MF, Reijerse E, Snoek CM, Van der Velden U. Comparison of an oscillating/rotating electric toothbrush and a “sonic” toothbrush in plaque-removing ability. A professional toothbrushing and supervised brushing study. *J Clin Periodontol*. 1996;23:407-411. doi:10.1111/j.1600-051x.1996.tb00565.x

14. Williams K, Rapley K, Huan J, et al. A study comparing the plaque removal efficacy of an advanced rotation-oscillation power toothbrush to a new sonic toothbrush. *J Clin Dent*. 2008;19:154-158. http://www.ncbi.nlm.nih.gov/pubmed/19278087. Accessed June 1, 2021.

15. Williams K, Rapley K, Haun J, et al. Comparison of rotation/oscillation and sonic power toothbrushes on plaque and gingivitis for 10 weeks. *Am J Dent*. 2009;22:345-349.

16. Ayad F, Petrone DM, Wachs GN, Mateo LR, Chaknis P, Panagakos F. Comparative efficacy of a specially engineered sonic powered toothbrush with unique sensing and control technologies to two commercially available power toothbrushes on established plaque and gingivitis. *J Clin Dent*. 2012;23:A5-10.

17. Biesbrock AR, Bartizek RD, Walters PA, et al. Clinical evaluations of plaque removal efficacy: An advanced rotating-oscillating power toothbrush versus a sonic toothbrush. *J Clin Dent*. 2007;18:106-111.

18. Biesbrock AR, Walters PA, Bartizek RD, Goyal CR, Qaqish JG. Plaque removal efficacy of an advanced rotation-oscillation power toothbrush versus a new sonic toothbrush. *Am J Dent*. 2008;21:185-188.

19. Gallob JT, Lynch M, Charles C, et al. A randomized trial of ethyl lauroyl arginate-containing mouthrinse in the control of gingivitis. *J Clin Periodontol*. 2015;42:740-747. doi:10.1111/jcpe.12428

20. Goyal CR, Qaqish J, He T, Walters P, Grender J, Biesbrock AR. A randomized 12-week study to compare the gingivitis and plaque reduction benefits of a rotation-oscillation power toothbrush and a sonic power toothbrush. *J Clin Dent*. 2009;20:93-98.

21. Nathoo S, Mankodi S, Mateo LR, Chaknis P, Panagakos F. A Clinical study comparing the supragingival plaque and gingivitis efficacy of a specially engineered sonic powered toothbrush with unique sensing and control technologies to a commercially available manual flat-trim toothbrush. *J Clin Dent*. 2012;23(SPEC. ISS. A):A11-6

22. Nathoo S, Mateo LR, Chaknis P, et al. Efficacy of two different toothbrush heads on a sonic power toothbrush compared to a manual toothbrush on established gingivitis and plaque. *J Clin Dent*. 2014;25:65-70.

23. Sharma NC, Galustians J, Qaqish J, Cugini M. A comparison of two electric toothbrushes with respect to plaque removal and subject preference. *Am J Dent*. 1998;11:S29-33.

24. Sharma NC, Goyal CR, Qaqish JG, Cugini MA, Thompson MC, Warren PR. Single-use plaque removal efficacy of three power toothbrushes. *J Dent*. 2005;33S1:11-15.

25. Sharma NC, Lyle DM, Qaqish JG, Galustians J. Evaluation of the plaque removal efficacy of three power toothbrushes. *J Int Acad Periodontol*. 2006;8:83-88.

26. Sharma NC, Qaqish J, Klukowska M, Grender J, Rooney J. The plaque removal efficacy of a novel power brush head. *J Clin Dent*. 2011;22:19-22.

27. Strate J, Cugini MA, Warren PR, Qaqish JG, Galustians HJ, Sharma NC. A comparison of the plaque removal efficacy of two power toothbrushes: Oral-B Professional Care Series versus Sonicare Elite. *Int Dent J*. 2005;55:151-156. doi:10.1111/j.1875-595X.2005.tb00312.x

28. Terézhalmy GT, Bartizek RD, Biesbrock AR. Relative Plaque Removal of Three Toothbrushes in a Nine-Period Crossover Study. *J Periodontol*. 2005;76:2230-2235. doi:10.1902/jop.2005.76.12.2230
